# Multilayered Computational Framework for Designing Peptide Inhibitors of HVEM-LIGHT Interaction

**DOI:** 10.1021/acs.jpcb.4c02255

**Published:** 2024-07-03

**Authors:** Piotr Ciura, Pamela Smardz, Marta Spodzieja, Adam K. Sieradzan, Pawel Krupa

**Affiliations:** † Faculty of Chemistry, Fahrenheit Union of Universities in Gdańsk, University of Gdańsk, Baż̇yńskiego 8, 80-309 Gdansḱ, Poland; ‡ Institute of Physics, Polish Academy of Sciences, Al. Lotnikow 32/46, 02-668 Warsaw, Poland

## Abstract

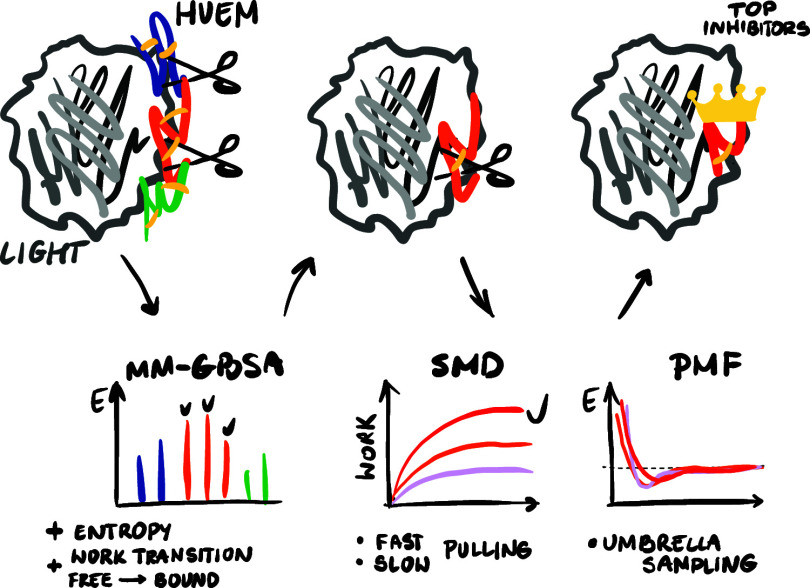

The herpesvirus entry mediator (HVEM) and its ligand LIGHT play crucial roles in immune system regulation, including T-cell proliferation, B-cell differentiation, and immunoglobulin secretion. However, excessive T-cell activation can lead to chronic inflammation and autoimmune diseases. Thus, inhibiting the HVEM-LIGHT interaction emerges as a promising therapeutic strategy for these conditions and in preventing adverse reactions in organ transplantation. This study focused on designing peptide inhibitors, targeting the HVEM-LIGHT interaction, using molecular dynamics (MD) simulations of 65 peptides derived from HVEM. These peptides varied in length and disulfide-bond configurations, crucial for their interaction with the LIGHT trimer. By simulating 31 HVEM domain variants, including the full-length protein, we assessed conformational changes upon LIGHT binding to understand the influence of HVEM segments and disulfide bonds on the binding mechanism. Employing multitrajectory microsecond-scale, all-atom MD simulations and molecular mechanics with generalized Born and surface area (MM-GBSA) binding energy estimation, we identified promising CRD2 domain variants with high LIGHT affinity. Notably, point mutations in these variants led to a peptide with a single disulfide bond (C58–C73) and a K54E substitution, exhibiting the highest binding affinity. The importance of the CRD2 domain and Cys58–Cys73 disulfide bond for interrupting HVEM-LIGHT interaction was further supported by analyzing truncated CRD2 variants, demonstrating similar binding strengths and mechanisms. Further investigations into the binding mechanism utilized steered MD simulations at various pulling speeds and umbrella sampling to estimate the energy profile of HVEM-based inhibitors with LIGHT. These comprehensive analyses revealed key interactions and different binding mechanisms, highlighting the increased binding affinity of selected peptide variants. Experimental circular dichroism techniques confirmed the structural properties of these variants. This study not only advances our understanding of the molecular basis of HVEM-LIGHT interactions but also provides a foundation for developing novel therapeutic strategies for immune-related disorders. Furthermore, it sets a gold standard for peptide inhibitor design in drug development due to its systematic approach.

## 1 Introduction

The herpesvirus entry mediator (HVEM) and its ligand LIGHT are members of the tumor necrosis factor superfamily (TNFSF).^1,2^ These proteins play multiple roles in the immune system by regulating diverse processes and T-cell responses. In particular, HVEM and LIGHT function as costimulatory molecules, and their interaction results in the enhancement of T-cell growth, differentiation, and cytokine secretion.^3,4^

**Figure 1:**
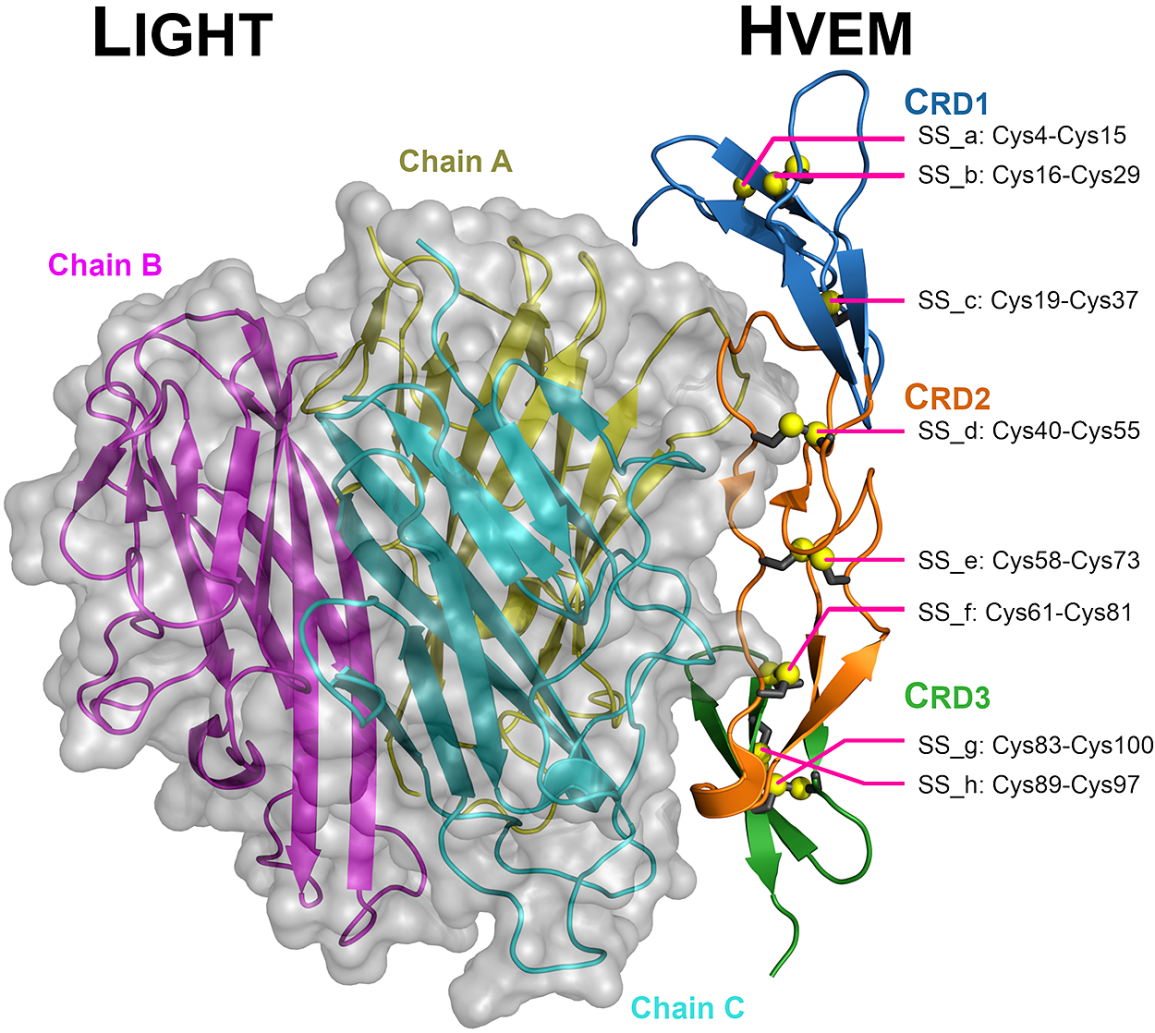
Cartoon representation of the LIGHT-HVEM complex with the semitransparent surface representation of the LIGHT trimer (PDB code: 4RSU). CRD's1–3 are indicated as blue, orange, and green colors, respectively, while chains A–C from the LIGHT trimer are colored yellow, magenta, and cyan, respectively. HVEM disulfide bonds are shown as gray sticks and yellow spheres (a–h).

HVEM is a type I transmembrane glycoprotein composed of 283 amino-acid residues, divided into a signaling peptide (−37–0) and extracellular (1–164), transmembrane (165–185), and cytoplasmic (186–245) parts. The extracellular fragment of HVEM contains four cysteine-rich domains (CRD1:1–38, CRD2:39–81, CRD3:82–124, and CRD4:125–164), and the disulfide bonds within these domains are critical for establishing the tertiary structure of this receptor.^5^ HVEM provides a stimulatory signal by interacting with the LIGHT and LTα ligands via the CRD2 and CRD3 domains^2,6^ (Figure 1), and an inhibitory signal when it binds to the BTLA^7^ or CD160^8^ receptors via the CRD1 domain. On the other hand, neither the CRD4 structure nor its affinity to other molecules is known.^9^ This binding pattern of HVEM offers multiple targetable molecules for modulating immunological responses.

LIGHT, also known as TNFSF14, is a homotrimeric type II transmembrane protein expressed on activated T-cells and immature dendritic cells.^2,4^ It is involved in various biological processes, including inflammation, immune regulation, and apoptosis, and plays a critical role in the pathogenesis of various autoimmune diseases and cancer,^10^ as well as COVID-19-induced pneumonia and inflammation.^11^ Therefore, understanding the molecular mechanisms of LIGHT signaling is crucial for developing new therapies for these diseases.

The HVEM-LIGHT complex could be involved in various inflammatory processes, such as inflammatory bowel disease^12^ and rheumatoid arthritis (RA),^13,14^ due to the modulation of T-cell proliferation. Furthermore, studies suggest that the interaction between these proteins may play a major role in transplantation. For example, targeting LIGHT protein using HVEM-Ig and LTβR-Ig fusion proteins significantly reduces allogeneic T-cell immune responses, including proliferation and cytotoxic T-cell activity.^4^ Similarly, the use of HVEM mAbs has shown a similar effect.^15^ All these data highlight that the HVEM-LIGHT complex could be a key factor in controlling T-cell immune responses and may be a candidate to target in different immune-mediated diseases.

As short peptides usually have lower toxicity, drug–drug interaction, and production cost,^16^ the aim of the present study is to systematically design short peptide inhibitors of the HVEM-LIGHT interaction based on HVEM-binding fragments. Another reason why we conduct research on peptides is the fact that they are easily degraded in the body.^17^ This fact, at first glance negative, in therapeutic applications may have a positive effect. Application of peptides may avoid severe side effects present in immunotherapy using antibodies,^18^ which are related to their long half-life in the body. To achieve this, we designed peptides that are fragments of the HVEM molecule, which can potentially block the formation of the HVEM-LIGHT complex and determined their affinity to the trimeric LIGHT protein. In our work, we used standard L-amino acids. Due to the fact that our work is based on structures occurring in nature, the use of other compounds, e.g., D-amino acids, could significantly affect the final structure of the designed compound, which would affect their affinity to the LIGHT protein. At the same time, since the pharmaceutical and laboratory costs of introducing disulfide bonds in proteins are high, we strive to minimize their presence to only those disulfide bonds which are essential. The presence and importance of the disulfide bonds in the HVEM molecule, used as a template for the design of the ligands, is one of the reasons why typical machine learning tools, such as AlphaFold 3,^19^ cannot be effectively used to examine various disulfide-bond variants.

A common, yet effective, computational approach for estimating binding affinities involves docking the ligand molecule to the receptor, followed by the estimation of binding free energy using methods such as molecular mechanics with generalized Born or Poisson–Boltzmann surface area solvation (MM-GBSA, MM-PBSA) on the MD trajectories of the obtained complex models.^20^ In this work, we performed detailed analyses of binding free energies, dynamics, and stability of the obtained complexes based on extensive molecular dynamics (MD) simulations in a state-of-the-art force field and water model. However, this approach assumes rigid docking, where the conformation of the ligand does not change upon binding.^21^ This assumption is unlikely to hold in the case of HVEM-based fragments, especially when not all disulfide bonds are present. Therefore, we studied the influence of disulfide bonds on HVEM domain stability. We also determined amino-acid residues and disulfide bonds that have a crucial influence on the formation and stability of the HVEM-LIGHT complex. Moreover, we performed a comparison between trajectories of free and bound HVEM variants, incorporating this information into the binding free energy estimations. To further elucidate the binding mechanism of the designed peptides to LIGHT, we conducted steered MD (SMD) simulations to pull the most promising HVEM-based inhibitors from the LIGHT trimer at various pulling speeds and then calculated the potential of mean force (PMF) using the umbrella sampling method. This allowed us to obtain a single-residue mutant of the CRD2 domain (CRD2e_K54E) with a single disulfide bond (C58–C73) that binds most strongly to LIGHT out of all the examined variants based on whole domains. Additionally, we identified a truncated variant of the CRD2 domain (CRD2(39–73)e) with the same disulfide bond present, which is characterized by similar binding strength despite being shorter in length; however, it exhibits a slightly different binding mechanism.

## 2 Methods

### 2.1 Binding Free Energy Estimation Approaches

One of the most important properties taken into account during drug design is the binding free energy of the complex formation, which can be presented as
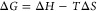
1where Δ*G* is the binding free energy, Δ*H* is the enthalpy of binding, *T* is temperature, and Δ*S* is the entropy change of the system upon complex formation.

**Figure 2:**
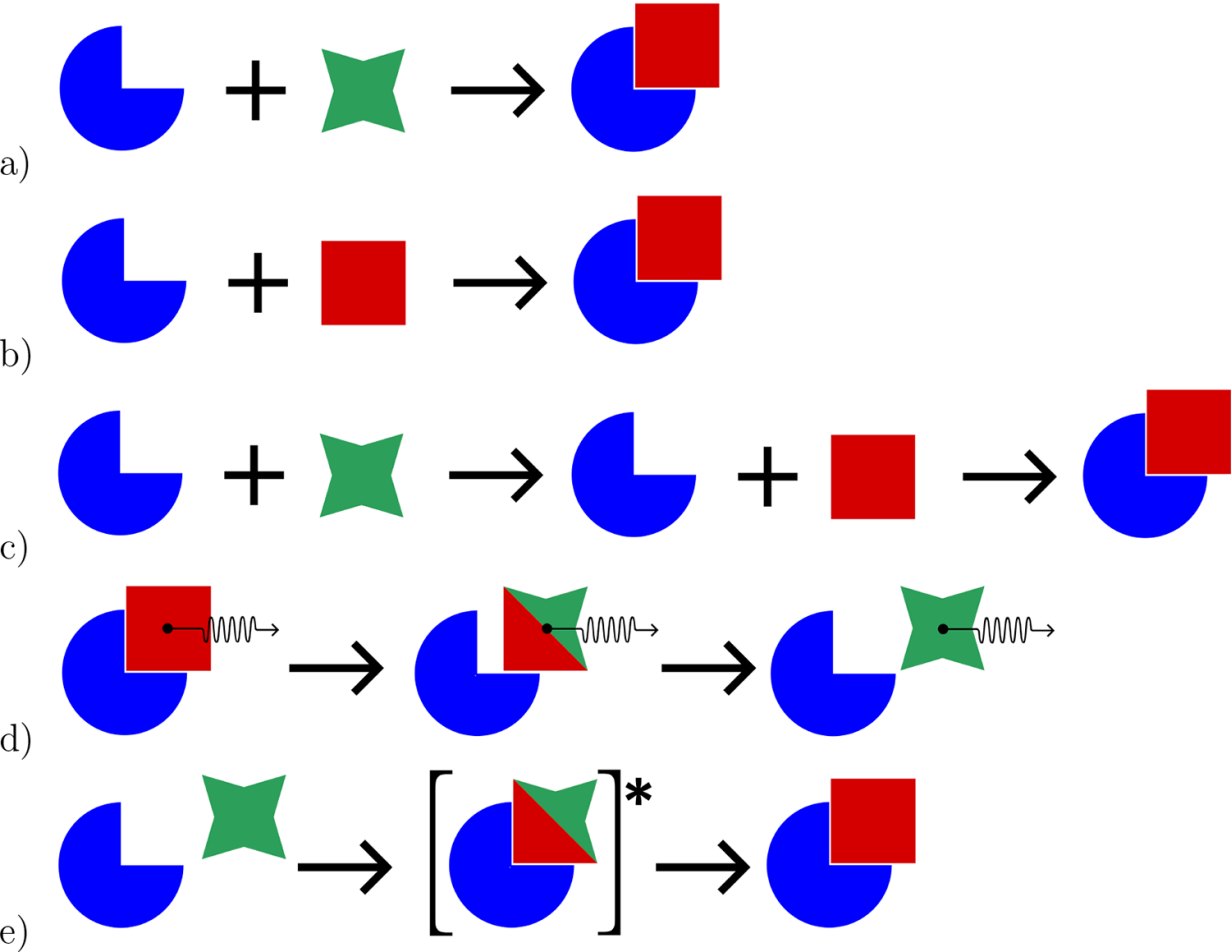
Diagram presents a simplified representation of receptor (blue; in this study, the LIGHT trimer) and ligand (red and green, with colors indicating bound and free conformational states, respectively; in this study, HVEM-based inhibitors) binding-unbinding pathways and mechanisms: (a) physiological, (b) MM-GBSA/MM-PBSA approach, (c) MM-GBSA/MM-PBSA approach with an additional calculation of the internal work connected to the transition between free and bound states of the ligand, (d) SMD approach to pull the ligand from the receptor at various pulling speeds, allowing for various levels of the bound-free state transition of the ligand due to the nonequilibrium character of the SMD simulations and limited time for conformational changes depending on the pulling speed, and (e) PMF calculation based on a series of US trajectories capturing the transition between bound and free states of the ligand depending on a distance between molecules.

It is directly related to the binding affinity/equilibrium dissociation constant *K*_D_ = exp(Δ*G*_bind_/*RT*). When designing peptides, one should take into consideration their flexibility and conformational changes upon binding (Figure 2A), as peptides bind either by an induced fit mechanism (where the ligand changes conformation upon binding)^22^ or by a conformational selection mechanism (where only a few conformations appropriate for binding are selected from the conformation space available to the ligand).^23^

In theoretical approaches, the most commonly used method to determine Δ*G* is MM-GBSA^24^ (Figure 2B). In this approach, a conventional MD simulation in explicit (more often) or implicit (less often) water is performed, followed by the selection of a certain number of frames from a given trajectory and computation of the binding energy as given by the eq 2:^25^
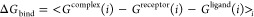
2where <···>_*i*_ denotes an average over *i* snapshots extracted from the MD trajectory. Δ*G* represents the free energy of a given system. It should be noted that this is not literally the free energy, as this term contains only solvent entropy when simulations are run with the explicit solvent model. The entropy contribution in the MM-GBSA method could be further extended to the difference between bound and unbound systems in a given conformation. This is usually obtained via normal-mode analysis or quasi-harmonic approximation.^25^ In this manner, one should, in theory, obtain binding free energies that are closer to experimental ones. However, this method is computationally expensive and may even lead to a worsening of the correlation between theoretically and experimentally obtained binding free energies.^26^

MM-GBSA and MM-PBSA methods, despite being extremely fast and commonly used, have several important downsides:^21^ (a) It considers the same conformation of the unbound and bound states, similar to rigid docking. (b) Entropy is only incorporated as solvent entropy, making it a very rough approximation of the whole system entropy. (c) Entropy can be obtained via normal-mode analysis or quasi-harmonic approximation. However, this method is computationally expensive, often requiring hours to days of calculation for a single snapshot depending on the system size, and may only provide a rough approximation of the entropic contribution. (d) It ignores solvent/counterion-mediated interactions.

MM-GBSA calculation could be, in principle, improved by simulating the unbound ligand (Figure 2C). In this way, the internal work associated with the conformational changes can be computed with the use of eq:
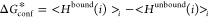
3where ΔG*_conf_ is the free energy associated with conformational changes with the restriction that it contains only solvent entropic contributions. This energy describes the difference between the average internal (conformational) energy of a ligand in a bound ensemble state and the average internal energy of the ensemble in an unbound state.

In this way, one can take into consideration conformational changes upon binding; however, the peptide conformational enthalpy difference might be burdened with a large error as (i) it comes from separate simulations, (ii) may be sensitive to ligand conformation changes, especially in the case of poorly structured molecules, like most peptides, and therefore should be handled with extreme care. Moreover, this method does not overcome the solvent/counterions-mediated interaction problem.

Another technique that allows the computation of free energy is SMD. In this method, the ligand is pulled away from the receptor (Figure 2D), and the free energy can be computed from Jarzyski equality:^27^ exp(−Δ*G*/*RT*) = <exp(−*W*/*RT*)> , where *W* is the work of a system. This method takes into consideration entropy in a direct manner and conformational changes during unbinding, as well as solvent effects; however, it is prone to other issues:

(a) requires a large number of trajectories and many starting points coming from equilibrium states and therefore may have problems with convergence, (b) for practical reasons, the Jarzynski equality is simplified to infinitely slow pulling limit (Δ*G* ∼ *W*), however, as the work depends on the velocity of pulling and is always greater than the free energy of binding (Δ*G* ≤ *W*).

In the case of many peptides, only one binding mode is dominant, and therefore, in many studies, a single starting point is used; therefore, this starting point should be handled with extreme care.

Finally, binding free energy can be computed from the PMF using eq 4:
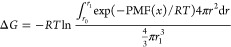
4where *x* is the coordinate in which PMF is derived, *r*_0_ and *r*_1_ are the boundaries determined for the bound state, *R* is the gas constant, and *T* is the simulation temperature. This technique has to be performed based on the data from enhanced sampling algorithms (Figure 2E), such as replica exchange MD,^28^ but even then it still can lead to nonuniform sampling of the energy landscape. Therefore, the safest way is to perform umbrella sampling MD,^29^ which can be computationally demanding as it requires running multiple separate trajectories, from which all have to reach equilibration. Another downside of this method is the choice of the coordinate of the derivation of PMF (*x*), as the PMF may depend on this choice.^30^ In some cases, one can face with convergence problem as the umbrella sampling depends on the force constant and bin size.

There are also other techniques that allow estimating binding free energies.^31−33^ Overall, there is no perfect technique to determine the binding free energy, and each technique has its pros and cons. Therefore, in this work, we performed a rigorous analysis using all techniques showing their differences and similarities when applied to peptide drug design.

### 2.2 Simulated Systems

**Figure 3:**
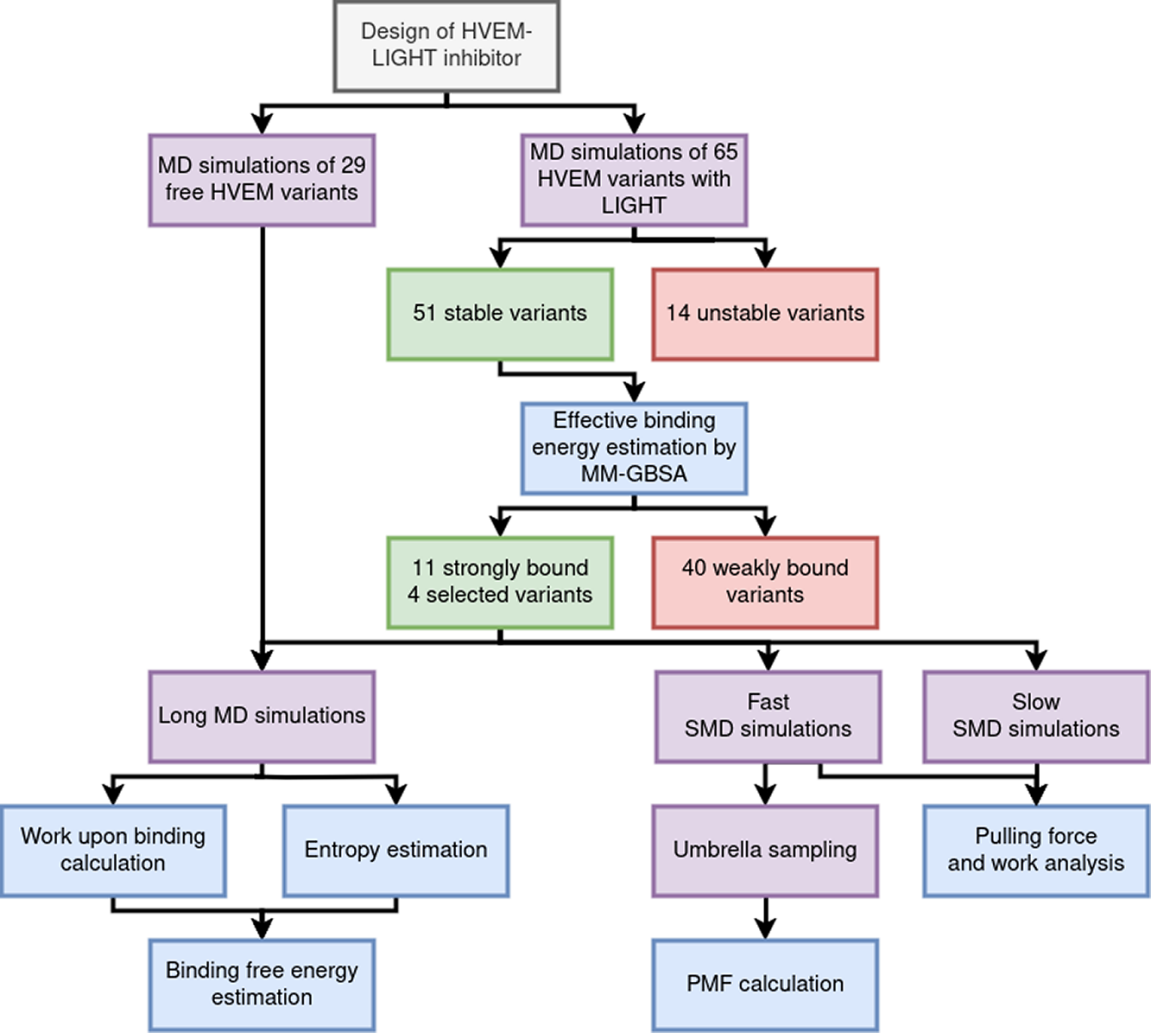
Flowchart illustrates the framework for designing highly effective HVEM-LIGHT inhibitors based on the HVEM molecule (gray box) using MD, SMD, and US simulations (purple boxes), along with their analyses (blue boxes), while arrows denote the general order of operations. The number of contacts between inhibitors and LIGHT during MD simulations was used as exclusion criteria for weakly bound inhibitors to reduce the computational costs of further studies.

Our studies involved MD simulations of 65 molecules (Figure 3), based on the HVEM protein, including various lengths, disulfide-bond combinations, and amino-acid substitutions interacting with the LIGHT trimer (Table S1), while selected 29 variants were simulated also alone, in bulk water, without the presence of LIGHT protein, for sake of the comparison (Table S2). To simplify the naming, each disulfide bond was assigned a letter from a to h (Figure 1), based on the position of the first cysteine residue involved in a bond (detailed explanation is provided in Table S1).

### 2.3 All-Atom MD Simulations

The first set of MD simulations was used to determine the stability of the HVEM protein and its variants in an aquatic environment, while the second set was used to determine the binding affinity of proposed inhibitors to LIGHT and the change of structural properties upon binding. All MD simulations adapted the experimental model of HVEM-LIGHT (PDB: 4RSU,^9^) as the initial conformation, with or without disulfide-bond modifications, amino-acid substitutions, and truncations (Table S1).

Subsequently, we focused primarily on the amino-acid residues that exhibited the most repulsive or least attractive tendency toward the LIGHT trimer, as testing all possible combinations was infeasible. The mutations were selected based on chemical differences, change of charge, hydrophobicity/hydrophilicity nature, and large/small side-chain. If a single mutant demonstrated improved binding affinities compared to the wild-type molecule, we attempted to replace other residues showing the most repulsive or least attractive interactions. For every simulated variant, we modified it by manually removing the side-chain of the original residue and changing the backbone residue name to a given mutation in the text editor. Then, the side-chain was reconstructed during the preparation of the coordinates and topology files in tLeap. Minimization, equilibration, and production runs were then performed.

All systems were prepared using the tleap program, part of AmberTools23,^34^ with the ff19SB force field^35^ and four-point OPC water model,^36^ while the simulations were run in pmemd.cuda, part of Amber22.^37^ The HVEM/LIGHT complex and its equivalents were surrounded by a minimum 17.5 (20 for CRD1) Å layer of water in the shape of a truncated octahedron. To avoid the introduction of additional entropy from the ions, the system’s charge was only neutralized, and no constant salt concentration was used with the use of Na^+^ or Cl^–^ counterions. Each system underwent 2 minimization procedures: first with 1000 minimization steps (400 of steepest descent followed by 600 steps of conjugate gradient) and restraints placed on all solute atoms with constant *k* = 100 kcal/mol/Å^2^, and second with 2000 minimization steps (800 of steepest descent followed by 1200 steps of conjugate gradient) and restraints placed on all heavy atoms with constant *k* = 100 kcal/mol/Å^2^. Subsequently, each system underwent 3 equilibration cycles: first to set up the proper temperature of the system (heating up from 10 to 300 K over 10,000 MD steps in the NVT ensemble, each of 1 fs with restraints placed on heavy atoms), second to reach a proper density (100,000 MD steps in the NPT ensemble, each of 1 fs with restraints placed on heavy atoms), and the third to equilibrate the system (400,000 MD steps in the NPT ensemble, each of 2 fs with restraints placed on Cα and Cβ atoms). Subsequently, three independent MD trajectories were carried out for 100 × 10^6^ steps, each of 2 fs, providing 600 ns in total for each system. Four systems (CRD2e, CRD2def, CRD2e_K54E, and CRD2(39–73)e) were simulated for a longer time −500 × 10^6^ steps, each of 2 fs, providing a total of 3000 ns per system. All production simulations were carried out in the NVT ensemble and with no restraints, with the Langevin thermostat set to 300 K. In all explicit solvent simulations, the Particle Mesh Ewald method with a cutoff for nonbonded interactions set at 9 Å was used to speed up calculations.

In addition to classical MD simulations, a series of SMD simulations (25 trajectories per system, each of at least 80 ns) was run to establish a binding-unbinding mechanism for CRD2e, CRD2def, CRD2e_K54E, and CRD2(39–73)e (Figure 5) representative models. Each of them started from the representative model of the above-mentioned HVEM variants obtained by the clustering of the last parts of the trajectories. To ensure that no interperiodic contacts were formed during the simulation, a larger number of water molecules was added to form a layer of 30 Å, giving a truncated octahedron box dimensions of approximately 122 × 122 × 122 Å. Then, a standard minimization and equilibration procedure was performed as for the regular MD simulations, followed by a 10 ns run to make sure that the water molecules oriented properly around the complex. For those systems, 25 independent SMD trajectories were run with restraints placed on the centers of mass of LIGHT and CRD2 molecules with a force constant of 10 kcal/mol and a pulling speed of 0.05 m/s to achieve full dissociation of the system (minimal distance of any heavy atoms from the proteins above 6 Å), which translates to extensions above 40 Å.

To investigate the influence of pulling speed on the observed force and work response, an additional SMD run was performed with a pulling speed five times slower at 0.01 m/s. However, to expedite calculations, a smaller water box of 20 Å was used, and simulations were conducted only until most, but not all, trajectories were dissociated (to 25 Å of extension). This adjustment should not significantly impact the results but allows for some computational time savings. Given the slower pulling speed, five times more data points were collected for averaging. Consequently, we opted to run only 10 independent trajectories, each lasting 250 ns (totaling 2500 ns per system), as opposed to 25 trajectories, each lasting approximately 80 ns (totaling 2000 ns per system), for the faster pulling.

After the SMD simulations, PMFs were computed using the distance between the centers of mass of the proteins as a PMF variable. To set up umbrella sampling simulations, the strongest interacting conformation from SMD at a given distance was used as a starting structure for the umbrella sampling simulation. For each system, at least 44 trajectories were run (with the distance of centers of mass from −5 to +37 Å with 1 Å interval compared to the distance from the initial position from classical MD simulations), each of 100 ns with a spring constant *k* = 5 kcal/mol/Å^2^. Distances were collected every 1000 steps, and the last 95 ns of the trajectories (47,500 snapshots) were used for the weighted histogram analysis method (WHAM)^38^ to calculate the PMF. From PMFs, association constants were computed (eq 4).

### 2.4 Analysis

Binding free energy Δ*G* and effective free energy analyses were performed on the converged parts of the trajectories (three for each of the systems): the last 20 out of 200 ns (last 200 out of 2000 snapshots) and the last 500 out of 1000 ns (5000 out of 10,000 snapshots) for simulations of HVEM variants with and without LIGHT and averaged out over three independent trajectories. The free energy change between the bounded and free states of the receptor–ligand complexes was estimated using the MM-GBSA method^39^ with the recommended GB-Neck2 estimation method with the corresponding radii values.^40^ In our research, we used it as a method of MD analysis because it allows us to obtain a satisfactory estimate of the receptor–ligand interaction energy at a relatively low time and computational cost. The use of other methods (such as free energy perturbation, FEP), given the number of systems analyzed in our work, could significantly increase the computational cost of the research without increasing the accuracy of calculations that would have an impact on further research.^41^ For most of the systems, the effective free energy^42^ was analyzed, which should provide good qualitative results for similar compounds.^43^ Energy decomposition was performed on a per-residue and pairwise per-residue basis, with the latter summarized for each residue in the HVEM variant of its interactions only with LIGHT residues, to show the difference in how each residue influences overall binding stability and the influence on the interaction partner. The entropy contribution was calculated using the normal-mode analysis method for 200 ns of designed systems with the highest binding affinity and 1000 ns simulations of CRD2e, CRD2def, CRD2e_K54E, and CRD2(39–73)e systems. In the first case, calculations were performed for 10 frames extracted evenly from the last 20 ns of 200 ns simulations, while for the longer simulations, 20 frames from the last 200 ns were used for entropy calculation due to the large computational cost with the maximum number of iterations to calculate entropy set up to 1000. Such a combination of force field, water model, and MM-GBSA method is the modern standard for the binding affinity prediction.^44^ To even further refine this approach, internal work associated with the change between bound and unbound compounds was calculated by an additional run of the MM-GBSA analysis for selected unbound HVEM variants (in bulk water) to obtain their enthalpy values and compared them with the one from bound molecules. The correlation between the values determined on the basis of MD, SMD, and PMF was confirmed on the basis of Pearson and Spearman correlation coefficients.

Analyses of: Cα root-mean-square deviation (RMSD); root-mean-square fluctuations (RMSF); radius of gyration (*Rg*); maximum distance of any heavy atom to center of mass of the protein (*Rg*_max_); end-to-end distance (e2e); solvent accessible surface area (SASA) (using the LCPO algorithm^45^); contacts based on distance cutoffs of 8 and 6 Å for determining dissociation of the complex in MD and SMD, respectively, (contact is defined as 0 if there are no heavy atoms from the complex partner within the range, and as 1 if there is at least one such heavy atom). During our work, we wanted to obtain highly effective inhibitors, for which it is important to maintain a structure that allows the formation of a stable complex with the receptor. Values aformentioned above can be used to determine the stability of compounds in a relatively simple way during the simulations and to visualize any changes in the conformation of simulated compounds that occur during the studies. The fraction of secondary structure was analyzed with the DSSP algorithm^46^ implemented in AmberTools2023 cpptraj.^34^ For comparison, the fraction of secondary structure was analyzed with STRIDE^47^ and KAKSI.^48^ LRMSD, calculated only for the backbone of the ligand after superposition on the receptor, was determined using DockQ.^49^ As STRIDE, KAKSI, and DockQ can process only a single structure at the time, an in-house script was used to process equilibrated parts of trajectories. Representative models were generated by hierarchical clustering of the converged parts of the simulation. All figures were prepared with the use of Python 3.7 and gnuplot5.2, while structures were visualized with PyMOL 2.5.0.^50^

### 2.5 Selectivity Check

To check if the designed peptides characterized by the strongest effective binding energy are binding selectively to the LIGHT binding groove, docking studies with use of HDOCK^51^ were performed. We also checked if the molecules could effectively bind to other proteins, which in physiological conditions bind the HVEM protein, such as BTLA.

Additionally, the UNRES-dock^52^ procedure was performed as in our previous paper.^20^ The dominant structure of the peptide from the all-atom simulation was used as the starting structure. The peptide was randomly oriented with respect to LIGHT, and weak restraints were imposed on each chain. Note that no peptide-LIGHT restraints were imposed. Afterward, multiplexed-replica exchange MD^53^ with the NEWCT-9P force field was applied,^54^ with temperatures ranging from 250 to 400 K and 10,000,000 steps performed. After the simulation, the bin-less weighted histogram analysis method^55^ along with clustering^56^ at 280 K was used to obtain 10 clusters for each of the systems.

### 2.6 Peptide Synthesis

Selected peptides were synthesized by the solid phase peptide synthesis method on LibertyBlue synthesizer using the Fmoc/tBu strategy. The resin used was Rink Amide Pro Tide (LL) with a capacity of 0.18 mmol/g. In order to selectively create disulfide bridges, two cysteine derivatives were used: Fmoc-Cys(Acm)–OH and Fmoc-Cys(Trt)–OH. Cysteine residues present in the amino-acid sequence of HVEM and not involved in the disulfide bond were replaced with α-aminobutyric acid (Abu), while methionine residues were replaced with norleucine (Nle) in order to avoid the oxidation of sulfur in side-chain. The reaction was carried out at room temperature for 24 h. Then the peptides were cleaved from the resin using a mixture consisting of 88% TFA, 5% phenol, 5% deionized water, and 2% triisopropylsilane. 10 mL of the mixture was used per 1 g of resin, and all were stirred for 4 h at room temperature. Then resin was filtered off, and Et_2_O was added to the remaining mixture to precipitate the peptide. The mixture was centrifuged three times at 4000 rpm for 15 min at 4 °C. Then the peptide was dissolved in deionized water and freeze-dried.

### 2.7 Peptide Purification

The purification of the peptides was carried out on a reverse-phase high-performance liquid chromatography (RP-HPLC) on a Luna 5 μm C8(2) 100 Å column. A 10-fold excess of DTT relative to free sulfhydryl groups was added to the aqueous solution of the purified peptide. The mixture was subjected to ultrasound at a temperature of 40 °C before being applied to the column. Two solutions were used during the purification: (A) deionized water with 0.1% TFA (v/v), and (B) 80% solution of acetonitrile in water with 0.08% TFA (v/v). A linear concentration gradient from 5 to 50% B in A was applied over 120 min. The purification process was monitored using a UV detector measuring the absorption at 222 and 254 nm. Peptide purity was verified using RP-UHPLC with PDA and ELSD-LT detectors (SHIMADZU, Kyoto, Japan) Kromasil C8 analytical column Kinetex C8 (100 × 2.1 mm; 2.6 μm; 100 Å) with using a 5–100% gradient of solution B in 15 min.

### 2.8 Disulfide Bond Formation

The first disulfide bond was formed between the cysteine residues with the sulfhydryl group protected by a trityl group, which was removed when the peptide was pulled from the resin. After purification, the peptide was dissolved in a mixture of water and methanol (1:9, v/v) at a concentration of 40 mg/L. The pH of the mixture was adjusted to between 8 and 9 using ammonia–water. The mixture was stirred for 7 days, and compressed air ran through the solution. The progress of the reaction was monitored by RP-HPLC. After this time, the methanol was evaporated, and the remaining aqueous solution of the peptide was freeze-dried. The peptides with one disulfide bond were purified according to the procedure given earlier, while the peptides with two disulfides were subjected to the second oxidation. The second disulfide bond was created according to the following procedure: The peptide was dissolved in a mixture of acetic acid, water, and methanol (1:1:9, v/v/v) at a concentration of 40 mg/L. A 25–50 fold excess of iodine dissolved in methanol was then added to the mixture. The mixed solution was left for a week. After this time, the mixture was filtered through a Dowex ion exchange bed to remove excess iodine. The solvent was evaporated, and the obtained peptide was dissolved in water and lyophilized. Then, the peptide was purified according to the procedure given earlier.

Circular dichroism (CD) spectra were recorded at the Circular Dichroism Laboratory, Faculty of Chemistry, University of Gdańsk. The analysis was carried out using a CD J-815 circular dichroism spectrometer by Jasco. Peptide solutions with concentrations of 0.15 mg/mL were used for the tests, and all CD spectra were taken in water at 298 K as recommended.^57^ The results are presented in the form of the dependence of the molar ellipticity on the wavelength.

## 3 Results

### 3.1 Structural Properties of Unbound HVEM Protein and Its Disulfide-Bond Variants

For all of the structural fragments in which cysteine residues forming disulfide bonds are present, reduction of those bonds causes spatial separation of these elements (Figure S1). When the full HVEM structure is present, reduced cysteines tend to maintain native contacts between them, particularly noticeable in the case of the second domain and one disulfide bridge in the first domain. However, this effect is not observed in the specific HVEM domains when all cysteine residues are reduced (Figure S1). The introduction of any single disulfide bond consistently enhances structural stability. However, the strengthening of this effect does not always occur with the addition of another disulfide bond. Furthermore, an interdomain stabilizing effect can be observed. This phenomenon is particularly pronounced in the case of CRD2, which exhibits remarkable stability even in the absence of any disulfide bonds when surrounded by CRD1 and CRD3.

In CRD2, which is the most important domain for binding LIGHT, the disulfide bond between Cys58-Cys73 (e) is the only one that significantly increases structural stability when only one disulfide bond is present on its own (reducing RMSD from 10.52 ± 0.28 to 4.93 ± 0.65 Å; Figure S2) and can be compared to the stability of the two-disulfide-bond variants CRD2df and CRD2ef. When the (e) disulfide bond is combined with Cys40-Cys55 (d), CRD2 domain stability slightly increases (CRDde: 3.84 ± 0.79 Å), approaching the case when all three bonds are present (CRD2def: 2.60 ± 0.18 Å).

The radius of gyration (RG) and RGmax analyses show that the compactness of CRD2 is not significantly affected by the presence or absence of disulfide bonds, except for the CRD2no and CRD2d (Figure S2) cases, which exhibited a more relaxed or loose structure.

The details of the influence of all disulfide bonds on CRD stability (Figure S1) are described in the Supporting Information section Influence of disulfide bonds on CRD stability.

The content of both α-helices and β-sheets in HVEM domains is heavily impacted by the presence (or absence) of given disulfide bonds and the presence (or absence) of the interdomain interactions (Table S3). While the full HVEM sequence has a strong tendency to form β-sheets over α-helices, which is understandable, as this secondary structure element requires distant parts of the protein to obtain complete stabilization, domains usually prefer more disordered and α-helical structures. The largest differences are observed for variants with all and none of the disulfide bonds present; however, a truncation of the CRD2 further modifies its behavior. Despite that, the predominant type of secondary structure in all of the HVEM variants is always disordered (coil) and turn, which is closely followed by β-sheets, while α-helix content is always low (<8%). The addition of disulfide bonds has varying degrees of impact but predominantly draws the secondary structure content close to the all-disulfide-bond variants and the complete HVEM chain with all disulfide bonds present.

The CD spectra generated with a Web server (PDB2CD)^58^ reveal some helical (Figure S3B) content which is not present in the analysis with other methods and is not observed in the experimental CD spectra (Figure S3A). Despite the fact that CD is a very low-resolution method of secondary structure determination, especially for flexible structures,^59^ for all peptides, the coil structure is the dominant one as the value of molar ellipticity is below 0 for 200 nm, which is in agreement with all secondary structure determination tools used for these simulations. Therefore, it can be concluded that peptides do not form significant amounts of stable secondary structures and are mainly unstructured.

### 3.2 Changes of the Structural Properties of HVEM and Its Variants Upon Binding LIGHT Trimer

HVEM variant structures become more rigid upon LIGHT binding, which is particularly evident in variants without any disulfide bonds present (Figure S2). This effect is even more pronounced when the complex exhibits high binding affinity. However, upon binding, the structure of CRD2 variants becomes slightly more expanded. This is evident in the relatively small increase in the RG and a larger increase in RGmax, with the only exception being CRD2no. In the case of CRD2no, the combination of high flexibility, change in SASA, and high binding affinity makes it more strongly impacted by the binding of LIGHT.

Interestingly, the theoretical SASA of HVEM variants (except for CRD2no), calculated without the presence of any other molecules, remains similar for both free and bound HVEM variants with only a slight increase (Figure S4 and Table S4). In most of the cases, the difference in SASA upon binding arising from hydrophobic residue is relatively small. This observation suggests that hydrophobic interactions are not the primary contributors, in most cases, to HVEM-LIGHT interactions.

In general, the binding of HVEMall to LIGHT does not have a significant impact on the fluctuations (RMSF; Figure S5) and secondary structure of the molecule (Figure S6). Moreover, it is noteworthy that the most structurally stable region upon binding to LIGHT is CRD2. The most stable HVEM variant overall is CRD2all, whose secondary structure remains unchanged if all disulfide bonds are present, similarly to the complete HVEM chain with all disulfide bonds present. The most drastic changes in the secondary structure upon LIGHT binding are observed for CRD3no, which shifts strongly into β-sheets.

### 3.3 Effective Binding Energy to Select the Best Candidates for LIGHT Inhibition

**Figure 4:**
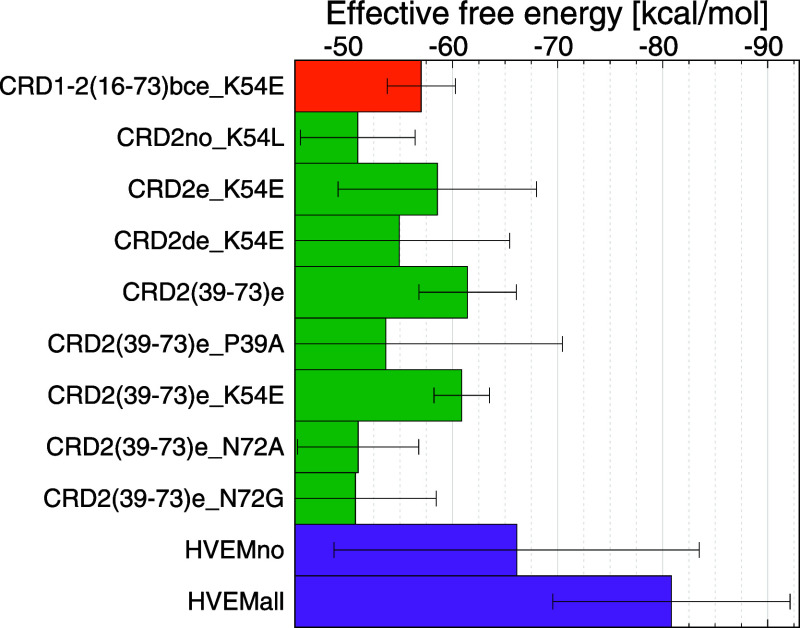
Bar plot of the effective binding free energy for HVEM variants with LIGHT with the highest binding affinity (< −50 kcal/mol), calculated as the average over three independent trajectories, with error bars representing standard deviations.

The effective binding energy of HVEM and its variants to the LIGHT trimer was determined using the MM-GBSA method, while the contribution of particular amino-acid residues to the total effective binding energy was calculated using per-residue and pairwise energy decomposition, however, only for the complexes which did not dissociate during MD simulations (Figure S7). Our analysis revealed that, among the three HVEM domains, CRD2 emerged as the primary and the most robust interacting entity with the LIGHT trimer (Figures 4, S8–9); therefore, our further efforts to design the strongest binding peptide were focused mostly on this HVEM fragment. It should be noted that disulfide bonds have tremendous influence on the binding affinities, i.e., CRD2no (CRD2 domain without disulfide bonds) demonstrated significantly higher affinity to LIGHT compared to CRD2all (with all disulfide bonds).

In the next step, we established that Lys54 has the most unfavorable energetic effect in most of the CRD2 variants; therefore, it was our first candidate for point mutations in order to attempt to increase binding affinity to the LIGHT trimer. Serine, leucine, isoleucine, glutamic acid, aspartic acid, tyrosine, and valine were selected as candidates for replacements to scan amino-acid residues of the opposite charge and properties to lysine. We selected CRD2 variants with different disulfide bond patterns which exhibited the best affinity: CRD2no and CRD2de, and the worst, but most stable in solution, one: CRD2e.

Unfortunately, no point mutations in CRD2no significantly improved the binding energy (Figure S8), while the mutation to serine dramatically reduced the binding affinity (−21.46 ± 3.90 kcal/mol). In the case of CRD2de, only the mutation to glutamic acid improved the effective binding energy (−54.92 ± 10.52 kcal/mol). Surprisingly, the highest effective binding energy was observed for the same substitution, but in the CRD2e variant, which is characterized by the lowest binding affinity among all CRD2 variants, but with the single-point mutation, it becomes the variant with the highest affinity (CRD2e_K54E: −58.55 ± 9.45 kcal/mol). Smaller improvement was observed for CRD2e with a substitution of Lys54 to valine (−41.17 ± 7.20 kcal/mol).

We also designed a series of double-point mutants to mitigate the unfavorable energy effect from Asp62, which was emphasized in the designed mutants. This residue was replaced with alanine, leucine, lysine, and serine. Unfortunately, none of the designed double mutants exhibited a more favorable effective binding energy than the original variant (Figure S8).

**Figure 5:**
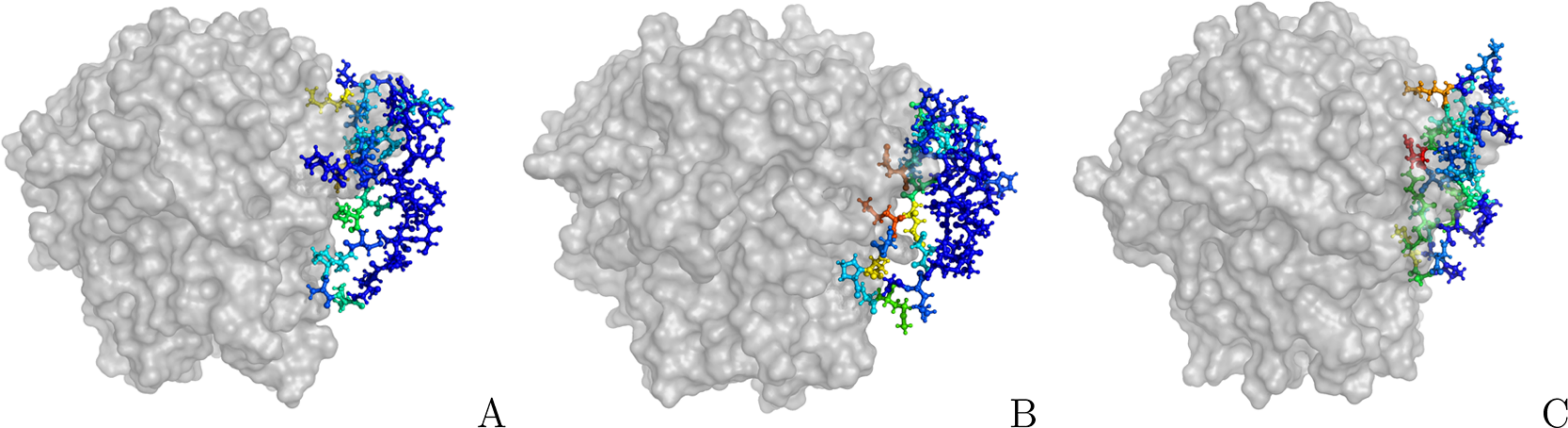
Representative models of selected HVEM-based variants, obtained by clustering three independent trajectories, colored by the pairwise effective binding energy (rainbow colors from blue to red, where blue indicates the lowest binding energy and red is the highest), in ball-and-stick representation for: (A) CRD2e, (B) CRD2e_K54E, and (C) CRD2(39–73)e, interacting with the LIGHT trimer (gray surface).

Based on the binding energy decomposition (Figure S9), we determined that not all amino-acid residues are involved in the formation of a complex with the LIGHT trimer. Therefore, we designed four peptides accordingly: two based on the CRD1 domain, namely CRD1(16–38)bc and CRD1(16–38)no, and two on the CRD2 domain, CRD2(39–73)e and CRD2(39–73)no, which included only amino-acid residues with sufficient affinity for the LIGHT trimer. It should be noted that, in general, peptides based on the CRD1 domain have much lower affinity for LIGHT than those based on the CRD2 domain, and the truncated variant showed even lower affinity (−18.89 ± 6.41 and −44.35 ± 6.06 kcal/mol, respectively). However, truncation of the CRD2 amino-acid residues resulted in a significant increase in effective binding energy, with the CRD2(39–73)e peptide being the strongest binding peptide (−61.43 ± 4.63 kcal/mol), despite its shorter polypeptide chain. Conversely, CRD2(39–73)no exhibited lower affinity than CRD2no. Remarkably, the removal of the 8 C-terminal residues from CRD2e resulted in a CRD2(39–73)e conformation that barely extends beyond the binding site of the LIGHT trimer (Figure 5).

In an effort to further improve the affinity of CRD2(39–73)e, we designed a series of mutants in which the Pro39, Lys54, Cys61, and Asn72 residues were substituted by other amino-acid residues. Unfortunately, these mutants displayed lower affinity for the LIGHT trimer than the parent peptide. To check the shortened configuration with all natively present disulfide bonds in this fragment (d and de disulfide bonds), we designed CRD2(39–73)d and CRD2(39–73)de molecules. These peptides, although showing improvement in affinity in comparison to their counterparts, CRD2d and CRD2de, present significantly higher energy than CRD2(39–73)e (−45.17 ± 17.72 and −49.70 ± 17.19 for CRD2(39–73)d and CRD2(39–73)de, respectively).

Our final attempt was to design a peptide combining the best CRD1 and CRD2 variants, namely CRD1(16–38)bc and CRD2(39–73)e peptides, resulting in CRD1–2(16–73)bce. Its affinity for the LIGHT protein, although higher than the peptide based on the whole second domain (−49.53 ± 3.04 kcal/mol compared to CRD2e −35.26 ± 1.94 kcal/mol), does not show improvement compared to CRD2(39–73)e and CRD2e_K54E. Therefore, we decided to try the K54E mutation, which was determined to improve affinity in the case of CRD2 peptides. A mutant designed this way, CRD1–2(16–73)bce:K54E, exhibited an effective binding free energy only slightly higher than the CRD2e_K54E peptide. Due to our desire to design the shortest possible peptides, further research on this compound was abandoned.

### 3.4 Binding Energy and Process Captured by the SMD Simulations

**Figure 6:**
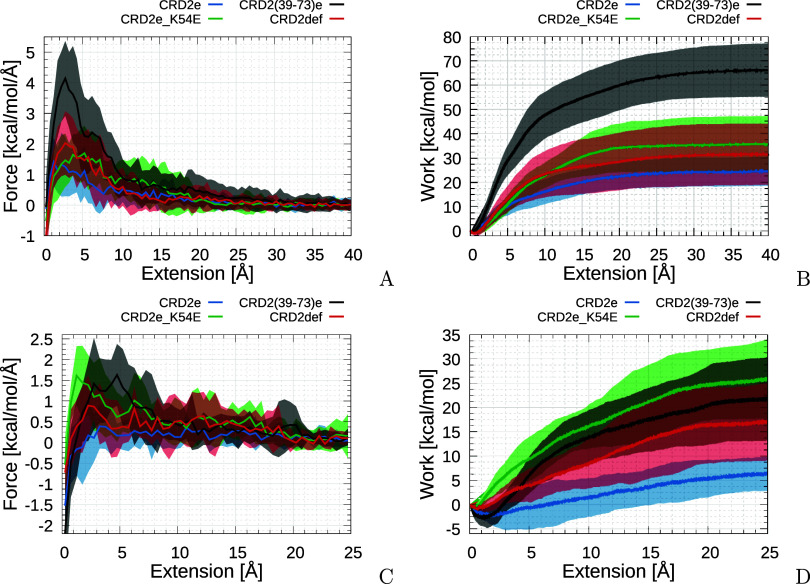
Plots of force (A, C) and work (B, D) averaged over 25 and 10 SMD trajectories (solid lines) with the corresponding standard deviation (SD) values (shown as semitransparent areas) for HVEM variants interacting with the LIGHT trimer run with 0.05 m/s (A, B) and 0.01 m/s (C, D) pulling speeds, respectively.

In order to gain a comprehensive understanding of the interactions influencing the binding between HVEM variants and LIGHT trimer, a series of 25 SMD trajectories for representative conformations of the two most promising systems and two reference systems were performed, namely CRD2e_K54E, CRD2(39–73)e, CRD2e, and CRD2def, in which the complex components were extended from each other using a spring constant on all Cα atoms to reach a full dissociation. Observed initial distances of the centers of mass were equal to 28.70, 28.21, 30.25, and 24.28 Å for CRD2e, CRD2def, CRD2e_K54E, and CRD2(39–73)e, respectively, indicating that the truncated variant formed a much more compact complex than the complete CRD2 molecules. It also suggests that the truncated amino-acid residues do not stick to the LIGHT or even prohibit other amino-acid residues from forming tight contacts. Although the CRD2e_K54E variant shows that larger work is needed for the dissociation, increased stability, compared to the CRD2e, is observed only after about 3 Å extension, and *F*_max_ values of both variants are comparable (Figure 6), whereas CRD2e_K54E reveals a smaller *F*_max_ than CRD2def. The situation is completely different when CRD2(39–73)e is taken into consideration–it shows not only a significantly larger work needed for complete dissociation than both untruncated variants (24.68 ± 6.16, 31.75 ± 12.23, 35.70 ± 11.43, and 66.18 ± 11.07 kcal/mol, for CRD2e, CRD2def, CRD2e_K54E, and CRD2(39–73)e, respectively) but also a much greater *F*_max_ value is observed (1.41 ± 0.80, 2.04 ± 1.05, 1.70 ± 0.90, and 4.14 ± 1.22 kcal/mol/Å, respectively for CRD2e, CRD2def, CRD2e_K54E, and CRD2(39–73)e). Moreover, truncation did not diminish the long-range interactions of the molecules; therefore, it should not have a negative impact on the recognition and early stages of the HVEM-fragment-LIGHT binding (Figure S10).

In CRD2e, amino-acid residues 42–44, 50–52, and 54–63 play the most important role in interacting with the LIGHT trimer when bound, with 50–54 being responsible for long-range interactions, persisting even after the extension of proteins to about 8 Å. The CRD2def exhibits a similar contact map pattern to CRD2e, with the key residues showing tighter binding. This is particularly evident for residues 132–141 of LIGHT chain B, which remained in contact even after a 12 Å extension.

Mutation of residue 54 in CRD2e_K54E impacts the binding mode to the LIGHT trimer by decreasing contacts of residues 50–52 and 54–56 of ligand. However, it allows for the strengthening of interactions by residues 57–63 and 40–44 of the ligand, enabling stabilization after a large extension to about 12 Å (compared to 8 Å in the case of nonmutated CRD2e).

For the truncated variant, namely CRD2(39–73)e, almost all amino-acid residues from CRD2(39–73)e form contacts with LIGHT without extending the molecules, especially regions 41–49, 54–68, and 71. This is also visible in Figure 5, where CRD2(39–73)e is inserted deeper into the LIGHT trimeric binding groove and no residues are sticking out outside of the complex. In addition to amino-acid residues 45–49, residues 56 and 68 are mostly responsible for the long-range interactions of CRD2(39–73)e with LIGHT. It should be noted that CRD2(39–73)e is the only variant in which almost all amino-acid residues can form contacts with LIGHT even after an extension of 22 Å, and multiple contacts are still present even if the extension exceeds 30 Å.

From the point of view of the LIGHT trimer, residues involved in the binding of all four CRD2 variants are similar, with the truncated variant forming interactions with most of the LIGHT amino-acid residues among the studied variants. While upon extension, CRD2e interacts mostly with amino-acid residues 76–93, with the help of regions around residues 160, 180, and 290, the K54E substitution pronounces the interaction with LIGHT residues 160–180 and 280–295. The binding pattern of the truncated variant, on the other hand, is much more similar to the CRD2e, forming way more interactions that are more stable during extending proteins. Overall, this suggests that point mutation K54E changes the binding mechanism, while truncation does not change the binding mechanism in the initial stages (large extension) but makes the tighter binding feasible.

Interestingly, analysis of the slower pulling speed (0.01 m/s) compared to the faster one (0.05 m/s) indicates that the CRD2(39–73)e variant exhibits much tighter binding, making it more susceptible to hydrodynamic effects upon mechanical unbinding compared to the other HVEM variants. This is evident in the larger drops in force and work observed for this variant compared to the others (Figure 6). It should also be noted that almost no Fmax drop is observed for CRD2e_K54E, indicating that only a few residues are mainly responsible for binding and no hydrodynamic effect is involved upon unbinding. During the slow pulling, the order of work needed to unbind is changed, with the CRD2e_K54E requiring the most, followed by CRD2(39–73)e. It should be noted that both the force and the work required for the CRD2e variant to dissociate become very small during slow pulling SMD, indicating that this molecule can easily dissociate, as observed in some of the longer (1000 ns) conventional MD trajectories performed for this molecule.

It should also be noted that pulling speeds used in this work (0.05 and 0.01 m/s) are significantly slower than in most computational works, which proved that SMD can predict well binding affinities of small molecules to proteins and used a pulling speed of 0.5 to 5 m/s.^60−62^

### 3.5 Equilibrium Binding Energy Presented by PMF Calculations

**Figure 7:**
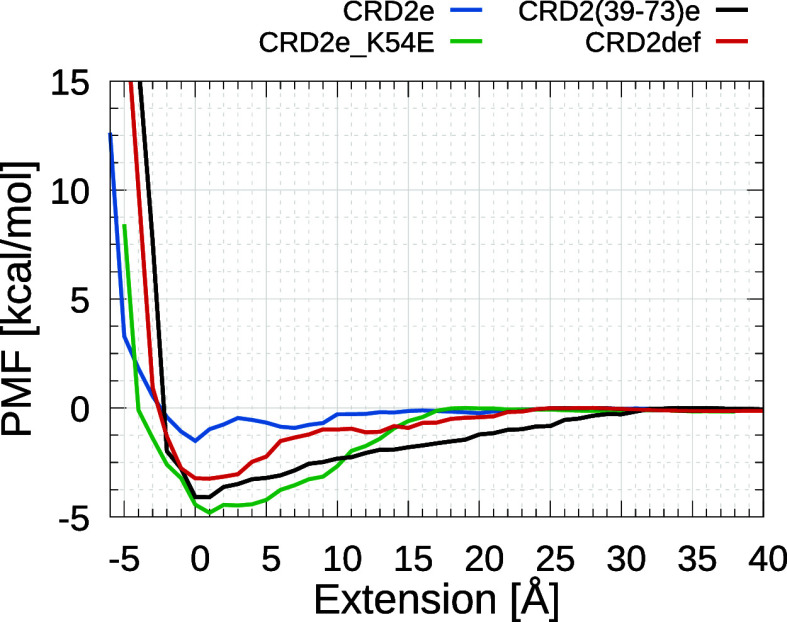
Plot of the potential of mean force (PMF) calculated based on approximately 44 trajectories for each of the HVEM variants interacting with LIGHT, obtained from umbrella sampling simulations. The extension 0 is taken as an approximation of the initial positions from conventional MD trajectories, which are used as starting points for SMD. Similarly, for PMF, it is equal to 29, 30, 24, and 28 for the centers of mass between CRD2e, CRD2e_K54E, CRD2(39–73)e, and CRD2def variants of HVEM and LIGHT, respectively.

In contrast to SMD trajectories, which depict the nonequilibrium properties of the system, PMF determination is based on conformations from the stretching trajectories but represents equilibrium phenomena. PMF results show that CRD2e_K54E is the strongest binding variant, surpassing even CRD2(39–73)e, in terms of energy difference between bound and free forms (Figure 7). It should be noted that CRD2(39–73)e exhibits a plethora of favorable positions in wider ranges of distances from the LIGHT trimer, which is most likely caused by its tightest binding.

Binding free energy computed from the PMF using eq 4 confirms raw PMF data analysis that CRD2e_K54E is the strongest binding moiety, surpassing the CRD2(39–73)e variant (computed Δ*G* is 0.37, −0.88, −1.58, and −2.44 kcal/mol for CRD2e, CRD2def, CRD2(39–73)e, and CRD2e_K54E, respectively). This trend is understandable, as it is confirmed by the same observation when a slower pulling speed was applied in the SMD approach, resulting in a drop in *F*_max_ and *W*_total_ values compared to the faster pulling speed trajectories. This change is equivalent to the transition from nonequilibrium to equilibrium simulations.

**Figure 8:**
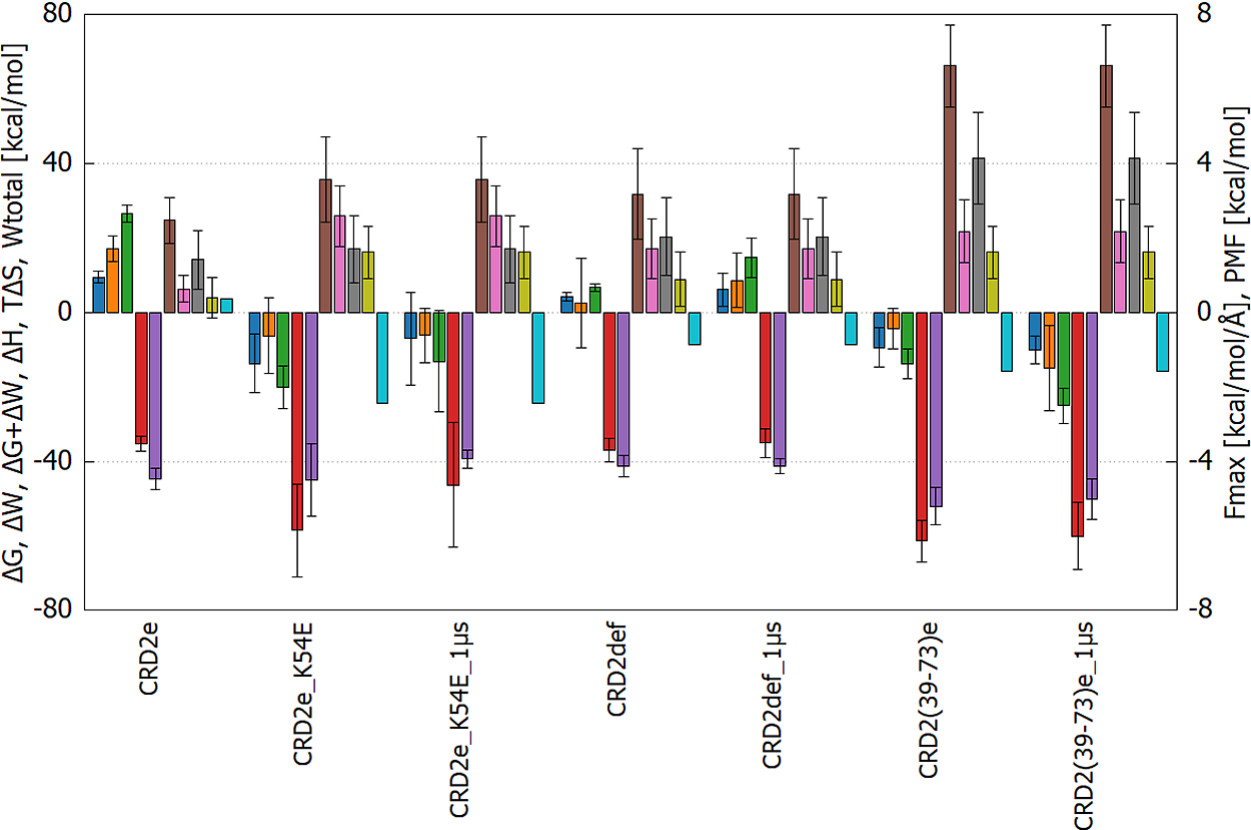
Change of: Binding free energy (Δ*G*, **blue**), internal work (Δ*W*, **orange**), summary of Gibbs free energy and internal work (Δ*G* + ΔW, **green**), enthalpy (Δ*H*, **red**), entropy (*T*Δ*S*, **violet**) in 200 and 1000 ns long simulations (”1 μs” postscript), total work (*W*_total_, pulling speed 0.05 m/s **brown**, 0.01 m/s **pink**), force max (*F*_max_, pulling speed 0.05 m/s **gray**, 0.01 m/s **yellowish green**), and potential mean force (PMF, **cyan**) calculated on SMD basis of four selected systems. CRD2e_1 μs, due to dissociating, is not shown.

### 3.6 Changes in Binding Free Energy, Internal Work, Enthalpy, and Entropy Due to Complex Formation

**Figure 9:**
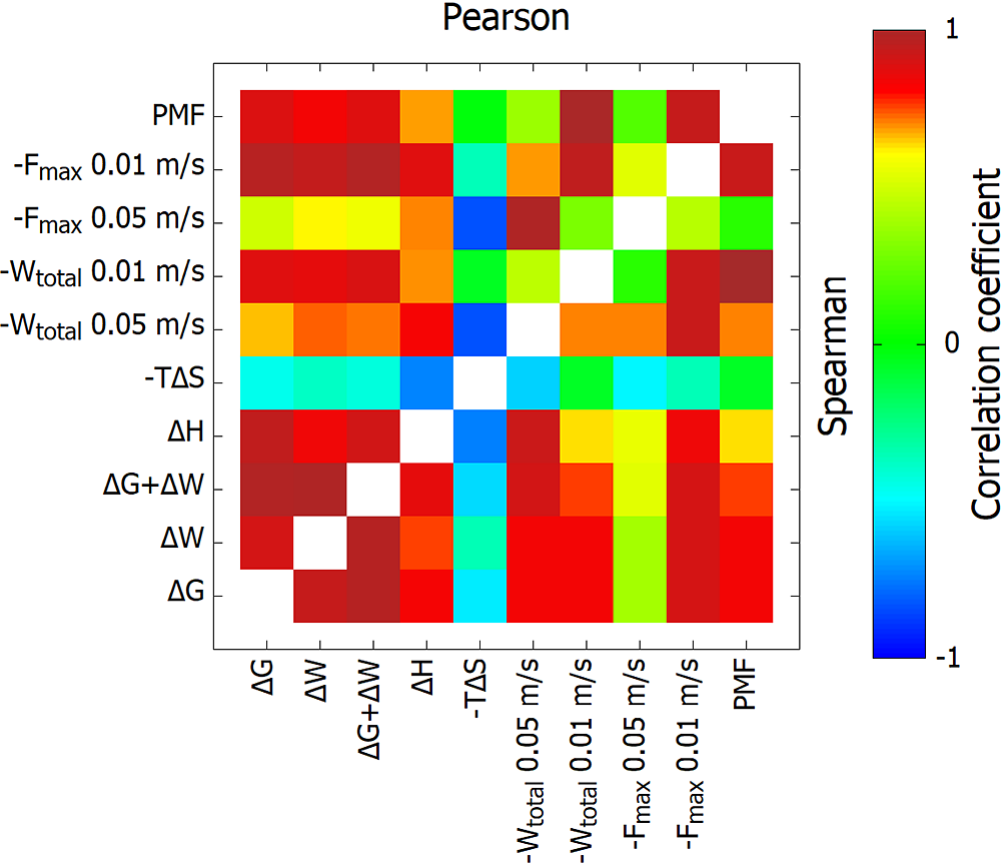
Pearson and Spearman correlation coefficients of the properties computed by various methods to calculate energetic components contributing to the binding affinity of the complexes determined for the systems included in Figure S8.

To evaluate the relationship between different techniques in predicting binding energy, we compared their results and found that fast pulling SMD aligns well with MM-GBSA, while slow SMD aligns well with PMFs (Figures 8 and 9). For comparison, we included all the results for strong binding peptides in the Supporting Information (Figure S11). We found that CRD2e is the least stable among the closely studied peptides. In the case of the CRD2def molecule, defined as a reference structure, binding free energy (Δ*G*) and work transition between free and bound states of the ligand (Δ*W*) are also positive, while for CRD2e_K54E and CRD(39–73)e, they are both negative. Moreover, these values tend to increase during simulations (as indicated in CRD2def_1 μs).

In the case of the CRD2e peptide, the stability of its complex with LIGHT is even weaker. In the best mutant, CRD2e_K54E, Δ*W* tends to remain at the same level during longer simulations, but Δ*G* and enthalpic difference (Δ*H*) increase, indicating that the complex is losing its stability over the course of the simulation. The increased affinity to LIGHT, marked by a lower Δ*H* value, may originate from new interactions, but they have little impact on the overall stability of the complex.

In the shortened molecule, CRD2(39–73)e, a different pattern is observed: Δ*W* decreases in longer simulations, indicating further stabilization of the molecule structure as a result of complex formation. The entropic contribution (*T*Δ*S*) is significantly larger (more negative) than other counterparts, but this effect is compensated by enthalpy, and therefore, Δ*G* + Δ*W* is more negative than other studied variants. The maximum force peak (*F*_max_) and total work needed for dissociation (*W*_total_) values are similar in CRD2e, CRD2def, and CRD2e_K54E, but in CRD2(39–73)e, they are visibly more favorable, further suggesting the highest affinity for the receptor. However, this trend is no longer visible in the case of PMFs and slower SMD simulations.

This indicates that MM-GBSA overemphasizes the solvent component, while fast SMD depends on the hydrodynamic properties as water cannot accommodate overly rapid pulling. When PMF or slow SMD results are analyzed, CRD2e_K54E emerges as the strongest binding peptide; however, PMF and SMD seem to overemphasize a single strong interaction. Therefore, both CRD2e_K54E and CRD2(39–73)e are great HVEM-LIGHT inhibitor candidates, albeit through different properties.

Strong positive correlation between Δ*G*, Δ*W*, Δ*G* + Δ*W,* and Δ*H* with −*W*_total_, −*F*_max,_ and PMF implies that all methods used provide reliable estimation of binding energy. Those values are related due to the fact that with the decrease of the Gibbs free energy and enthalpy, the strength of the peptide binding to the protein increases, which is expressed by the increase in the work and force needed to detach the peptide from the protein marked by SMD. PMF correlates very well with both *W*_total_ and *F*_max_ from the slow pulling SMD simulations. This suggests that slow SMD simulations capture the equilibrium properties of the system better than fast SMD, aligning well with the PMF results.

Anticorrelation of Δ*G*, Δ*W*, Δ*G* + Δ*W,* and Δ*H* with a negative *T*Δ*S* value should be noted, which could be interpreted as the more strongly the ligand binds to the receptor, the more negative the *T*Δ*S* term becomes, meaning the structure becomes more rigid upon binding.

The weakest correlation was observed for −*F*_max_ for fast pulling; therefore, it is not recommended to rely solely on this property when assessing binding affinities using SMD simulations. This is in agreement with previous observations that total work is more accurate estimation of the binding affinity than the main force peak.^63^

Comparing the Pearson and Spearman correlation coefficients, we observe that they are generally in good agreement. However, in some cases, the Spearman coefficient is slightly higher, suggesting a stronger monotonic relationship between the variables, even if the linear correlation (captured by Pearson) is not as strong. This highlights the robustness of the Spearman coefficient in capturing nonlinear relationships between the binding energy estimates from different methods.

Notably, the correlation coefficients between PMF and *W*_total_ for slow pulling are 0.94 (Pearson) and 0.90 (Spearman), indicating a very strong agreement between these two methods. In contrast, the correlation coefficients between PMF and *W*_total_ for fast pulling are lower, with values of 0.77 (Pearson) and 0.80 (Spearman). This further supports the notion that slow SMD simulations better capture the equilibrium properties of the system compared to fast SMD simulations.

### 3.7 Designed Truncated Peptide Is Highly Selective to the LIGHT Binding Groove

As the LIGHT trimer consists of three identical binding sites, the analysis of the HDOCK results reveals that CRD2(39–73)e is docked to these three positions in its top 10 binding modes, with one of these modes ranking as ‘top1’. This finding suggests that CRD2(39–73)e and HVEM can effectively compete for the same binding site on the LIGHT trimer, therefore inhibiting the HVEM-LIGHT complex formation. This, along with the docking score of −342.41 and confidence score of 0.9791, shows a strong preference of the CRD2(39–73)e peptide to bind to the LIGHT trimer.

As this study primarily focuses on silencing the stimulatory signal with LIGHT as opposed to the inhibitory signal with BTLA, we also docked the best molecule candidates to BTLA. Docking of the CRD2(39–73)e peptide to the BTLA dimer shows low specificity, as the molecules in the top 10 binding modes are located in the interface between the molecules with the best docking score of −239.82 and confidence score of 0.8577, which are significantly worse than for the binding with the LIGHT trimer. CRD2e_K54E and CRD2e show worse docking (−252.88 and −213.45, respectively) and confidence (0.8867 and 0.7806, respectively) scores when binding to LIGHT, as well as lower selectivity compared to binding to the BTLA molecule, making CRD2(39–73)e the best drug candidate. Moreover, calculated lRMSD values, representing the position of CRD2e_K54E and CRD2(39–73)e in the LIGHT binding groove, were the lowest (most stable) among all tested variants (Figure S12), further indicating high selectivity. Additionally, we confirmed the binding sites using the coarse-grained UNRES force field and the UNRES-Dock procedure. The results showed that CRD2e, CRD2e_K54E, CRD2(39–73)e, and CRD2def reveal similar binding sites as observed in the all-atom simulations (Figure S14).

## 4 Discussion

The crystal structure of the HVEM/LIGHT complex (PDB: 4RSU)^9^ shows a 3:3 stoichiometry. Crystallographic studies confirmed that CRD2, CRD3, and a small part of CRD1 of HVEM are involved in the interactions with LIGHT. CRD1 and CRD2 of HVEM interact with the loop regions G100, G151, T170-E175, L177, V255, and R226-G230 of LIGHT, while CRD3 of HVEM binds to loop regions: G151-V152, A159-T161, Q183, R195-V196, and W198 from LIGHT. It should be noted that HVEM is located between two LIGHT proteins (Figure 1). In our study, residues from both binding loop regions (T170-E175 and R226-G230) are in the top 5 amino-acid residues for the most promising variants (Table 1).

**Table 1:** Top 5 Amino-Acid Residues from CRD2e, CRD2e_K54E, and CRD2(39-73)e and LIGHT Protein in Complexes, Ranked Based on Their Effective Binding Free Energy Decomposition [kcal/mol] Determined by MM-GBSA Pairwise Analysis Averaged Over Three Independent Trajectories with Standard Deviation^a^

CRD2e	CRD2e_K54E	CRD2def	CRD2(39–73)e
Gln57 −9.04 ± 3.67	Met60 −11.76 ± 2.94	Gln57 −8.07 ± 3.13	Gln57 −13.90 ± 2.70
Lys54 −8.09 ± 4.20	Gln57 −11.34 ± 3.99	Lys54 −4.70 ± 4.34	Lys54 −10.21 ± 8.46
Met60 −4.74 ± 2.15	Asp62 −8.48 ± 3.60	Met60 −4.34 ± 1.75	Met65 −8.01 ± 3.62
Met65 −3.87 ± 3.11	Gln59 −7.94 ± 2.34	Pro63 −2.61 ± 2.00	Arg68 −6.03 ± 5.10
Gln59 −2.95 ± 1.86	Met65 −6.28 ± 2.03	Ala64 −2.25 ± 1.46	Gln59 −5.87 ± 2.86

LIGHT	LIGHT	LIGHT	LIGHT
Tyr173(A) −7.79 ± 3.84	Asn102(B) −11.02 ± 3.37	Asn102(B) −6.34 ± 1.64	Glu178(A) −10.23 ± 5.61
Asn102(B) −6.65 ± 3.40	Arg228(B) −10.56 ± 3.06	Leu118(B) −3.99 ± 1.48	Leu118(B) −8.07 ± 2.38
Arg228(B) −6.21 ± 2.71	Arg226(B) −9.48 ± 2.91	Arg228(B) −2.81 ± 1.77	Gly119(B) −7.66 ± 2.65
Leu118(B) −5.58 ± 2.89	Ser103(B) −6.78 ± 2.39	Ser104(B) −2.17 ± 0.99	Arg228(B) −6.93 ± 1.67
Glu175(A) −4.66 ± 2.67	Arg172(A) −6.47 ± 5.82	Glu115(B) −2.10 ± 1.07	Arg172(A) −5.72 ± 4.90

a As LIGHT is a homotrimer and HVEM variants interact with two of its chains, a chain letter (A–C) is presented in brackets.

During the last several years, many single and double mutants of HVEM proteins were designed, and their interactions with LIGHT were tested. Shrestha et al. computationally redesigned the HVEM recognition interfaces using a residue-specific pharmacophore approach and postulated that single mutations H48I, D62R, M65K, and double mutations H48I/M65K or D62R/M65K in CRD2 of HVEM significantly reduced binding to LIGHT or resulted in a loss of interactions. According to the structural modeling, the substitution of methionine 65 for lysine in HVEM delivers unfavorable electrostatic interactions with R226 in LIGHT, while the mutation H48I disrupts favorable polar interactions between H48 and E175 and R226 in LIGHT. The other single mutations in HVEM, such as D7F, E14R, E14K, S20L, S20K, S20Q, E31R, L32D, L49F, L40W, G51F, and V91K, and double mutations S20Q_L32D and E31R_G51F, do not have significant effects on the binding to LIGHT.^64^ Cheung et al. pointed out that Y9F, S20A, Y23F, R24A, K26A, E27A, and E38A in CRD1 of HVEM and R75A in CRD2 do not affect binding to LIGHT.^65^

The performed effective binding energy decomposition (Figure S9) explains why experimental mutations of the selected amino-acid residues Y9, S20, Y23, R24, K26, E27, E38, R75,^65^ and D7, E14, S20, E31, and L32^64^ do not affect the binding affinity to LIGHT, due to their near-zero contribution to LIGHT binding and presence in regions that are not directly involved in binding. We found that L52 and M60 play an important role in the binding interface for the complete HVEM molecule (Figure S9, Table 1), while other experimentally determined amino-acid residues,^9^ such as H48 and L56, are in their vicinity and may play a role in stabilizing the local structure of HVEM rather than playing a role in the formation of direct interactions. This is further confirmed by the observation of Liu et al. that only pairwise mutagenesis of H48, L52, and L56 has an observable effect on LIGHT binding.

Shrestha et al. showed that the G51F mutant of HVEM binds to LIGHT comparably to wt HVEM. Residue 51 of HVEM forms contact with R172 of LIGHT through their backbone atoms (as mentioned above), and therefore, this substitution is not so important for protein binding.^66^ This work is a good example that not all mutations of the amino-acid residues involved in the interaction with the partner molecule have a significant influence on the total binding affinity. In our study, we observed similar behavior for some of the substitutions of K54, C55, C61, D62, and N72, while some other amino-acid residues, such as P39, are much more sensitive to substitution. Overall, this proves that MM-GBSA with pairwise and per-residue decomposition can be a useful tool that explains the key interactions and mutation effects.

## 5 Conclusions

In this study, we conducted a comprehensive investigation into the stability of the HVEM molecule, its domains, and domain fragments, as well as their interactions with the LIGHT trimer, to identify potential strategies for modulating LIGHT activity. Our extensive MD simulations, totaling over 175 μs of production runs, provided valuable insights into the role of disulfide bonds in domain stability and their varying stabilizing properties. While the addition of disulfide bonds generally increases protein stability, it is important to note that this is not always the case, as exemplified by peroxiredoxin enzymes, where disulfide bond formation can introduce structural frustration and increased dynamics.^67^

Our simulations confirmed CRD2 as a key fragment for interactions with LIGHT, in agreement with experimental observations. The presence of disulfide bonds emerged as a critical determinant of HVEM domain stability, with specific combinations exerting varying impacts on structural integrity. Interestingly, we found that in some cases, such as CRD2no, the lack of a disulfide bond allows the structure to open up and adjust during binding to LIGHT, resulting in increased flexibility and binding strength compared to CRD2all (Figures S4, S5, S7).

Through MM-GBSA analyses, we successfully identified promising peptide inhibitors targeting the LIGHT trimer, with the CRD2 domain serving as the key binding site. Mutational and truncational studies of the CRD2e peptide provided valuable insights into enhancing binding affinity, albeit with intriguing complexities that warrant further investigation. Steered MD simulations at various pulling speeds revealed dynamic insights into the binding and dissociation events between HVEM fragments and LIGHT, highlighting the influence of specific amino-acid residue truncations on complex stabilization without impacting molecular recognition.

Our study advances the understanding of the molecular basis of HVEM-LIGHT interactions and HVEM domain stability, contributing to the development of targeted therapeutic interventions for immune-related disorders. We propose a truncated variant, CRD2(39–73)e, and a mutant, CRD2e_K54E, as the most promising compounds for selective interaction with the LIGHT trimer. Our results can be used for further experimental verification, as demonstrated in our previous work.^20^

Importantly, our studies revealed that each computational technique has its own limitations in predicting binding affinities. MM-GBSA tends to overemphasize the solvent component, while fast SMD simulations are heavily influenced by hydrodynamic properties, as water molecules cannot effectively enter the cavity formed during protein dissociation, leading to an overestimation of binding affinity for more tightly bound complexes. This effect can be mitigated by using slower pulling speeds, albeit at the cost of increased computational resources. Conversely, PMF and SMD, when used to study binding affinities, may overemphasize the contribution of single strong interactions. Therefore, we recommend using these techniques in a complementary manner to gain a comprehensive understanding of the system. By combining insights from multiple methods, one can obtain a more complete picture of binding affinities and the underlying mechanisms that give rise to the differences observed between various systems. This multifaceted approach ensures a thorough analysis of the protein–protein interactions under investigation.

In conclusion, our multistep study of structural properties, binding affinity, selectivity, and mechanisms sets a new standard for the computational design of peptide drugs, while the insights gained from this work pave the way for the development of novel therapeutic strategies targeting the HVEM-LIGHT interaction with potential applications in the treatment of immune-related disorders.

## Data Availability

Three PDB models of the LIGHT inhibitors: PM0084527, PM0084528, and PM0084592.

## Supporting Information Available

Table with names and amino-acid sequences of designed peptides and simulation details; estimated secondary structure content; solvent accessible surface area (SASA) and hydrophobic SASA; plots of distance between sulfur atoms in disulfide bonds; structural properties comparisons; experimental and simulated CD spectra; heatmaps for SASA and RMSF; change in secondary structures heatmap; native contacts between HVEM variants and LIGHT trimer; effective free energies bar plot; MM-GBSA binding energy decomposition results; contact heatmaps for SMD simulations; plot of binding free energies; structural properties bar plots; and structural comparison between all-atom and UNRES-dock structures (PDF)
