# Supplementary material for: Multilayered Computational Framework for Designing Peptide Inhibitors of HVEM-LIGHT Interaction
Source: J Phys Chem B. 2024 Jul 3;128(28):6770–85. doi: 10.1021/acs.jpcb.4c02255 (PMC11264271; doi:10.1021/acs.jpcb.4c02255)
Supplement: Supplementary file 1 — jp4c02255_si_001.pdf [file jp4c02255_si_001.pdf]

Supporting Information for ”Multi-Layered Computational Framework for Designing Peptide Inhibitors of HVEM-LIGHT Interaction”

Piotr Ciura[1], Pamela Smardz[2], Marta Spodzieja[1], Adam K. Sieradzan*[1], and Pawel Krupa[2]

[1]Faculty of Chemistry, University of Gdańsk, Fahrenheit Union of Universities in Gdańsk, Bażyńskiego 8, 80-309, Gdańsk, Poland
[2]Institute of Physics, Polish Academy of Sciences, Al. Lotników 32/46, 02-668, Warsaw, Poland

1 Influence of disulfide bonds on CRD stability

The HVEM domains demonstrate a very different role of particular disulfide bonds on their stability. In CRD1, out of all simulations with only one bond, Cys16-Cys29 (marked as b) lowered RMSD to 7.47Å from 11.20Å in simulations without bonds. Yet, only when coupled with Cys19-Cys29 (c), the RMSD lowers to 3.27Å, which is close to the result of simulations with all bonds present (2.89Å). Both the combinations ”ab” and ”ac” have results comparable to the one bond ”b”. It can be concluded that ”a” has the smallest importance in the overall stability of CRD1.

In CRD3, which has up to two disulfide bonds, the presence of Cys83-Cys100 (g) has a greater effect on stability (3.89 Å) than the presence of Cys89-Cys97 (h). The measured end-to-end distance for CRD3h is comparable to that without bonds, and for CRD3g, it is comparable to all bonds present. The RMSF of residues in both the C- and N-terminal in simulations without (g) are much higher compared to those with (h) present (Fig. S5).

Further increase in the stability of the domain was observed when the rest of the HVEM was present, indicating that these are rather protein fragments than structurally independent moieties.

The radius of gyration (RG) and RGmax analyses provide additional confirmation that, for CRD1, the most crucial bond is (b). This is evident from the similarity in values between simulations where it is present and those where all bonds are present, indicating a consistent level of compactness. In contrast, the compactness of CRD2 was not significantly affected by the presence or absence of disulfide bonds, except for CRD2no and CRD2d (Fig. S2), which exhibited a more relaxed or loose structure, in comparison to the structures with disulfide bonds in position (e) and (f). Regarding CRD3, disulfide bond (g) appears to contribute more significantly to structural stabilization than (h). It is worth noting that for molecules without disulfide bonds the rigidify occurs upon binding. The higher the bonding affinity, the more structure rigidity is observed (Fig S2, S3). The SASA of bound and unbound HVEM variants (Fig. S4) are similar (the variants bound to LIGHT trimer have slightly larger SASA), this indicate that hydrophobic interaction are not the key players in HVEM-LIGHT interactions.

*adam.sieradzan@ug.edu.pl

List of Figures

S1 Distance between S atoms in disulfide bonds averaged for 3 trajectories for the second halves of the simulations (500-1000ns) for CRD1, CRD2 and CRD3 without the LIGHT trimer. S10
S2 (A) RMSD, (C) RG, (E) RGmax, (G) End-to-end distance calculated from simulations of free domains and (B) RMSD, (D) RG, (F) RGmax and (H) End-to-end distance values calculated from simulations with LIGHT, averaged over the second halves of the 3 trajectories. S11
S2 continued: (I) SASA values calculated from simulation of free domains and (J) theoretical SASA values calculated from simulation with the LIGHT trimer (the LIGHT trimer was excluded in this analysis, therefore, the whole HVEM variant surface was computed) averaged over the second halves of the 3 trajectories. . . . S12
S3 Experimental (A) and simulated (B) CD spectra of the selected designed peptides. S12
S4 Heatmap of per-residue $\Delta SASA = SASA_{\text{free}} - SASA_{\text{complex}}$ (right-hand bar [Å^2]) calculated for all combinations of disulfide bonds present and absent in the simulations for the full HVEM molecule, its domains (CRD1-3) and it variant, and CRD2e_K54E, and fragment (CRD(39-73)e), averaged over the second halves of the 3 trajectories. S13
S5 Heatmap of per-residue $\Delta RMSF = RMSF_{\text{free}} - RMSF_{\text{complex}}$ (right-hand bar [Å]) calculated for HVEM with and without disulfide bonds, and its selected variants forming stable complexes with LIGHT, averaged over the second halves of the 3 trajectories. S14
S6 Heatmap of the change in the content of secondary structures expressed in percentage points calculated for HVEM with and without disulfide bonds, and its selected variants forming stable complexes with LIGHT, averaged over the second halves of the 3 trajectories S15
S7 Number of native contacts between HVEM variant and LIGHT trimer as a function of time for last 20ns for (A): CRD1no (red), CRD1a (blue), and CRD1b (grey); (B): CRD1c (red), CRD1ab (blue), and CRD1ac (grey); (C): CRD1bc (red), CRD1abc (blue), and CRD1(16-38)no (grey); (D): CRD1(16-38)bc (red), CRD1-2(16-73)bce (blue), and CRD1-2(16-73)bce_K54E (grey) (E): CRD2no (red) CRD2no_K54E (blue), CRD2no_K54D (grey); (F): CRD2no_K54I (red); (F): CRD2no_K54L (blue), and CRD2no_K54S (grey); (G): CRD2no_K54V (red) CRD2no_K54Y (blue), and CRD2d (grey); (H): CRD2e (red), CRD2e_K54E (blue), and CRD2e_K54D (grey). Different trajectories are depicted as various color tone. S16
S7 continued: (I): CRD2e_K54I (red), CRD2e_K54L (blue), and CRD2e_K54S (grey); (J) CRD2e_K54V (red), CRD2e_K54Y (blue), and CRD2e_K54E_D62A (grey); (K): CRD2e_K54E_D62K (red), CRD2e_K54E_D62L (blue), and CRD2e_K54E_D62S (grey); (L): CRD2f (red); CRD2de (blue) and CRD2de_K54E (grey); (M): CRD2de_K54D (red), CRD2de_K54I (blue), and CRD2de_K54L (grey); (N): CRD2de_K54S (red), CRD2df (blue), and CRD2ef (grey); (O): CRD2def (red), CRD2(39-73)d (blue), and CRD2(39-73)de (grey); (P): CRD2(39-73)e (red), CRD2(39-73)e_K54E(blue), and CRD2(39-73)e_P39A (grey). Different trajectories are depicted as various color tone. S17

S7 continued (Q): CRD2(39-73)e_P39V(red), CRD2(39-73)e_P39W (blue), and CRD2(39-73)e_P39L (grey); (R): CRD2(39-73)e_P39I (red), CRD2(39-73)e_P39x(blue), and CRD2(39-73)e_N72A (grey); (S): CRD2(39-73)e_N72G(red), CRD2(39-73)e_C55A (blue), and CRD2(39-73)e_C55V (grey); (T): CRD2(39-73)e_C61S (red), CRD2(39-73)no (blue) and CRD3no (grey); (U): CRD3g (red), CRD3h (blue) and CRD3gh(grey); (V): CRD3(89-97)no (red), and CRD3(89-97)h (blue) . Different trajectories are depicted as various color tone. S18
S8 Bar plot of the effective free energies of the various stable variants of the HVEM molecule interacting with the LIGHT trimer. S19
S9 MM-GBSA effective binding energy decomposition results for (A) CRD1no, (B) CRD1(16-38)no, (C) CRD1bc, (D) CRD1-2(16-73)bce, (E) CRD1-2(16-73)bce_K54E, (F) CRD2no variants S20
S9 continued: MM-GBSA effective binding energy decomposition results for (G) CRD2no_K54D, (H) CRD2no_K54I, (I) CRD2no_K54L, (J) CRD2no_K54S, (K) CRD2no_K54V, (L) CRD2no_K54Y variants S21
S9 continued: MM-GBSA effective binding energy decomposition results for: (M) CRD2d, (N) CRD2e, (O) CRD2e_K54D, (P) CRD2e_K54E, (Q) CRD2e_K54I, (R) CRD2e_K54L variants S22
S9 continued: MM-GBSA effective binding energy decomposition results for: (S) CRD2e_K54S, (T) CRD2e_K54V, (U) CRD2e_K54Y, (V) CRD2e_K54E_D62A, (W) CRD2e_K54E_D62K, (X) CRD2e_K54E_D62L variants S23
S9 continued: MM-GBSA effective binding energy decomposition results for: (Y) CRD2f, (Z) CRD2de, (AA) CRD2de_K54D, (AB) CRD2de_K54E, (AC) CRD2de_K54I, (AD) CRD2de_K54S variants S24
S9 continued: MM-GBSA effective binding energy decomposition results for: (AE) CRD2df, (AF) CRD2ef, (AG) CRD2def, (AH) CRD2(39-73)no, (AI) CRD2(39-73)d, (AJ) CRD2(39-73)e variants S25
S9 continued: MM-GBSA effective binding energy decomposition results for: (AK) CRD2(39-73)e_P39A, (AL) CRD2(39-73)e_P39I, (AM) CRD2(39-73)e_P39L, (AN) CRD2(39-73)e_P39V, (AO) CRD2(39-73)e_P39W, (AP) CRD2(39-73)e_P39x variants S26
S9 continued: MM-GBSA effective binding energy decomposition results for: (AQ) CRD2(39-73)e_K54E, (AR) CRD2(39-73)e_C55A, (AS) CRD2(39-73)e_C55V, (AT) CRD2(39-73)e_C61S, (AU) CRD2(39-73)e_N72A, (AV) CRD2(39-73)e_N72G variants S27
S9 continued: MM-GBSA effective binding energy decomposition results for: (AW) CRD2(39-73)de, (AX) CRD3no, (AY) CRD3(39-73)no S28
S9 continued: MM-GBSA effective binding energy decomposition results for: (AZ) HVEMno variant, (AAA) HVEMall S29
S10 Contact heatmap averaged over 25 SMD simulations for HVEM variant CRD2e with the LIGHT trimer. The values are normalized in the range of increasing extension (relative difference of the distance between centers of mass of HVEM variant and LIGHT trimer) averaged for 4 Å. S30
S10 continued: Contact heatmap averaged over 25 SMD simulations for HVEM variant CRD2def with the LIGHT trimer. The values are normalized in the range of increasing extension (relative difference of the distance between centers of mass of HVEM variant and LIGHT trimer) averaged for 4 Å. S31
S10 continued: Contact heatmap averaged over 25 SMD simulations for HVEM variant CRD2(39-73)e with the LIGHT trimer. The values are normalized in the range of increasing extension (relative difference of the distance between centers of mass of HVEM variant and LIGHT trimer) averaged for 4 Å. S32

S10 continued: Contact heatmap averaged over 25 SMD simulations for HVEM variant CRD2e_K54E with the LIGHT trimer. The values are normalized in the range of increasing extension (relative difference of the distance between centers of mass of HVEM variant and LIGHT trimer) averaged for 4 Å. S33
S10 Representative structures upon extending (relative difference of the distance between centers of mass of HVEM variant and LIGHT trimer) averaged for 4 Å. . . S34
S11 Change of: Binding free energy (ΔG, **blue**), internal work (ΔW, **orange**), summary of Gibbs free energy and internal work (ΔG+ΔW, **green**), enthalpy (ΔH, **red**), entropy (ΔS, **violet**) in 200 ns and 1000 ns long simulations ("1μs" postscript), total work (Wtotal, pulling speed 0.05 m/s **brown**, 0.01 m/s **pink**), force max (Fmax, pulling speed 0.05 m/s gray, 0.01 m/s yellowish green) and potential mean force (PMF, **cyan**) calculated on SMD basis of eleven selected systems with highest affinity to LIGHT. CRD2e_1μs, due to dissociating, is not shown. S35
S12 Bar plots of the stable complexes of the structural properties: (A) Complex CαRMSD, (B) Receptor CαRMSD, and (C) Ligand CαRMSD values averaged for 3 trajectories for the last 20 ns of simulation. For the RMSD calculation, an initial conformation adapted from the PDB file was used as a reference. S36
S12 continued: Bar plots of the stable complexes of the structural properties: (D) RG and (E) RGmax values averaged for 3 trajectories for the last 20 ns of simulation. For the RMSD calculation, an initial conformation adapted from the PDB file was used as a reference. S37
S12 continued: Bar plots of the stable complexes of the structural properties: (F) End-to-end distance and (G) lRMSD values averaged for 3 trajectories for the last 20 ns of simulation. For the RMSD calculation, an initial conformation adapted from the PDB file was used as a reference. S38
S13 Number of native contacts between HVEM variant and LIGHT trimer as a function of time for 1μs simulations (A): CRD2e (red), CRD2e_K54E (blue), and CRD2(39-73)e (grey); (B): CRD2def (red). Different trajectories are depicted as various color tone. S38
S14 Comparison between the all-atom dominant structure (left, cartoon representation) with the UNRES-dock cluster with lowest RMSD to respective all-atom structure (right, ribbon representation). Each LIGHT chain is colored green, cyan, magenta and peptide is colored in red for: A) CRD2e (RMSD 4.80Å), B) CRD2def (RMSD 4.39Å), C) CRD2_K54E (RMSD 6.30Å) and D) CRD2(39-73)e (RMSD 4.95Å). S39

Table S1: Names and amino acid sequences of the designed peptides

name	disulfide bonds	residues
CRD1no	without	L^{1}PSCKEDEYPV^{11}GSECCPKCSP^{21}GYRVKEACG^{31}ELTGTVCE
CRD1a	C^{4}-C^{15}	L^{1}PS**C**KEDEYPV^{11}GSE**C**CPKCSP^{21}GYRVKEACG^{31}ELTGTVCE
CRD1b	C^{16}-C^{29}	L^{1}PSCKEDEYPV^{11}GSEC**C**KCSP^{21}GYRVKEA**C**G^{31}ELTGTVCE
CRD1c	C^{19}-C^{37}	L^{1}PSCKEDEYPV^{11}GSECCPK**C**SP^{21}GYRVKEACG^{31}ELTGTV**C**E
CRD1ab	C^{4}-C^{15};C^{16}-C^{29}	L^{1}PS**C**KEDEYPV^{11}GSE**CC**PKCSP^{21}GYRVKEA**C**G^{31}ELTGTVCE
CRD1ac	C^{4}-C^{15};C^{19}-C^{37}	L^{1}PS**C**KEDEYPV^{11}GSE**C**CPK**C**SP^{21}GYRVKEACG^{31}ELTGTV**C**E
CRD1bc	C^{16}-C^{29};C^{19}-C^{37}	L^{1}PSCKEDEYPV^{11}GSEC**C**PK**C**SP^{21}GYRVKEA**C**G^{31}ELTGTV**C**E
CRD1abc	C^{4}-C^{15};C^{16}-C^{29};C^{19}-C^{37}	L^{1}PS**C**KEDEYPV^{11}GSE**CC**PK**C**SP^{21}GYRVKEA**C**G^{31}ELTGTV**C**E
CRD1(16-38)no	without	CPKCSP^{21}GYRVKEACG^{31}ELTGTVCE
CRD1(16-38)bc	C^{16}-C^{29};C^{19}-C^{37}	**C**PK**C**SP^{21}GYRVKEA**C**G^{31}ELTGTV**C**E
CRD1-2(16-73)bce	C^{16}-C^{29};C^{19}-C^{37};C^{58}-C^{73}	**C**PK**C**SP^{21}GYRVKEA**C**G^{31}ELTGTV**C**EPCP^{41}PGTYIHLNG^{51}LSKCLQ**C**QMC^{61}DPAMGLRASR^{71}N**C**^{73}
CRD1-2(16-73)bce_K54E	C^{16}-C^{29};C^{19}-C^{37};C^{58}-C^{73}	**C**PK**C**SP^{21}GYRVKEA**C**G^{31}ELTGTV**C**EPCP^{41}PGTYIHLNG^{51}LSECLQ**C**QMC^{61}DPAMGLRASR^{71}N**C**^{73}
CRD2no	without	PCP^{41}PGTYIHLNG^{51}LSKCLQCQMC^{61}DPAMGLRASR^{71}NCSRTENAVC^{81}
CRD2no_K54D	without	PCP^{41}PGTYIHLNG^{51}LSDCLQCQMC^{61}DPAMGLRASR^{71}NCSRTENAVC^{81}
CRD2no_K54E	without	PCP^{41}PGTYIHLNG^{51}LSECLQCQMC^{61}DPAMGLRASR^{71}NCSRTENAVC^{81}
CRD2no_K54I	without	PCP^{41}PGTYIHLNG^{51}LSICLQCQMC^{61}DPAMGLRASR^{71}NCSRTENAVC^{81}
CRD2no_K54L	without	PCP^{41}PGTYIHLNG^{51}LSLCLQCQMC^{61}DPAMGLRASR^{71}NCSRTENAVC^{81}
CRD2no_K54S	without	PCP^{41}PGTYIHLNG^{51}LSSCLQCQMC^{61}DPAMGLRASR^{71}NCSRTENAVC^{81}
CRD2no_K54V	without	PCP^{41}PGTYIHLNG^{51}LSVCLQCQMC^{61}DPAMGLRASR^{71}NCSRTENAVC^{81}
CRD2no_K54Y	without	PCP^{41}PGTYIHLNG^{51}LSYCLQCQMC^{61}DPAMGLRASR^{71}NCSRTENAVC^{81}
CRD2d	C^{40}-C^{55}	P**C**P^{41}PGTYIHLNG^{51}LSK**C**LQCQMC^{61}DPAMGLRASR^{71}NCSRTENAVC^{81}
CRD2e	C^{58}-C^{73}	PCP^{41}PGTYIHLNG^{51}LSKCLQ**C**QMC^{61}DPAMGLRASR^{71}N**C**SRTENAVC^{81}
CRD2e_K54D	C^{58}-C^{73}	PCP^{41}PGTYIHLNG^{51}LSDCLQ**C**QMC^{61}DPAMGLRASR^{71}N**C**SRTENAVC^{81}
CRD2e_K54E	C^{58}-C^{73}	PCP^{41}PGTYIHLNG^{51}LSECLQ**C**QMC^{61}DPAMGLRASR^{71}N**C**SRTENAVC^{81}
CRD2e_K54I	C^{58}-C^{73}	PCP^{41}PGTYIHLNG^{51}LSICLQ**C**QMC^{61}DPAMGLRASR^{71}N**C**SRTENAVC^{81}
CRD2e_K54L	C^{58}-C^{73}	PCP^{41}PGTYIHLNG^{51}LSLCLQ**C**QMC^{61}DPAMGLRASR^{71}N**C**SRTENAVC^{81}
CRD2e_K54S	C^{58}-C^{73}	PCP^{41}PGTYIHLNG^{51}LSSCLQ**C**QMC^{61}DPAMGLRASR^{71}N**C**SRTENAVC^{81}
CRD2e_K54V	C^{58}-C^{73}	PCP^{41}PGTYIHLNG^{51}LSVCLQ**C**QMC^{61}DPAMGLRASR^{71}N**C**SRTENAVC^{81}
CRD2e_K54Y	C^{58}-C^{73}	PCP^{41}PGTYIHLNG^{51}LSYCLQ**C**QMC^{61}DPAMGLRASR^{71}N**C**SRTENAVC^{81}
CRD2e_K54E_D62A	C^{58}-C^{73}	PCP^{41}PGTYIHLNG^{51}LSECLQ**C**QMC^{61}APAMGLRASR^{71}N**C**SRTENAVC^{81}
CRD2e_K54E_D62K	C^{58}-C^{73}	PCP^{41}PGTYIHLNG^{51}LSECLQ**C**QMC^{61}KPAMGLRASR^{71}N**C**SRTENAVC^{81}
CRD2e_K54E_D62L	C^{58}-C^{73}	PCP^{41}PGTYIHLNG^{51}LSECLQ**C**QMC^{61}LPAMGLRASR^{71}N**C**SRTENAVC^{81}
CRD2e_K54E_D62S	C^{58}-C^{73}	PCP^{41}PGTYIHLNG^{51}LSECLQ**C**QMC^{61}SPAMGLRASR^{71}N**C**SRTENAVC^{81}
CRD2f	C^{61}-C^{81}	PCP^{41}PGTYIHLNG^{51}LSKCLQCQM**C**^{61}DPAMGLRASR^{71}NCSRTENAV**C**^{81}

Table S1: Continued: Names and amino acid sequences of the designed peptides (x denotes point deletion)

CRD2de	C^{40}-C^{55};C^{58}-C^{73}	P**C**P^{41}PGTYIHLNG^{51}LSK**C**LQ**C**QMC^{61}DPAMGLRASR^{71}N**C**SRTENAVC^{81}
CRD2de_K54D	C^{40}-C^{55};C^{58}-C^{73}	P**C**P^{41}PGTYIHLNG^{51}LSD**C**LQ**C**QMC^{61}DPAMGLRASR^{71}N**C**SRTENAVC^{81}
CRD2de_K54E	C^{40}-C^{55};C^{58}-C^{73}	P**C**P^{41}PGTYIHLNG^{51}LSE**C**LQ**C**QMC^{61}DPAMGLRASR^{71}N**C**SRTENAVC^{81}
CRD2de_K54I	C^{40}-C^{55};C^{58}-C^{73}	P**C**P^{41}PGTYIHLNG^{51}LSI**C**LQ**C**QMC^{61}DPAMGLRASR^{71}N**C**SRTENAVC^{81}
CRD2de_K54L	C^{40}-C^{55};C^{58}-C^{73}	P**C**P^{41}PGTYIHLNG^{51}LSL**C**LQ**C**QMC^{61}DPAMGLRASR^{71}N**C**SRTENAVC^{81}
CRD2de_K54S	C^{40}-C^{55};C^{58}-C^{73}	P**C**P^{41}PGTYIHLNG^{51}LSS**C**LQ**C**QMC^{61}DPAMGLRASR^{71}N**C**SRTENAVC^{81}
CRD2df	C^{40}-C^{55};C^{61}-C^{81}	P**C**P^{41}PGTYIHLNG^{51}LSK**C**LQCQM**C**^{61}DPAMGLRASR^{71}NCSRTENAV**C**^{81}
CRD2ef	C^{58}-C^{73};C^{61}-C^{81}	PCP^{41}PGTYIHLNG^{51}LSKCLQ**C**QM**C**^{61}DPAMGLRASR^{71}N**C**SRTENAV**C**^{81}
CRD2def	C^{40}-C^{55};C^{58}-C^{73};C^{61}-C^{81}	P**C**P^{41}PGTYIHLNG^{51}LSK**C**LQ**C**QM**C**^{61}DPAMGLRASR^{71}N**C**SRTENAV**C**^{81}
CRD2(39-73)d	C^{40}-C^{55}	P**C**P^{41}PGTYIHLNG^{51}LSK**C**LQCQMC^{61}DPAMGLRASR^{71}NC^{73}
CRD2(39-73)e	C^{58}-C^{73}	PCP^{41}PGTYIHLNG^{51}LSKCLQ**C**QMC^{61}DPAMGLRASR^{71}N**C**^{73}
CRD2(39-73)e_P39A	C^{58}-C^{73}	ACP^{41}PGTYIHLNG^{51}LSKCLQ**C**QMC^{61}DPAMGLRASR^{71}N**C**^{73}
CRD2(39-73)e_P39L	C^{58}-C^{73}	LCP^{41}PGTYIHLNG^{51}LSKCLQ**C**QMC^{61}DPAMGLRASR^{71}N**C**^{73}
CRD2(39-73)e_P39I	C^{58}-C^{73}	ICP^{41}PGTYIHLNG^{51}LSKCLQ**C**QMC^{61}DPAMGLRASR^{71}N**C**^{73}
CRD2(39-73)e_P39V	C^{58}-C^{73}	VCP^{41}PGTYIHLNG^{51}LSKCLQ**C**QMC^{61}DPAMGLRASR^{71}N**C**^{73}
CRD2(39-73)e_P39W	C^{58}-C^{73}	WCP^{41}PGTYIHLNG^{51}LSKCLQ**C**QMC^{61}DPAMGLRASR^{71}N**C**^{73}
CRD2(39-73)e_P39x	C^{57}-C^{72}	CP^{40}PGTYIHLNG^{50}LSKCLQ**C**QMC^{60}DPAMGLRASR^{70}N**C**^{72}
CRD2(39-73)e_K54E	C^{58}-C^{73}	PCP^{41}PGTYIHLNG^{51}LSECLQ**C**QMC^{61}DPAMGLRASR^{71}N**C**^{73}
CRD2(39-73)e_C55A	C^{58}-C^{73}	PCP^{41}PGTYIHLNG^{51}LSKALQ**C**QMC^{61}DPAMGLRASR^{71}N**C**^{73}
CRD2(39-73)e_C55V	C^{58}-C^{73}	PCP^{41}PGTYIHLNG^{51}LSKVLQ**C**QMC^{61}DPAMGLRASR^{71}N**C**^{73}
CRD2(39-73)e_C61S	C^{58}-C^{73}	PCP^{41}PGTYIHLNG^{51}LSKCLQ**C**QMS^{61}DPAMGLRASR^{71}N**C**^{73}
CRD2(39-73)e_N72A	C^{58}-C^{73}	PCP^{41}PGTYIHLNG^{51}LSKCLQ**C**QMC^{61}DPAMGLRASR^{71}A**C**^{73}
CRD2(39-73)e_N72G	C^{58}-C^{73}	PCP^{41}PGTYIHLNG^{51}LSKCLQ**C**QMC^{61}DPAMGLRASR^{71}G**C**^{73}
CRD2(39-73)de	C^{40}-C^{55};C^{58}-C^{73}	P**C**P^{41}PGTYIHLNG^{51}LSK**C**LQ**C**QMC^{61}DPAMGLRASR^{71}N**C**^{73}
CRD2(39-73)no	without	PCP^{41}PGTYIHLNG^{51}LSKCLQCQMS^{61}DPAMGLRASR^{71}NC^{73}
CRD3no	without	GCSPQHFCIV^{91}QDGDHCAACR^{101}AYA
CRD3g	C^{83}-C^{100}	G**C**SPQHFCIV^{91}QDGDHCAA**C**R^{101}AYA
CRD3h	C^{89}-C^{97}	GCSPQHF**C**IV^{91}QDGDH**C**AACR^{101}AYA
CRD3gh	C^{83}-C^{100};C^{89}-C^{97}	G**C**SPQHF**C**IV^{91}QDGDH**C**AA**C**R^{101}AYA
CRD3(89-97)h	C^{89}-C^{97}	**C**IV^{91}QDGDH**C**
CRD3(89-97)no	without	CIV^{91}QDGDHC

Table S2: Details of the simulations performed in the project. cMD stands for conventional Molecular Dynamics simulations, SMD for Steered Molecular Dynamics simulations with 0.05 and 0.01 m/s for fast and slow pulling, respectively, and US for Umbrella Sampling. All designed variants of HVEM-based molecules are treated as possible inhibitors, while complete HVEM simulations were run for comparison only.

**Description**	**No. of Molecules**	**Number and Length of Trajectories**	**Total Time [µs]**
cMD of inhibitors with LIGHT	65	3 × 200ns	37.8
cMD of HVEM-LIGHT complex	2	3 × 200ns	1.2
cMD of top inhibitors with LIGHT	4	3 × 800ns (continuation from 200ns)	9.6
cMD of unbound inhibitors	29	3 × 1000ns	87
cMD of complete unbound HVEM	2	3 × 1000ns	6
SMD_{fast} of inhibitors with LIGHT	4	25 × 80ns	8
SMD_{slow} of inhibitors with LIGHT	4	10 × 250ns	10
US of inhibitors with LIGHT	4	44 × 100ns	17.6
			**177.2**

Table S3: Estimated secondary structure content

	Estimated secondary structure content (%)															
	α-Helix				β-sheet				Turn				Coil			
**Peptide**	DSSP	STRIDE	KAKSI	CD	DSSP	STRIDE	KAKSI	CD	DSSP	STRIDE	KAKSI	CD	DSSP	STRIDE	KAKSI	CD
HVEM-CRD1no	0.60	0.93			35.33	30.20			27.48	36.06			36.58	32.62		
CRD1no	4.49	8.89	5.50		10.26	12.47	9.20		34.32	38.20			50.94	45.55	85.29	
CRD1a	4.58	10.82	7.77		21.78	21.94	13.41		30.28	35.22			43.36	37.60	78.82	
CRD1b	1.42	6.24	0.31		20.55	16.22	8.23		32.63	40.93			45.40	41.16	91.46	
CRD1c	2.45	5.64	0.35		12.84	7.17	2.61		33.80	43.39			50.91	49.60	97.04	
CRD1ab	3.02	6.18	0.73		23.15	27.39	17.83		30.98	34.84			42.85	36.36	81.44	
CRD1ac	1.90	6.49	1.87		26.08	24.30	13.81		31.42	42.30			40.59	30.75	84.32	
CRD1bc	0.54	3.39	0.57		26.70	22.26	13.17		27.87	41.68			44.89	33.75	86.26	
CRD1abc	0.02	3.07	0.00		26.65	24.04	15.18		27.66	42.82			45.66	32.84	84.82	
HVEM-CRD1abc	0.00	0.00			39.97	37.22			23.23	35.38			36.80	27.14		
HVEM-CRD2no	1.87	3.07			12.39	10.89			29.86	31.55			55.87	52.28		
CRD2no	5.22	8.13	6.46	3.9	10.44	4.48	0.34	28	32.44	39.50		17.6	51.90	47.57	93.20	50.5
CRD2d	4.14	4.98	2.87	10.9	12.86	10.21	3.33	26.4	33.71	37.72		15.4	49.30	46.38	93.81	47.3
CRD2e	2.58	0.89	0.09	13.3	20.28	17.48	16.21	18.8	28.65	37.92		15	48.48	40.24	83.70	52.9
CRD2e_K54E	3.23	2.80	0.53	14.1	19.90	17.66	14.07	21.6	27.61	30.97		15.1	49.26	46.70	85.40	49.2
CRD2f	2.84	3.44	3.76	7.9	13.28	13.71	11.27	23.3	31.97	40.68		17.3	51.92	39.71	84.97	51.5
CRD2de	1.56	0.32	0.02	3.2	19.48	18.07	8.88	32.1	30.96	37.65		17.4	48.00	42.97	91.10	47.3
CRD2df	1.38	2.95	0.00	1.2	20.84	20.34	7.44	39.5	34.66	42.10		15.1	43.12	33.37	92.56	44.2
CRD2ef	1.32	1.08	0.10	2.5	19.63	19.77	16.11	33.4	28.15	32.18		17.4	50.90	44.84	83.79	46.7
CRD2def	1.72	0.87	0.00		19.67	18.46	9.88		31.86	39.83			46.75	38.18	90.12	
CRD2(39-73)e	6.61	9.42	11.10		9.09	2.13	5.67		32.29	28.69			52.10	88.44	54.37	
HVEM-CRD2def	5.09	6.01			17.45	13.72			24.77	25.31			52.69	50.56		
HVEM-CRD3no	2.31	4.29			20.03	8.55			31.66	37.64			46.00	49.32		
CRD3no	7.56	21.78	13.58		9.44	3.55	0.05		33.10	35.75			49.90	54.49	86.37	
CRD3g	6.31	14.76	0.00		29.96	27.90	16.19		35.73	43.12			28.00	40.41	83.81	
CRD3h	1.45	14.38	1.13		24.19	21.23	0.24		30.29	32.36			44.06	54.11	98.63	
CRD3gh	0.03	14.60	0.00		31.88	38.86	18.22		28.17	35.28			39.92	37.32	81.78	
HVEM-CRD3gh	0.00	0.00			46.69	50.89			24.62	24.24			28.68	24.66		

Table S4: Solvent accessible surfaces area (SASA) [Å^2] obtained for last 20 ns averaged over three trajectories and their standard deviation between trajectories. Δ Sasa hydrophobic indicates the difference in SASA when only hydrophobic residues (GLY,ALA,VAL,LEU,ILE,PRO,PHE,MET,TRP) are taken into consideration

**Name**	**SASA of complex**	**Sum of SASA of components**	**Ratio**	**ΔSASA hydrophobic**
CRD1no	21793 ± 123	22566 ± 259	0.966 ± 0.006	121.89 ± 65.17
CRD1bc	25262 ± 2090	25763 ± 1755	0.980 ± 0.015	85.53 ± 67.89
CRD1(16-38)no	23833 ± 1703	24237 ± 2002	0.984 ± 0.012	16.26 ± 26.36
CRD1-2(16-73)bce	25231 ± 1528	25737 ± 1404	0.980 ± 0.016	108.92 ± 104.12
CRD1-2(16-73)bce_K54E	24095 ± 578	25235 ± 578	0.955 ± 0.024	328.86 ± 192.27
CRD2no	25123 ± 2189	25582 ± 1986	0.982 ± 0.023	56.50 ± 63.87
CRD2no_K54D	24743 ± 1492	25361 ± 1314	0.975 ± 0.018	155.94 ± 200.26
CRD2no_K54I	24971 ± 2016	25456 ± 1592	0.980 ± 0.028	122.83 ± 206.07
CRD2no_K54L	21883 ± 403	23306 ± 445	0.939 ± 0.002	416.64 ± 35.44
CRD2no_K54S	24718 ± 2741	25081 ± 2421	0.984 ± 0.015	80.03 ± 95.60
CRD2no_K54V	24767 ± 2205	25474 ± 1805	0.971 ± 0.021	260.70 ± 223.09
CRD2no_K54Y	26345 ± 2527	26944 ± 2218	0.977 ± 0.016	133.45 ± 89.89
CRD2def	24550 ± 1650	25751 ± 1548	0.953 ± 0.007	317.82 ± 94.98
CRD2d	23337 ± 1531	24674 ± 1702	0.946 ± 0.004	319.52 ± 134.31
CRD2e	24237 ± 2004	24922 ± 1860	0.972 ± 0.024	128.37 ± 6.76
CRD2e_K54D	23486 ± 324	23842 ± 439	0.985 ± 0.007	83.56 ± 46.29
CRD2e_K54E	24103 ± 1751	24662 ± 1499	0.977 ± 0.025	307.50 ± 216.24
CRD2e_K54I	25385 ± 1477	26133 ± 1708	0.972 ± 0.007	320.30 ± 75.40
CRD2e_K54L	22874 ± 1091	24023 ± 554	0.952 ± 0.037	262.49 ± 229.68
CRD2e_K54S	24145 ± 1782	24429 ± 1542	0.988 ± 0.017	85.41 ± 147.93
CRD2e_K54V	23837 ± 1628	24581 ± 1306	0.969 ± 0.016	201.54 ± 121.90
CRD2e_K54Y	23456 ± 3045	24879 ± 2996	0.942 ± 0.011	368.41 ± 90.04
CRD2e_K54E_D62A	22354 ± 595	23329 ± 557	0.959 ± 0.030	229.90 ± 199.63
CRD2e_K54E_D62K	25340 ± 2252	25662 ± 2122	0.987 ± 0.012	66.02 ± 83.04
CRD2e_K54E_D62L	23559 ± 608	24065 ± 750	0.979 ± 0.019	94.17 ± 101.94
CRD2f	24639 ± 1542	25361 ± 1181	0.971 ± 0.021	207.58 ± 179.94
CRD2de	23300 ± 1938	24183 ± 1870	0.963 ± 0.019	210.06 ± 183.06
CRD2de_K54D	22298 ± 949	22956 ± 719	0.971 ± 0.012	148.10 ± 94.97
CRD2de_K54S	21582 ± 744	22400 ± 501	0.963 ± 0.018	164.07 ± 53.50
CRD2de_K54E	22901 ± 464	24198 ± 594	0.947 ± 0.013	341.12 ± 74.60
CRD2de_K54I	22798 ± 376	23902 ± 88	0.954 ± 0.018	313.70 ± 131.19
CRD2df	21272 ± 165	22639 ± 254	0.940 ± 0.004	232.40 ± 154.92
CRD2ef	24146 ± 1967	25097 ± 2028	0.962 ± 0.017	235.08 ± 230.81
CRD2(39-73)no	22343 ± 209	23489 ± 492	0.951 ± 0.012	215.44 ± 39.36
CRD2(39-73)d	26290 ± 1109	26524 ± 981	0.991 ± 0.011	42.95 ± 62.62
CRD2(39-73)e	23765 ± 2148	24621 ± 1688	0.964 ± 0.034	447.85 ± 87.44
CRD2(39-73)e_P39A	23400 ± 2209	23953 ± 2368	0.977 ± 0.017	103.85 ± 92.87
CRD2(39-73)e_P39I	24892 ± 1753	25883 ± 1624	0.961 ± 0.011	183.16 ± 92.87
CRD2(39-73)e_P39L	22699 ± 133	23398 ± 57	0.970 ± 0.004	137.40 ± 30.19
CRD2(39-73)e_P39V	25384 ± 1813	25852 ± 2016	0.983 ± 0.015	74.50 ± 64.61
CRD2(39-73)e_P39W	23523 ± 1889	24169 ± 2042	0.974 ± 0.019	95.89 ± 83.10
CRD2(39-73)e_P39x	24127 ± 1826	24842 ± 1282	0.970 ± 0.024	216.31 ± 261.48
CRD2(39-73)e_C55A	24474 ± 1943	25498 ± 1605	0.959 ± 0.022	268.11 ± 239.23
CRD2(39-73)e_C55V	23251 ± 2839	24533 ± 2756	0.947 ± 0.009	359.40 ± 45.79
CRD2(39-73)e_C61S	23123 ± 365	24232 ± 557	0.954 ± 0.015	326.65 ± 145.24
CRD2(39-73)e_N72A	23896 ± 2472	24779 ± 2119	0.963 ± 0.033	234.76 ± 227.51
CRD2(39-73)e_N72G	22300 ± 454	23785 ± 470	0.938 ± 0.006	374.15 ± 48.42
CRD2(39-73)de	21193 ± 347	22760 ± 453	0.931 ± 0.012	425.29 ± 104.52
CRD3no	24141 ± 849	24272 ± 857	0.995 ± 0.005	34.82 ± 41.84
CRD3(89-97)no	22657 ± 1922	22871 ± 1645	0.990 ± 0.017	0.45 ± 0.54
HVEMno	26750 ± 223	28726 ± 229	0.931 ± 0.011	505.543 ± 166.06
HVEMall	25892 ± 2028	27787 ± 1332	0.931 ± 0.030	496.38 ± 349.50

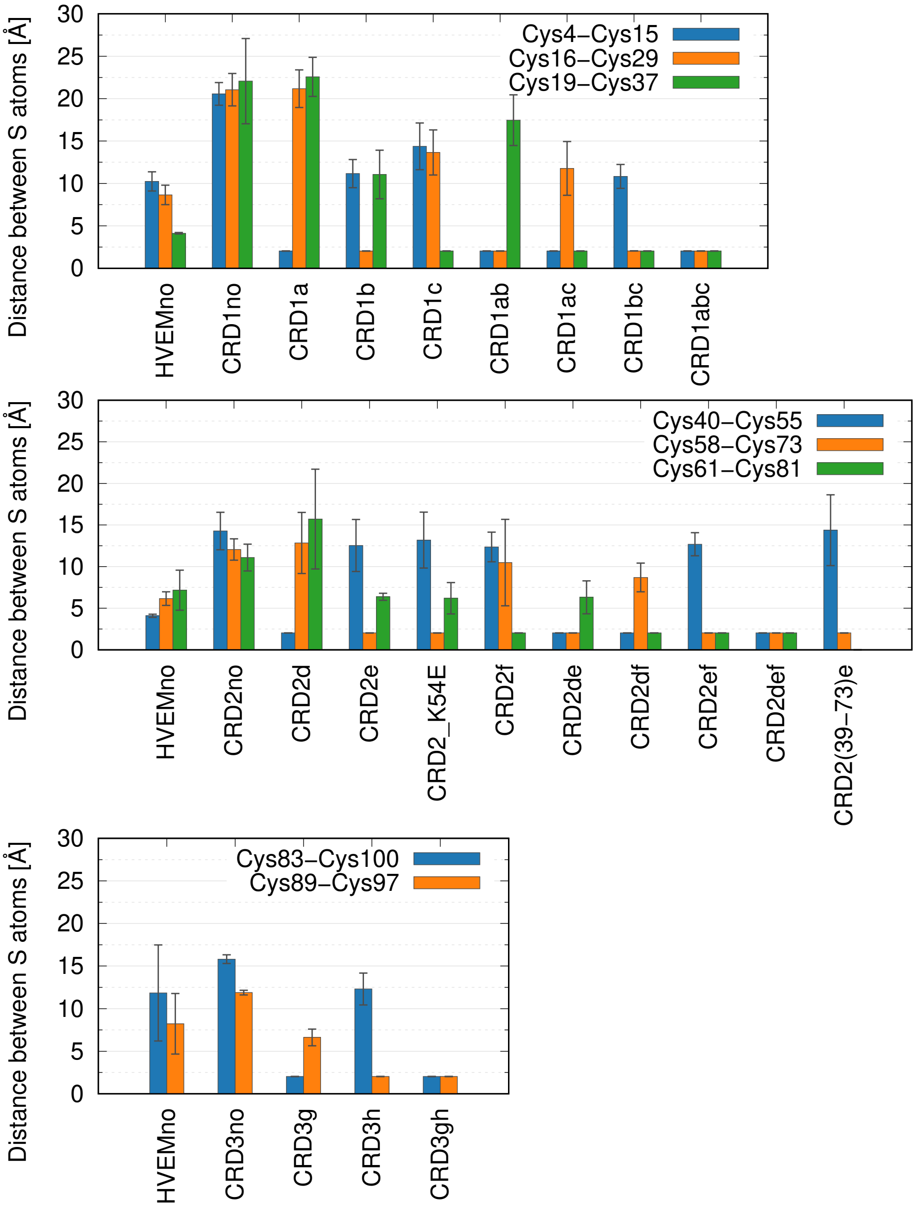

Figure S1: Distance between S atoms in disulfide bonds averaged for 3 trajectories for the second halves of the simulations (500-1000ns) for CRD1, CRD2 and CRD3 without the LIGHT trimer.

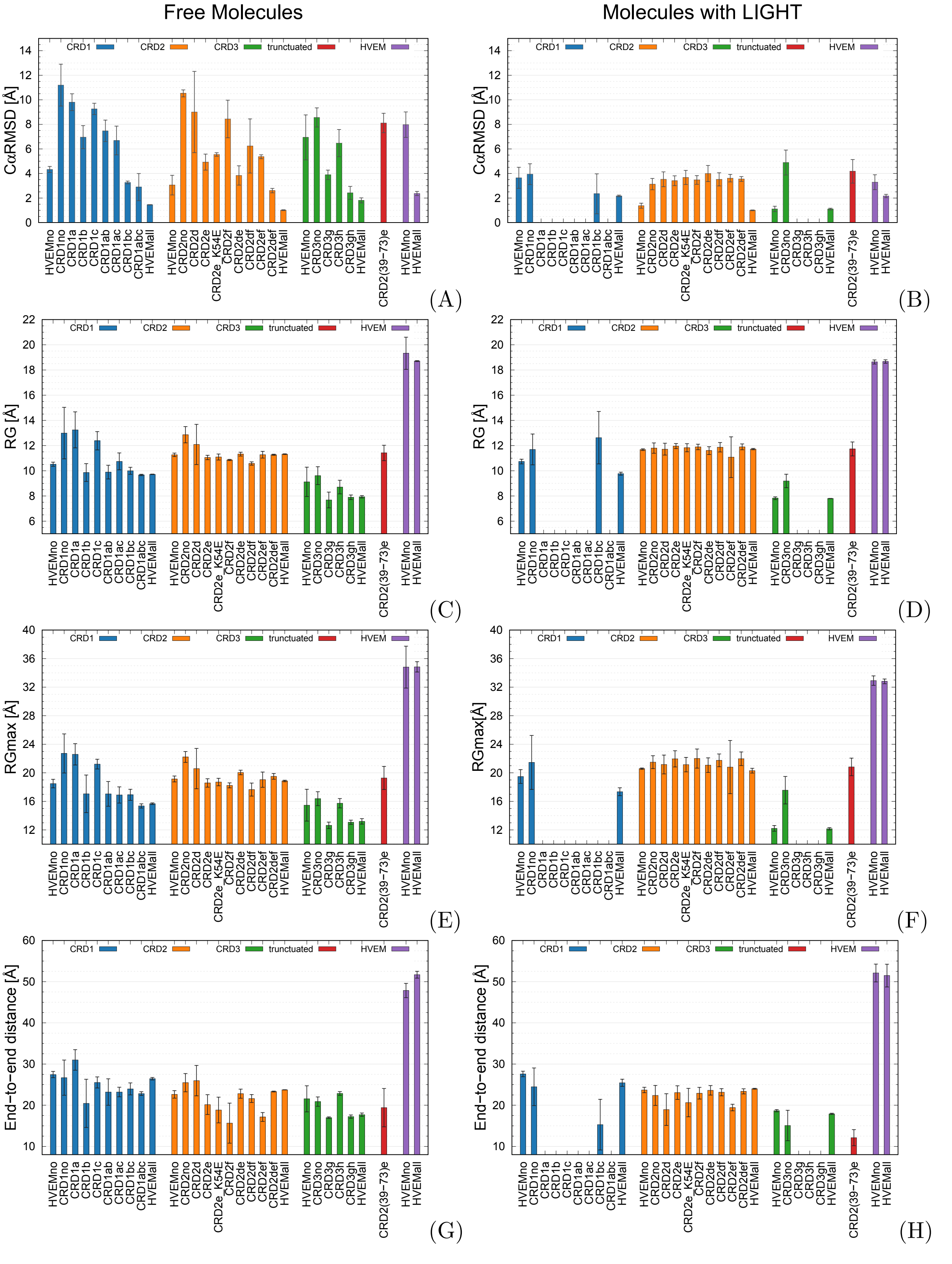

Figure S2: (A) RMSD, (C) RG, (E) RGmax, (G) End-to-end distance calculated from simulations of free domains and (B) RMSD, (D) RG, (F) RGmax and (H) End-to-end distance values calculated from simulations with LIGHT, averaged over the second halves of the 3 trajectories.

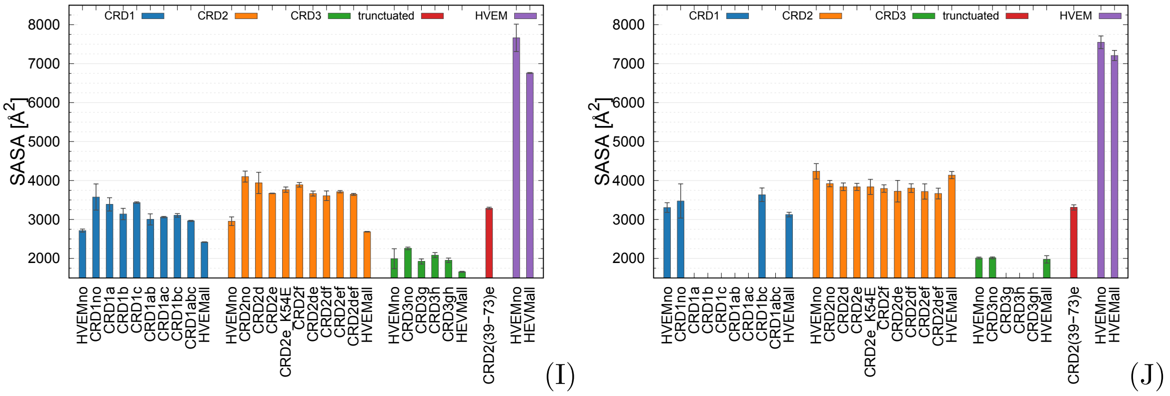

Figure S2: continued: (I) SASA values calculated from simulation of free domains and (J) theoretical SASA values calculated from simulation with the LIGHT trimer (the LIGHT trimer was excluded in this analysis, therefore, the whole HVEM variant surface was computed) averaged over the second halves of the 3 trajectories.

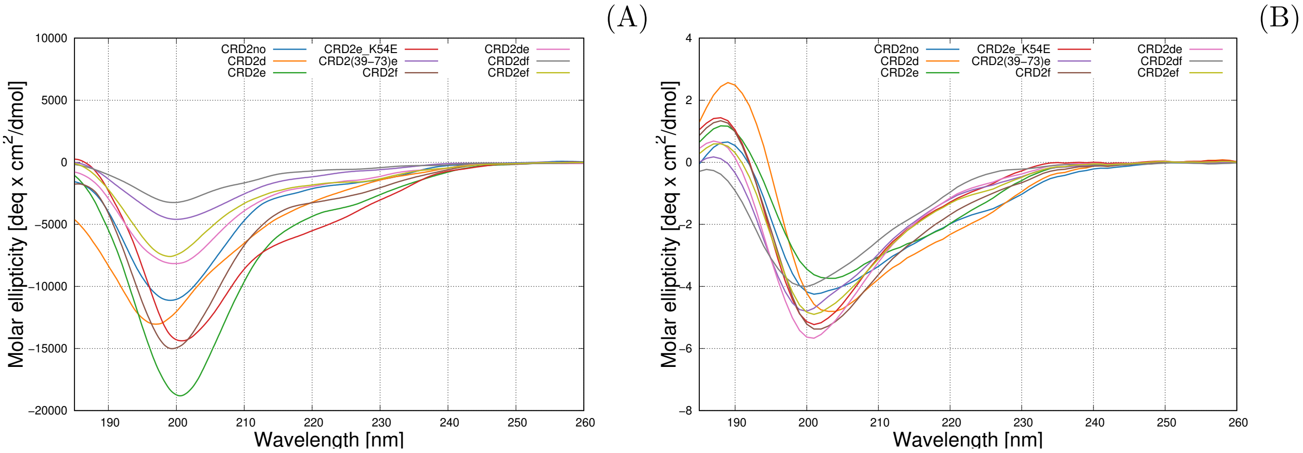

Figure S3: Experimental (A) and simulated (B) CD spectra of the selected designed peptides.

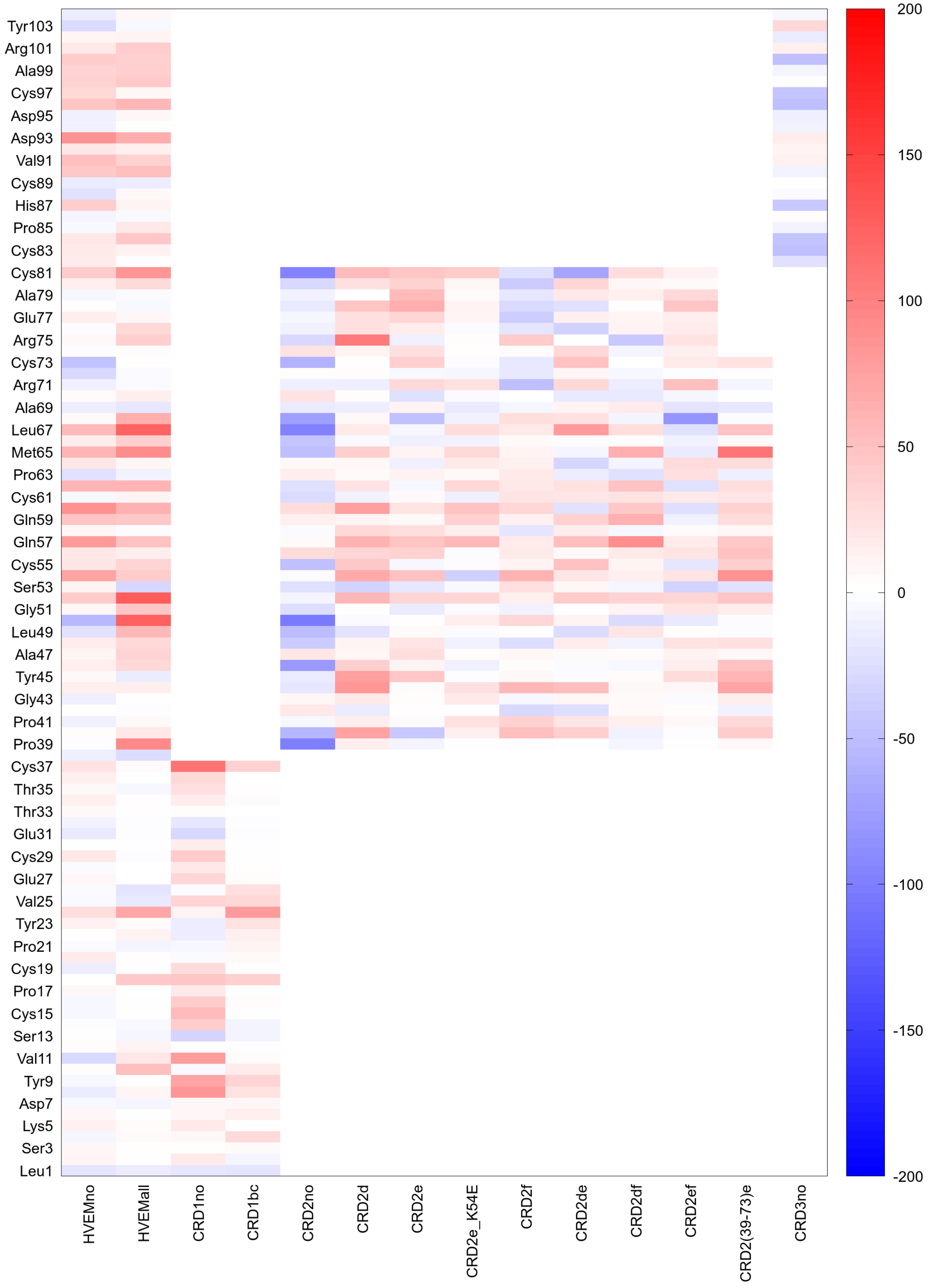

Figure S4: Heatmap of per-residue $\Delta SASA = SASA_{\text{free}} - SASA_{\text{complex}}$ (right-hand bar [Å^2]) calculated for all combinations of disulfide bonds present and absent in the simulations for the full HVEM molecule, its domains (CRD1-3) and it variant, and CRD2e_K54E, and fragment (CRD(39-73)e), averaged over the second halves of the 3 trajectories.

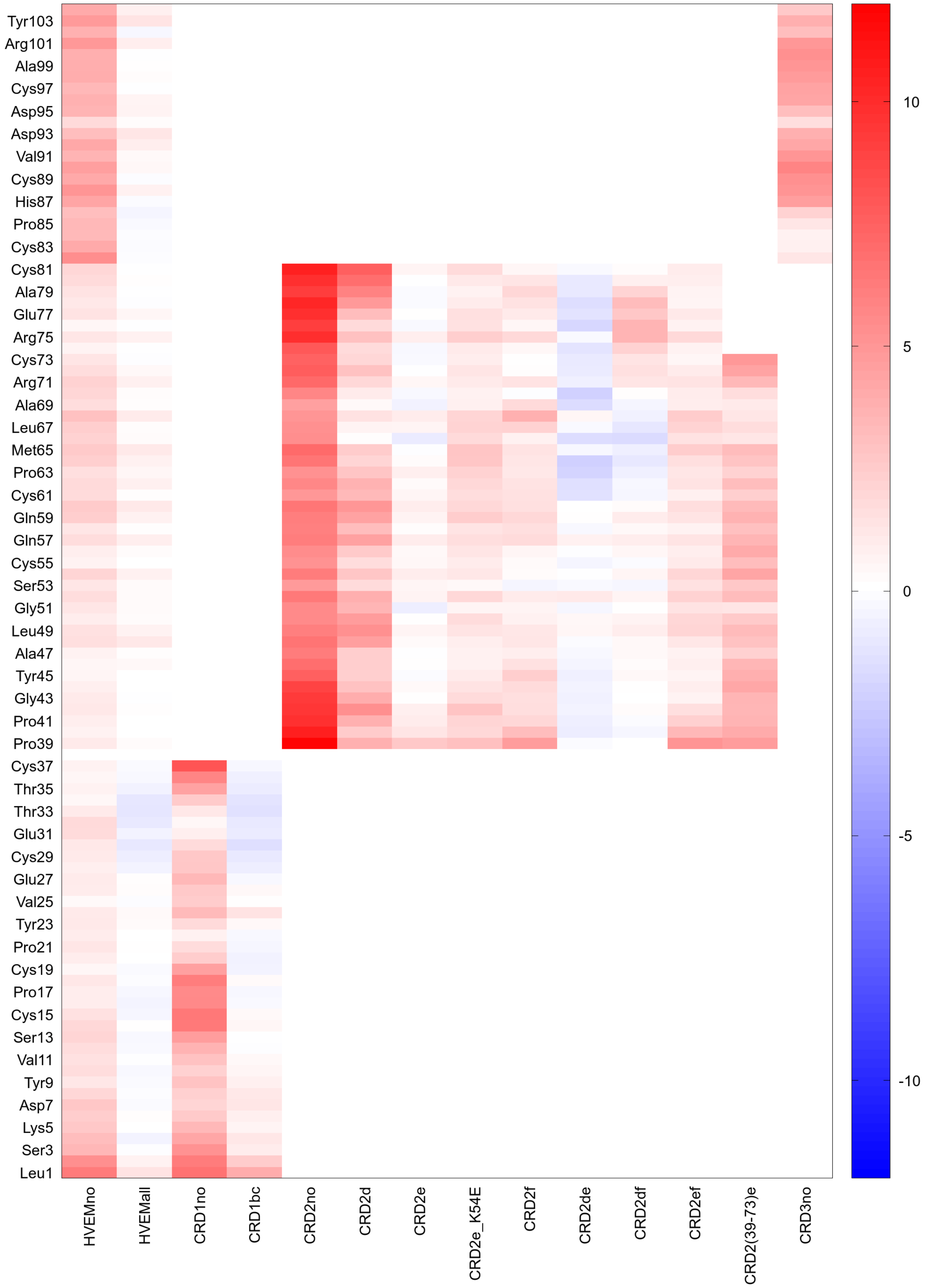

Figure S5: Heatmap of per-residue $\Delta RMSF = RMSF_{\text{free}} - RMSF_{\text{complex}}$ (right-hand bar [Å]) calculated for HVEM with and without disulfide bonds, and its selected variants forming stable complexes with LIGHT, averaged over the second halves of the 3 trajectories.

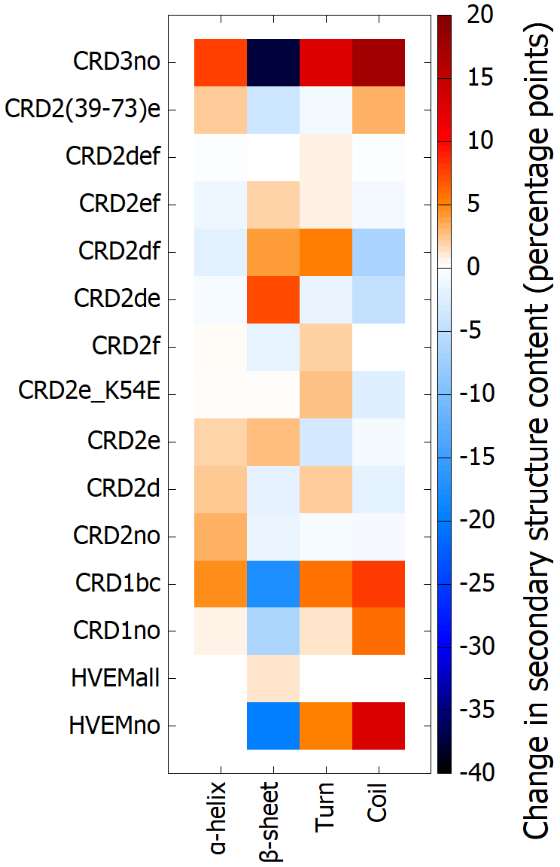

Figure S6: Heatmap of the change in the content of secondary structures expressed in percentage points calculated for HVEM with and without disulfide bonds, and its selected variants forming stable complexes with LIGHT, averaged over the second halves of the 3 trajectories

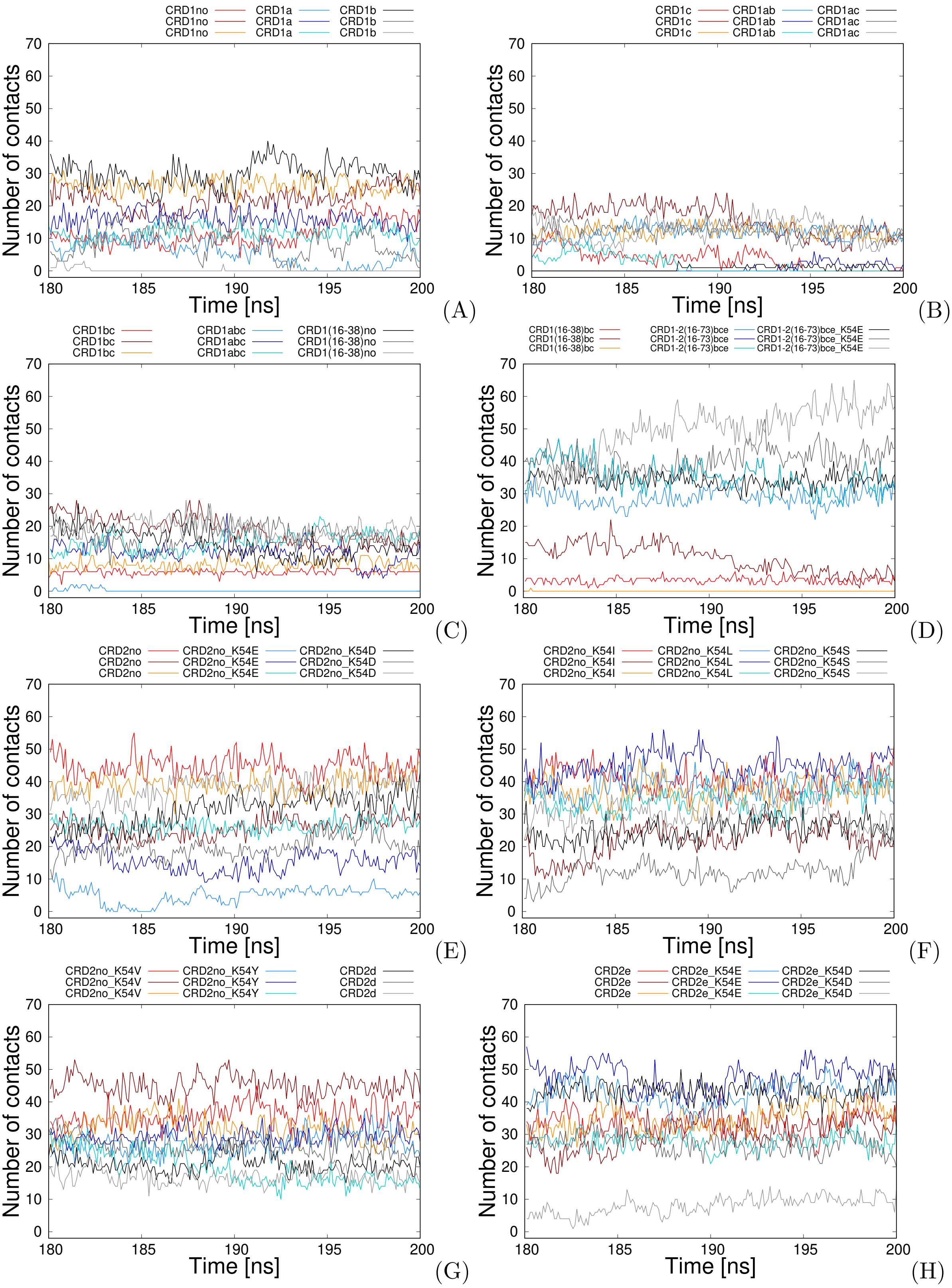

Figure S7: Number of native contacts between HVEM variant and LIGHT trimer as a function of time for last 20ns for (A): CRD1no (red), CRD1a (blue), and CRD1b (grey); (B): CRD1c (red), CRD1ab (blue), and CRD1ac (grey); (C): CRD1bc (red), CRD1abc (blue), and CRD1(16-38)no (grey); (D): CRD1(16-38)bc (red), CRD1-2(16-73)bce (blue), and CRD1-2(16-73)bce_K54E (grey) (E): CRD2no (red) CRD2no_K54E (blue), CRD2no_K54D (grey); (F): CRD2no_K54I (red); (F): CRD2no_K54L (blue), and CRD2no_K54S (grey); (G): CRD2no_K54V (red) CRD2no_K54Y (blue), and CRD2d (grey); (H): CRD2e (red), CRD2e_K54E (blue), and CRD2e_K54D (grey). Different trajectories are depicted as various color tone.

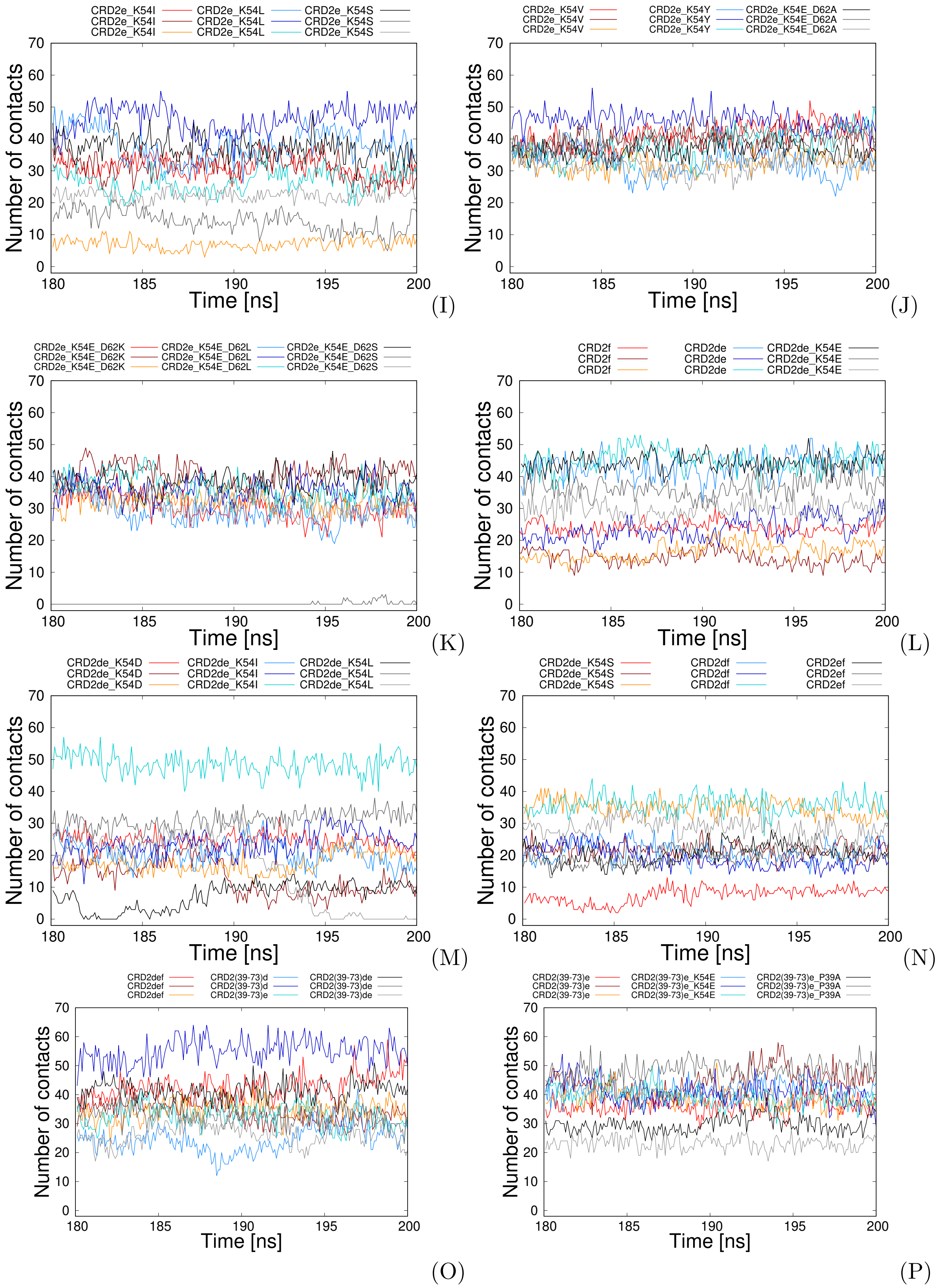

Figure S7: continued: (I): CRD2e_K54I (red), CRD2e_K54L (blue), and CRD2e_K54S (grey); (J) CRD2e_K54V (red), CRD2e_K54Y (blue), and CRD2e_K54E_D62A (grey); (K): CRD2e_K54E_D62K (red), CRD2e_K54E_D62L (blue), and CRD2e_K54E_D62S (grey); (L): CRD2f (red); CRD2de (blue) and CRD2de_K54E (grey); (M): CRD2de_K54D (red), CRD2de_K54I (blue), and CRD2de_K54L (grey); (N): CRD2de_K54S (red), CRD2df (blue), and CRD2ef (grey); (O): CRD2def (red), CRD2(39-73)d (blue), and CRD2(39-73)de (grey); (P): CRD2(39-73)e (red), CRD2(39-73)e_K54E(blue), and CRD2(39-73)e_P39A (grey). Different trajectories are depicted as various color tone.

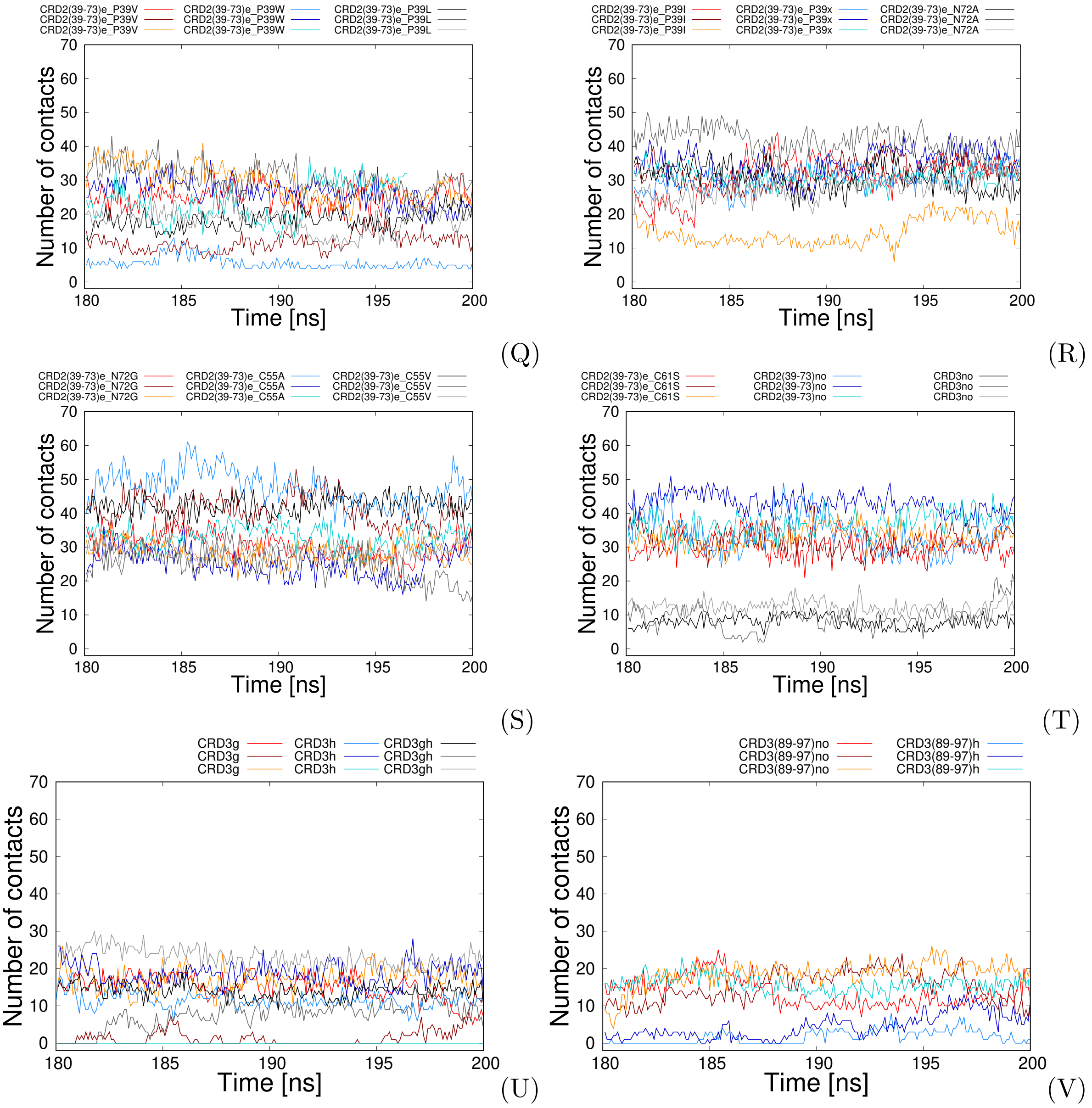

Figure S7: continued (Q): CRD2(39-73)e_P39V(red), CRD2(39-73)e_P39W (blue), and CRD2(39-73)e_P39L (grey); (R): CRD2(39-73)e_P39I (red), CRD2(39-73)e_P39x(blue), and CRD2(39-73)e_N72A (grey); (S): CRD2(39-73)e_N72G(red), CRD2(39-73)e_C55A (blue), and CRD2(39-73)e_C55V (grey); (T): CRD2(39-73)e_C61S (red), CRD2(39-73)no (blue) and CRD3no (grey); (U): CRD3g (red), CRD3h (blue) and CRD3gh(grey); (V): CRD3(89-97)no (red), and CRD3(89-97)h (blue) . Different trajectories are depicted as various color tone.

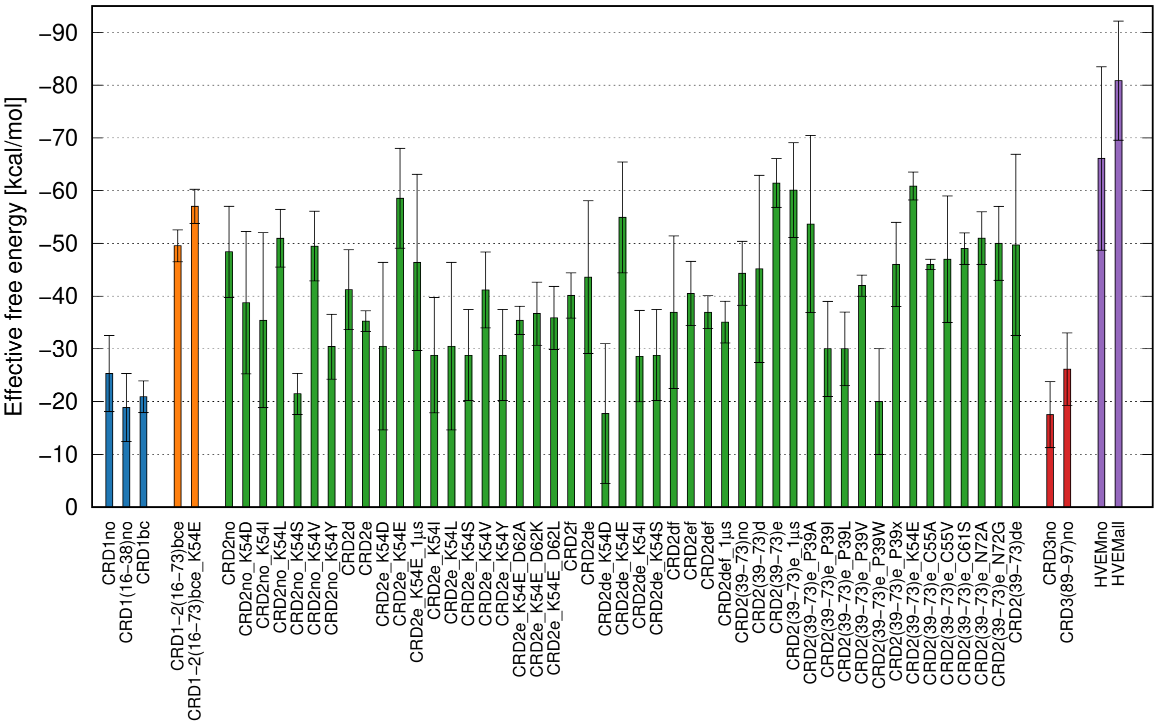

Figure S8: Bar plot of the effective free energies of the various stable variants of the HVEM molecule interacting with the LIGHT trimer.

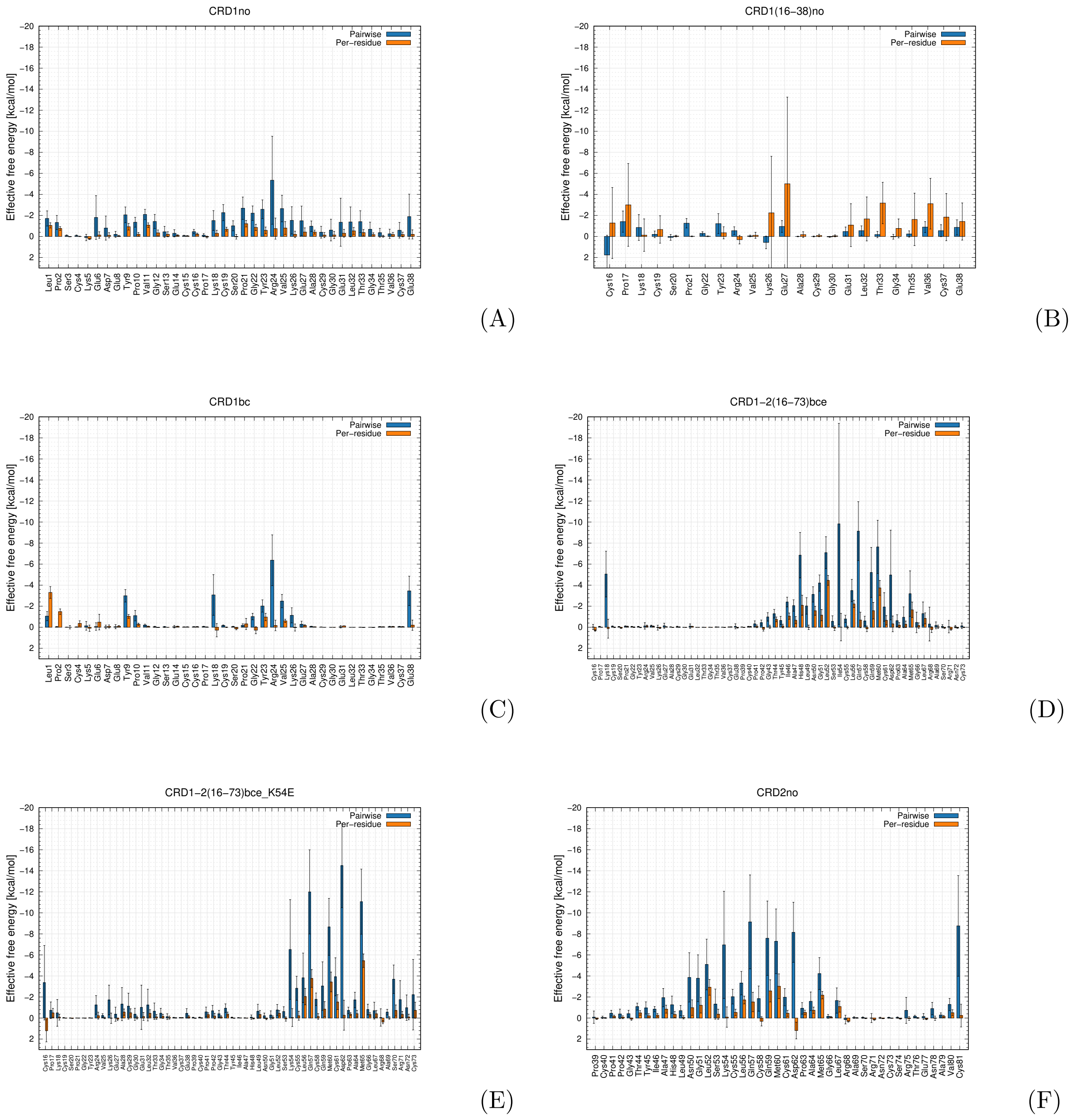

Figure S9: MM-GBSA effective binding energy decomposition results for (A) CRD1no, (B) CRD1(16-38)no, (C) CRD1bc, (D) CRD1-2(16-73)bce, (E) CRD1-2(16-73)bce_K54E, (F) CRD2no variants

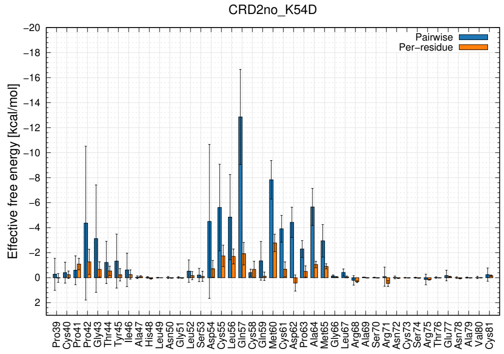

(G)

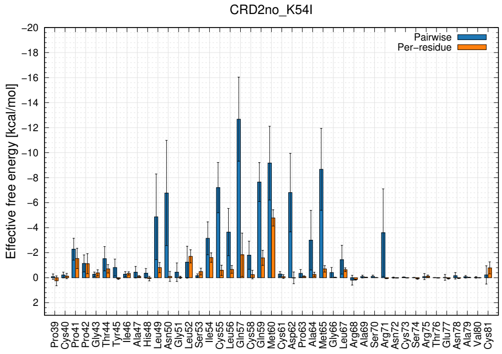

(H)

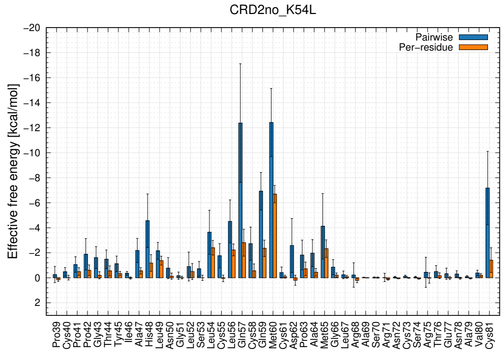

(I)

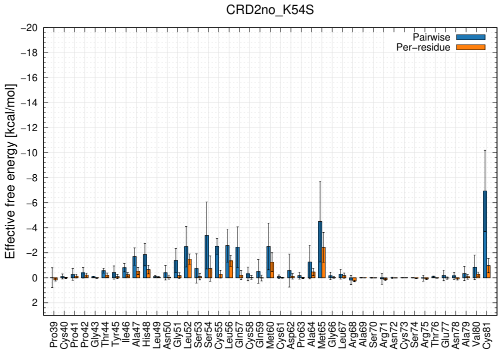

(J)

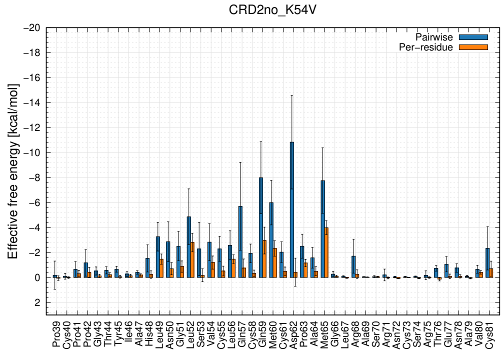

(K)

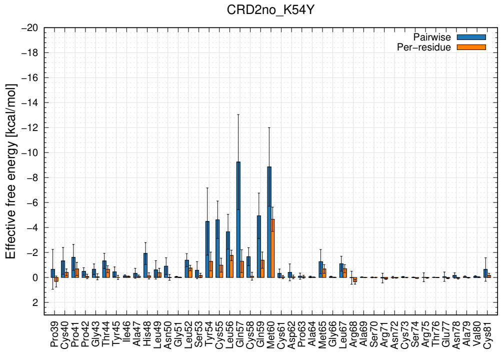

(L)

Figure S9: continued: MM-GBSA effective binding energy decomposition results for (G) CRD2no_K54D, (H) CRD2no_K54I, (I) CRD2no_K54L, (J) CRD2no_K54S, (K) CRD2no_K54V, (L) CRD2no_K54Y variants

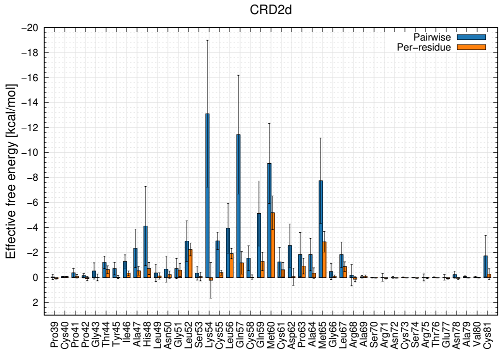

(M)

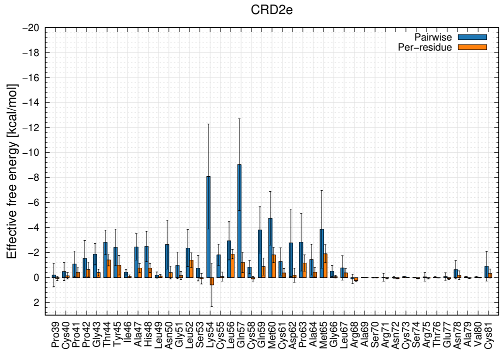

(N)

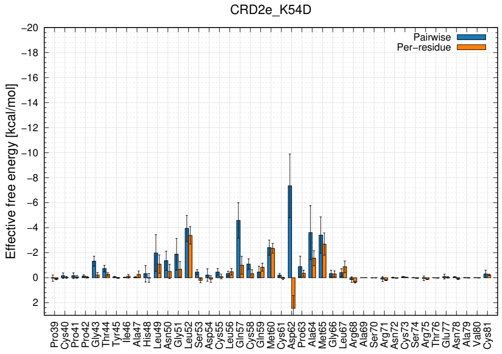

(O)

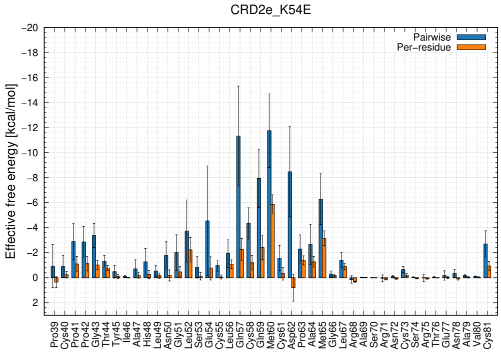

(P)

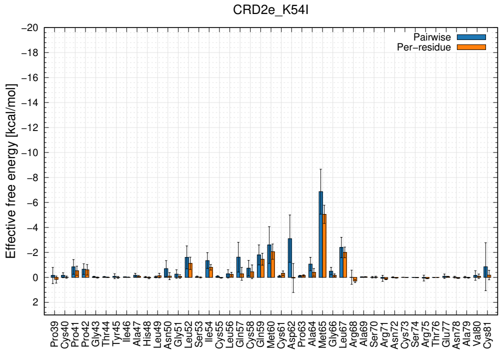

(Q)

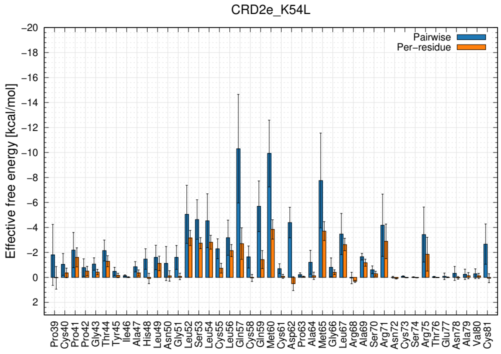

(R)

Figure S9: continued: MM-GBSA effective binding energy decomposition results for: (M) CRD2d, (N) CRD2e, (O) CRD2e_K54D, (P) CRD2e_K54E, (Q) CRD2e_K54I, (R) CRD2e_K54L variants

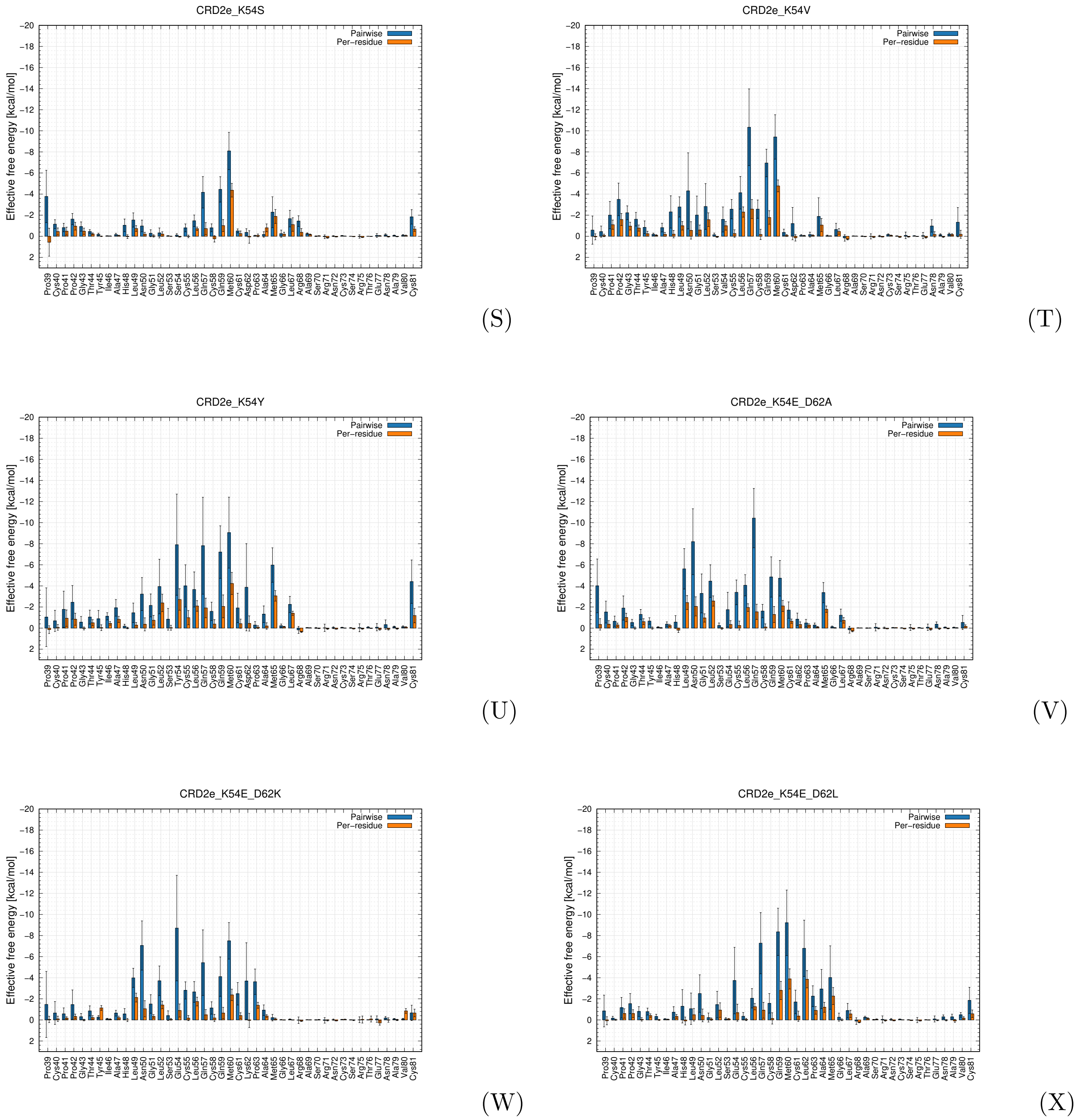

Figure S9: continued: MM-GBSA effective binding energy decomposition results for: (S) CRD2e_K54S, (T) CRD2e_K54V, (U) CRD2e_K54Y, (V) CRD2e_K54E_D62A, (W) CRD2e_K54E_D62K, (X) CRD2e_K54E_D62L variants

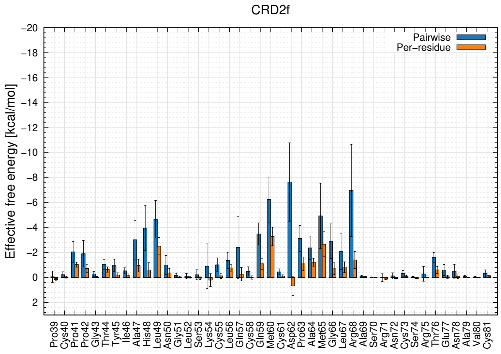

(Y)

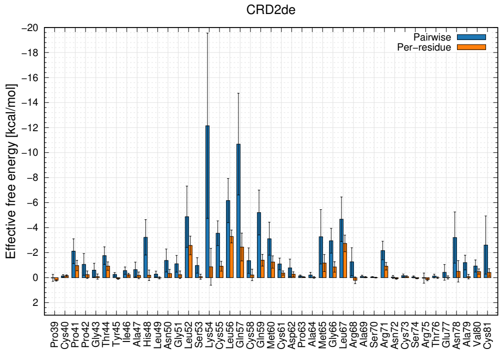

(Z)

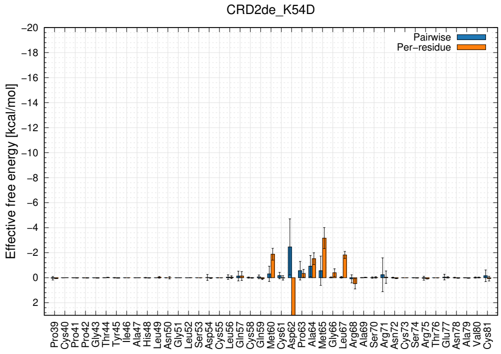

(AA)

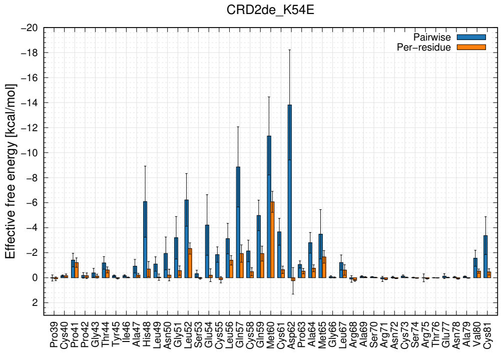

(AB)

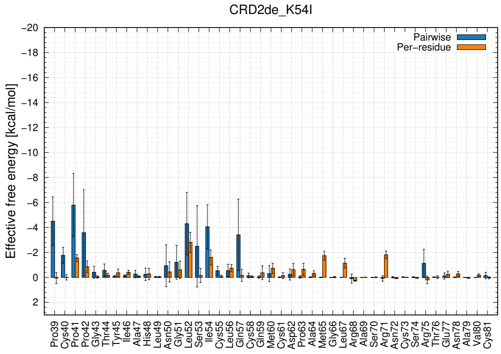

(AC)

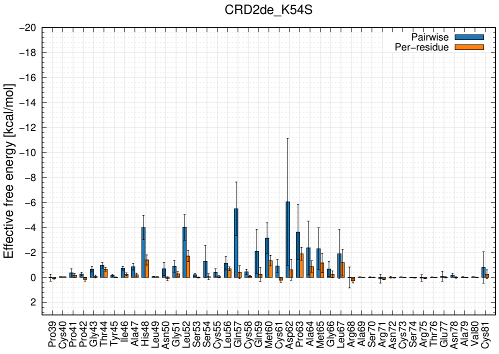

(AD)

Figure S9: continued: MM-GBSA effective binding energy decomposition results for: (Y) CRD2f, (Z) CRD2de, (AA) CRD2de_K54D, (AB) CRD2de_K54E, (AC) CRD2de_K54I, (AD) CRD2de_K54S variants

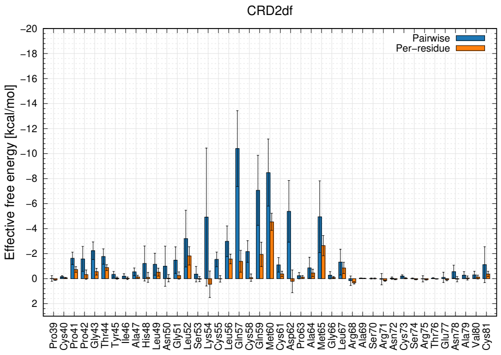

(AE)

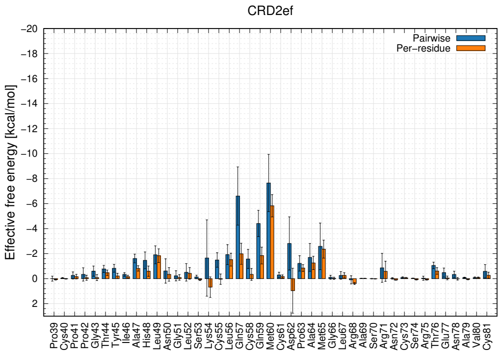

(AF)

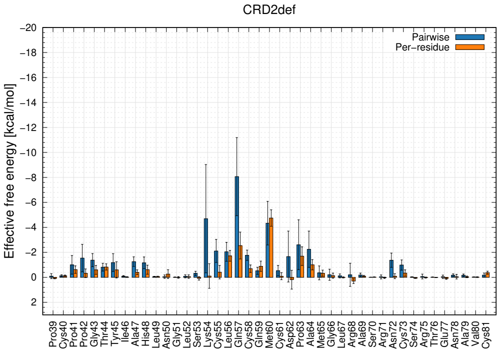

(AG)

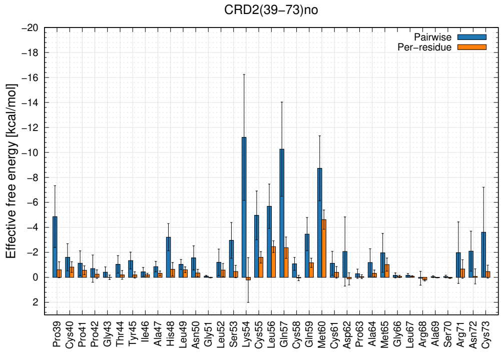

(AH)

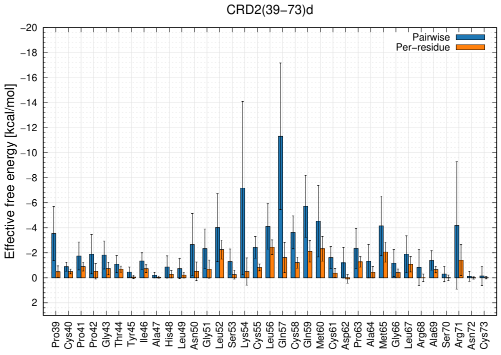

(AI)

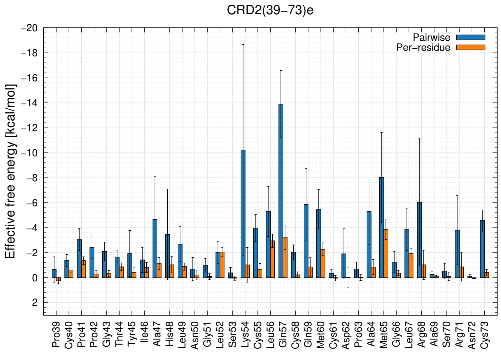

(AJ)

Figure S9: continued: MM-GBSA effective binding energy decomposition results for: (AE) CRD2df, (AF) CRD2ef, (AG) CRD2def, (AH) CRD2(39-73)no, (AI) CRD2(39-73)d, (AJ) CRD2(39-73)e variants

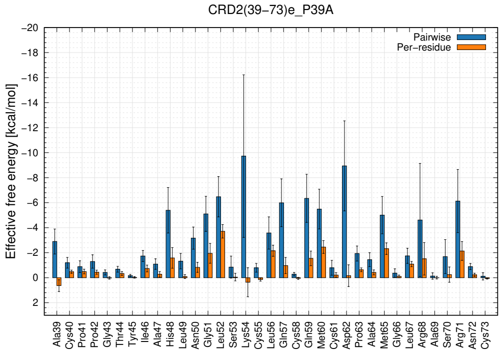

(AK)

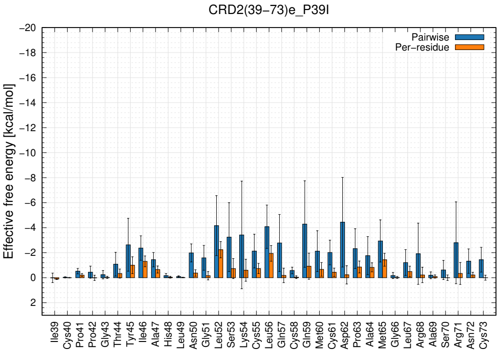

(AL)

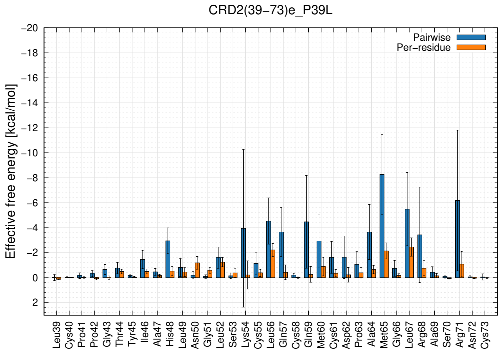

(AM)

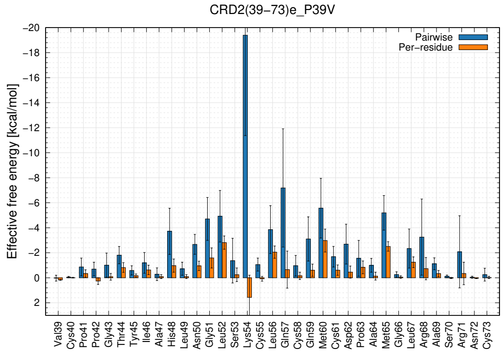

(AN)

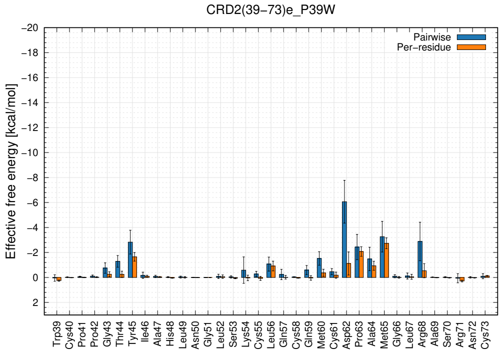

(AO)

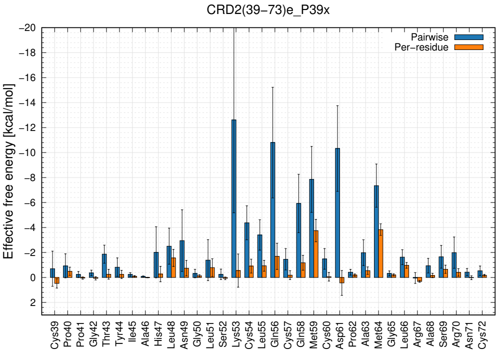

(AP)

Figure S9: continued: MM-GBSA effective binding energy decomposition results for: (AK) CRD2(39-73)e_P39A, (AL) CRD2(39-73)e_P39I, (AM) CRD2(39-73)e_P39L, (AN) CRD2(39-73)e_P39V, (AO) CRD2(39-73)e_P39W, (AP) CRD2(39-73)e_P39x variants

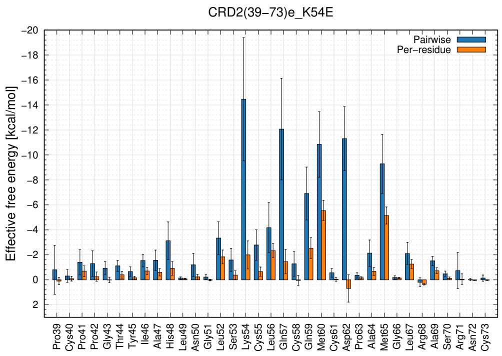

(AQ)

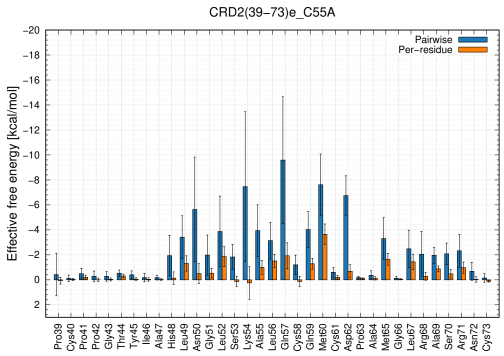

(AR)

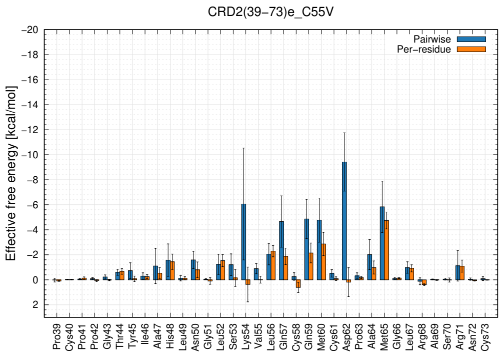

(AS)

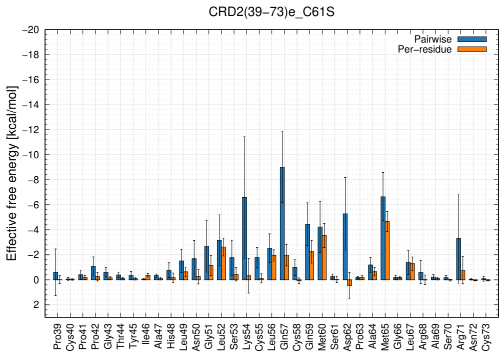

(AT)

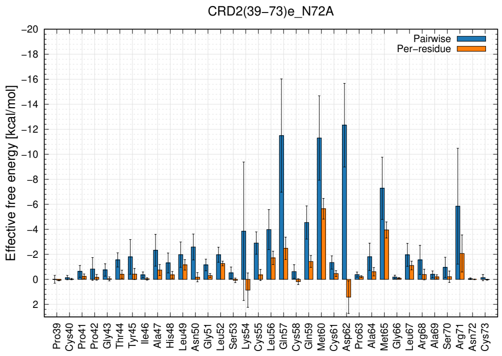

(AU)

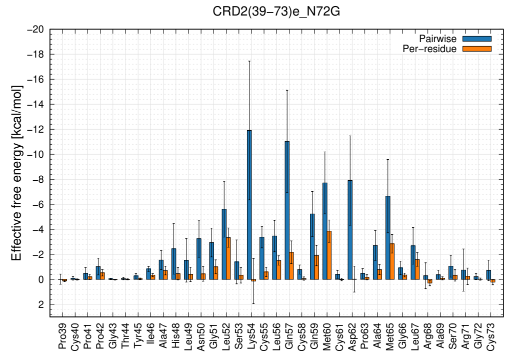

(AV)

Figure S9: continued: MM-GBSA effective binding energy decomposition results for: (AQ) CRD2(39-73)e_K54E, (AR) CRD2(39-73)e_C55A, (AS) CRD2(39-73)e_C55V, (AT) CRD2(39-73)e_C61S, (AU) CRD2(39-73)e_N72A, (AV) CRD2(39-73)e_N72G variants

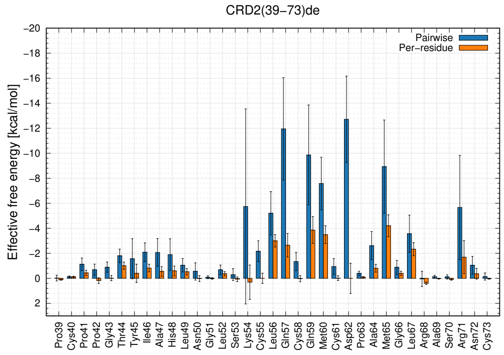

(AW)

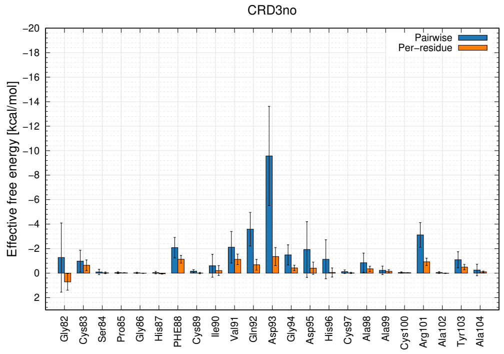

(AX)

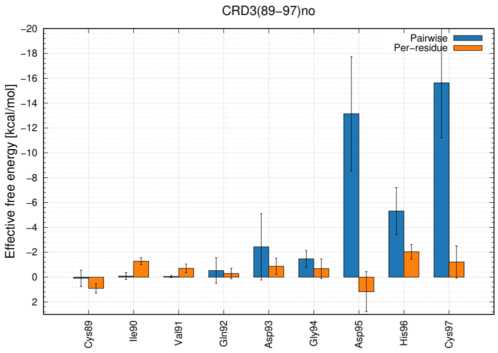

(AY)

Figure S9: continued: MM-GBSA effective binding energy decomposition results for: (AW) CRD2(39-73)de, (AX) CRD3no, (AY) CRD3(39-73)no

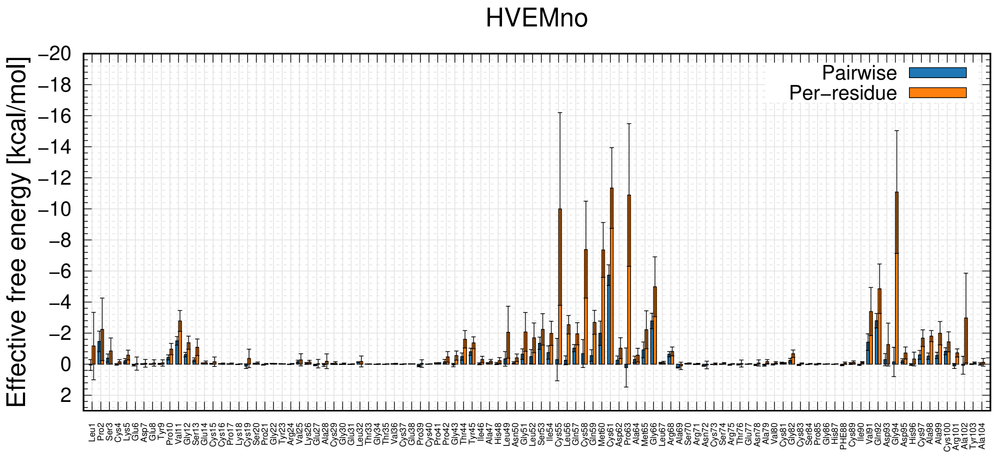

(AZ)

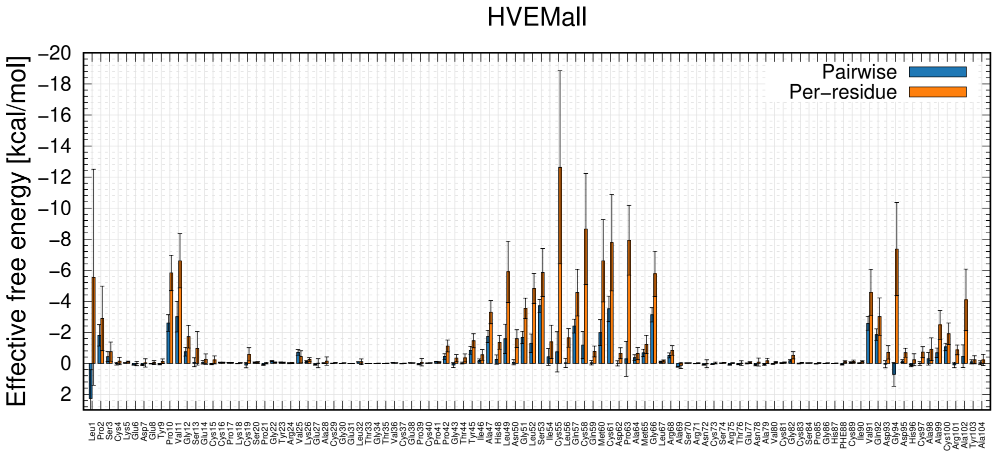

(AAA)

Figure S9: continued: MM-GBSA effective binding energy decomposition results for: (AZ) HVEMno variant, (AAA) HVEMall

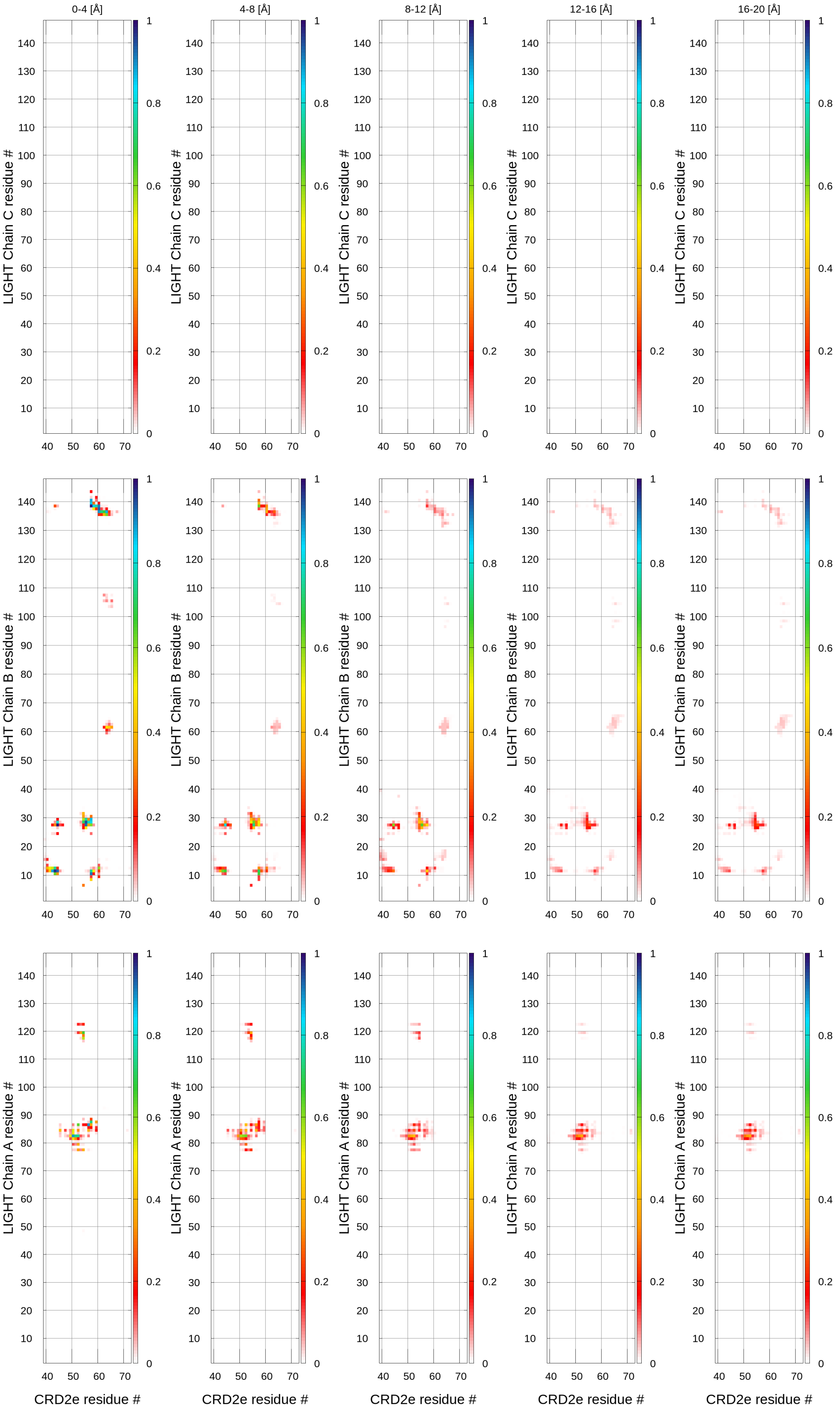

Figure S10: Contact heatmap averaged over 25 SMD simulations for HVEM variant CRD2e with the LIGHT trimer. The values are normalized in the range of increasing extension (relative difference of the distance between centers of mass of HVEM variant and LIGHT trimer) averaged for 4 Å.

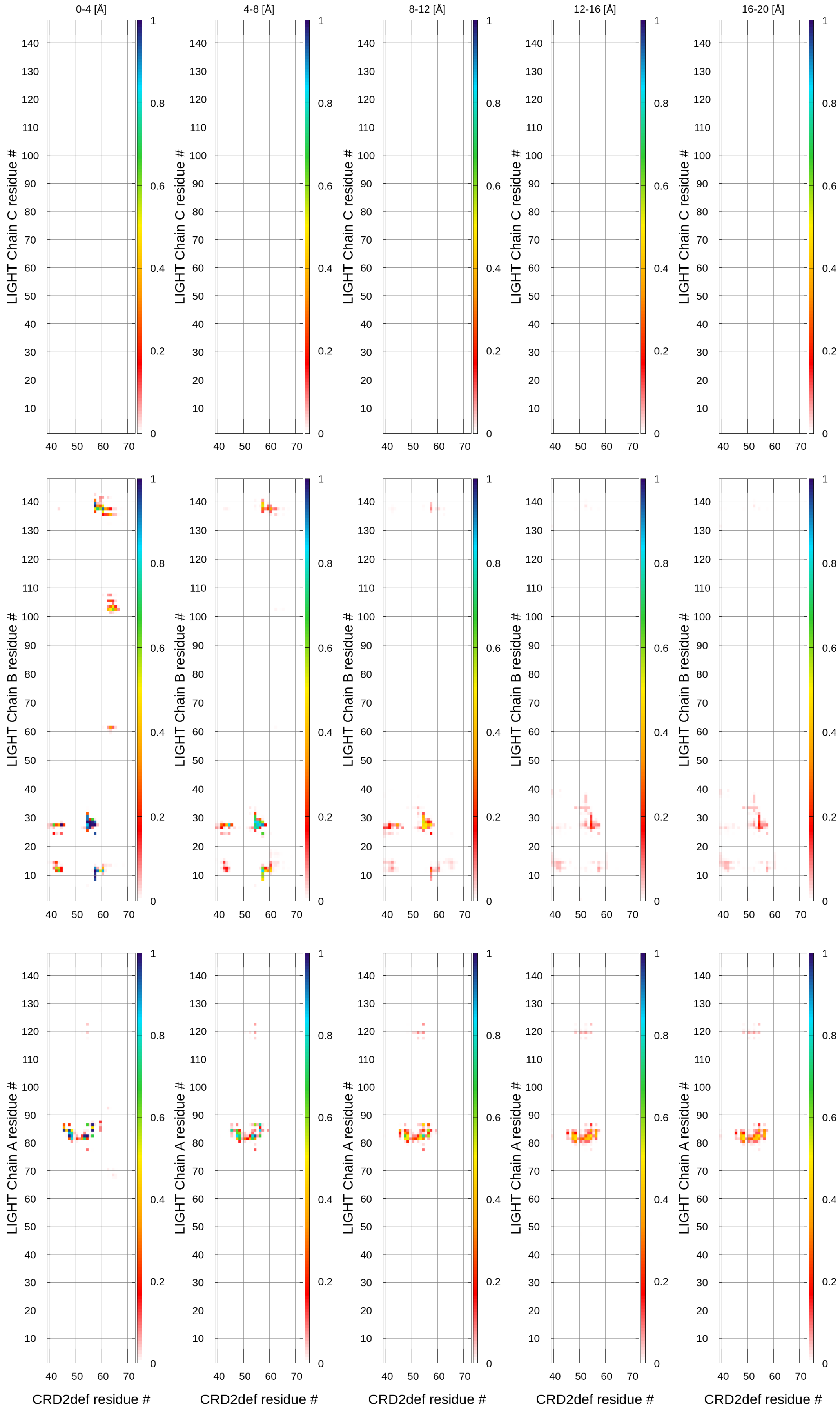

Figure S10: continued: Contact heatmap averaged over 25 SMD simulations for HVEM variant CRD2def with the LIGHT trimer. The values are normalized in the range of increasing extension (relative difference of the distance between centers of mass of HVEM variant and LIGHT trimer) averaged for 4 Å.

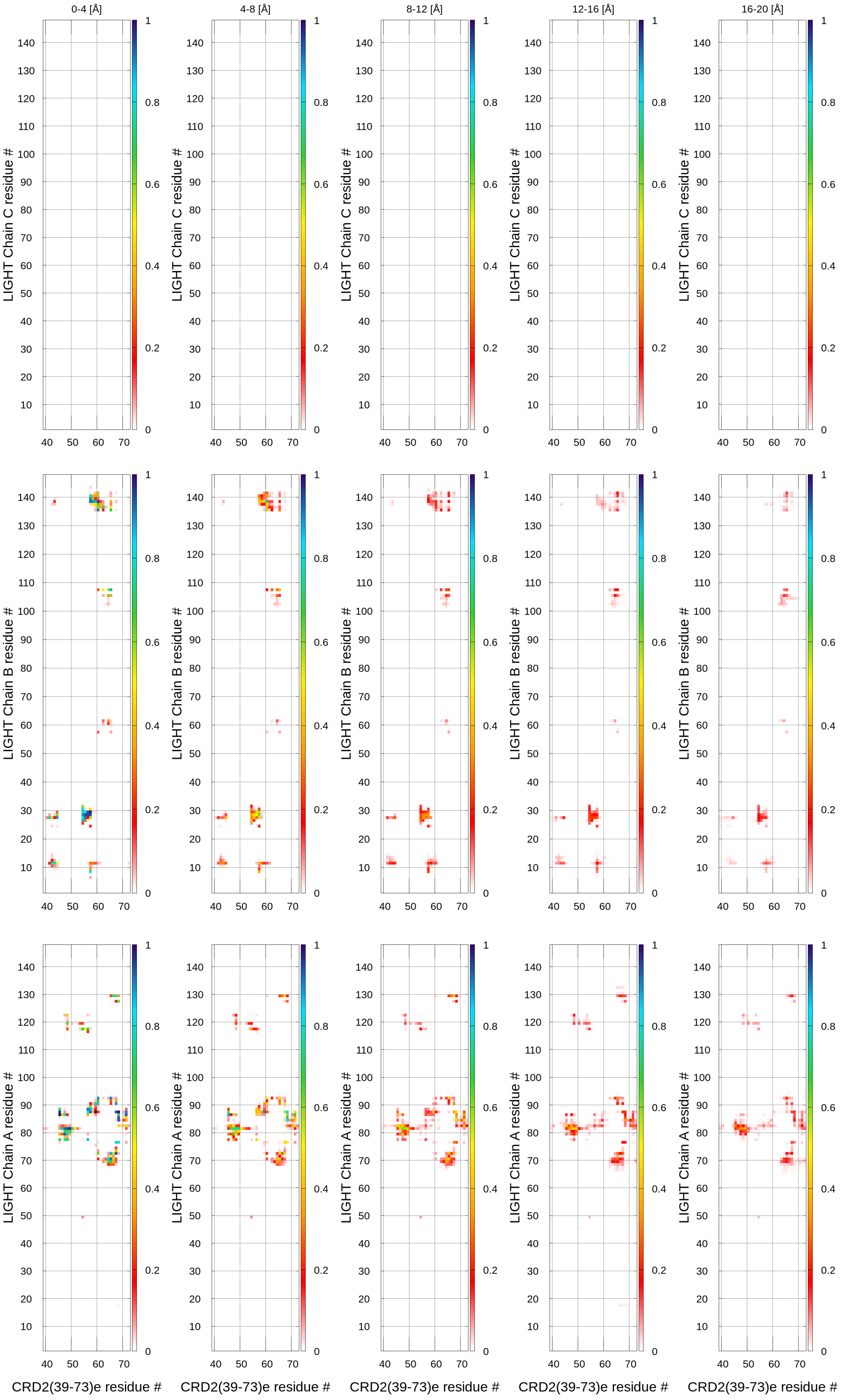

Figure S10: continued: Contact heatmap averaged over 25 SMD simulations for HVEM variant CRD2(39-73)e with the LIGHT trimer. The values are normalized in the range of increasing extension (relative difference of the distance between centers of mass of HVEM variant and LIGHT trimer) averaged for 4 Å.

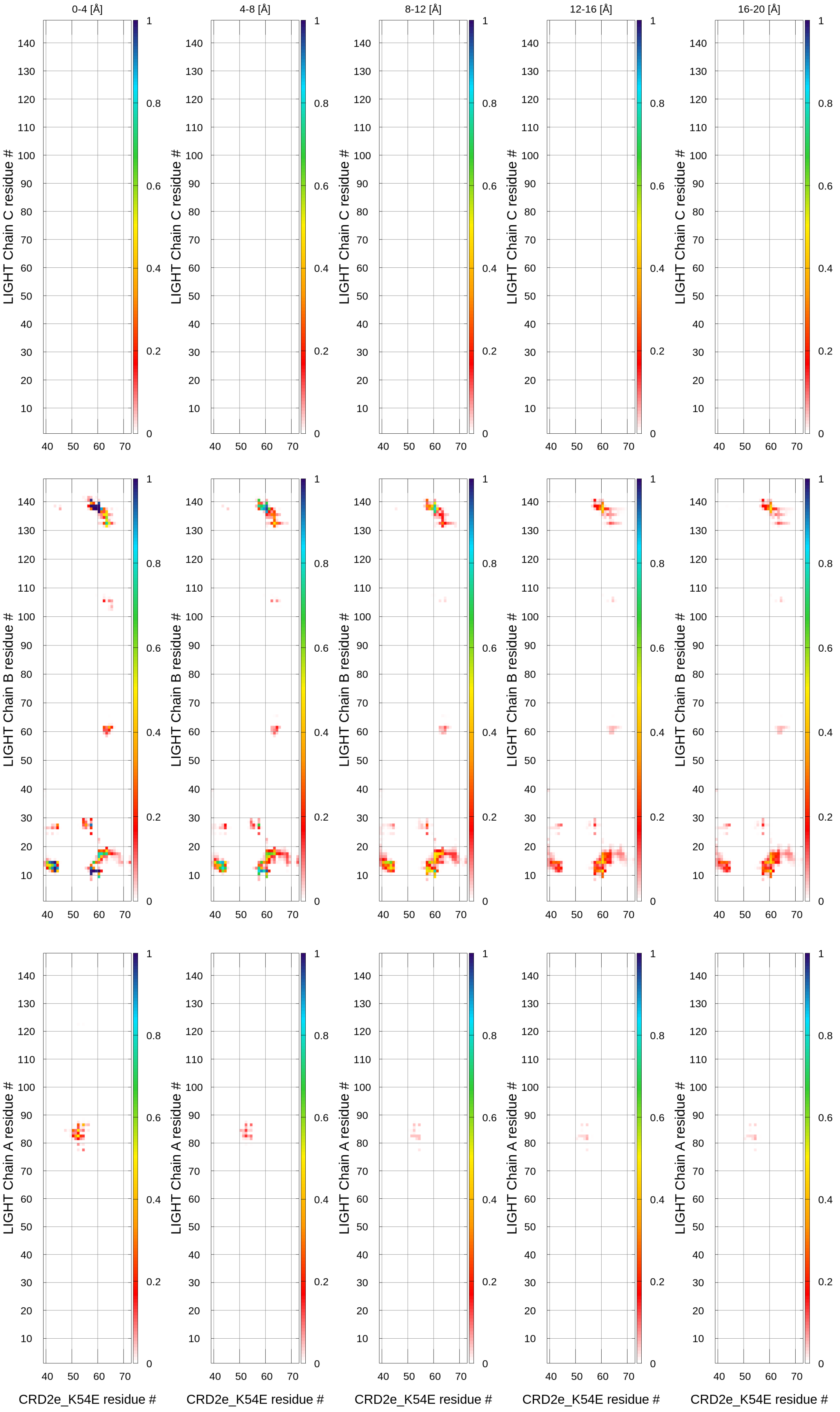

Figure S10: continued: Contact heatmap averaged over 25 SMD simulations for HVEM variant CRD2e_K54E with the LIGHT trimer. The values are normalized in the range of increasing extension (relative difference of the distance between centers of mass of HVEM variant and LIGHT trimer) averaged for 4 Å.

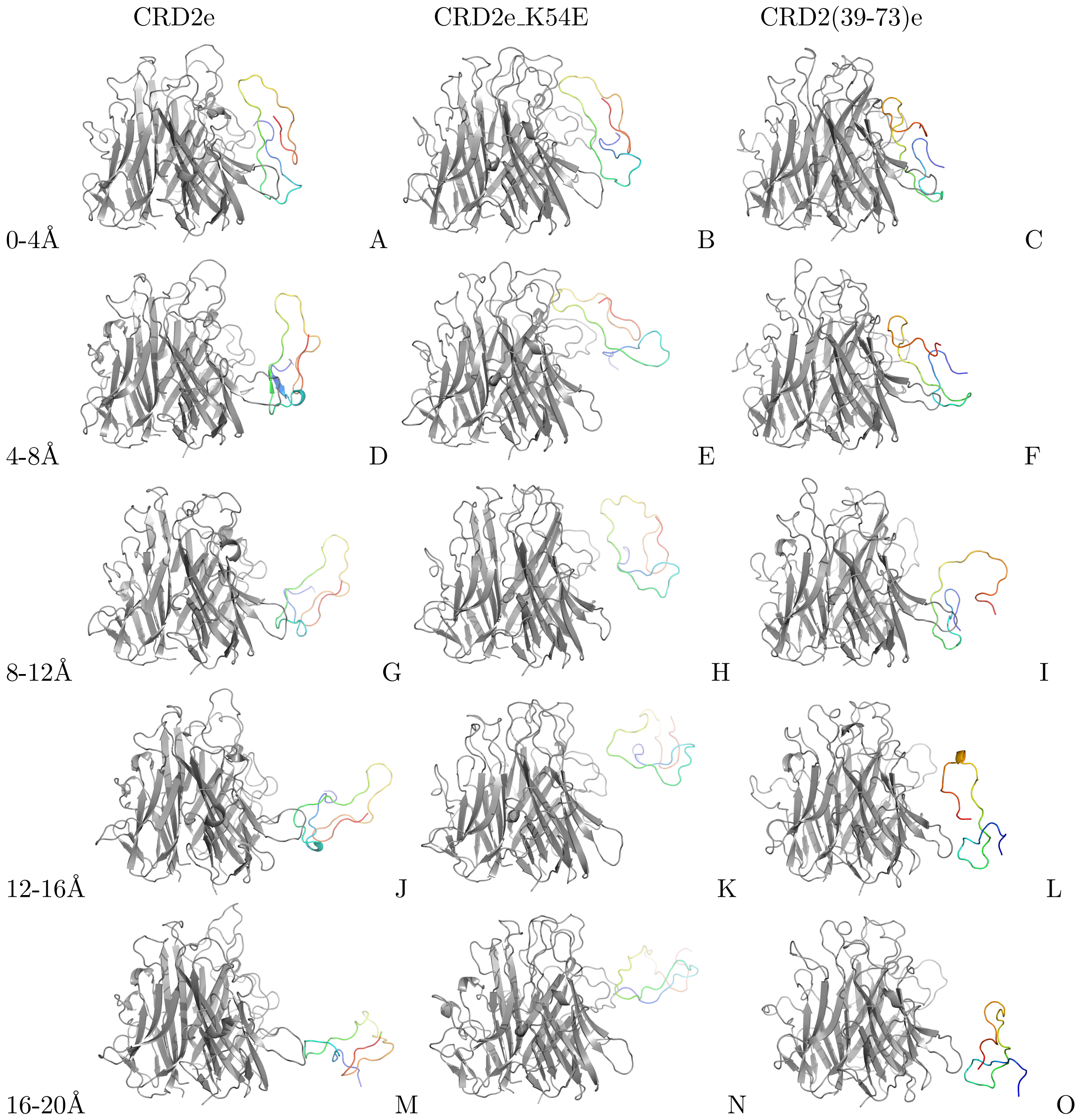

Figure S10: Representative structures upon extending (relative difference of the distance between centers of mass of HVEM variant and LIGHT trimer) averaged for 4 Å.

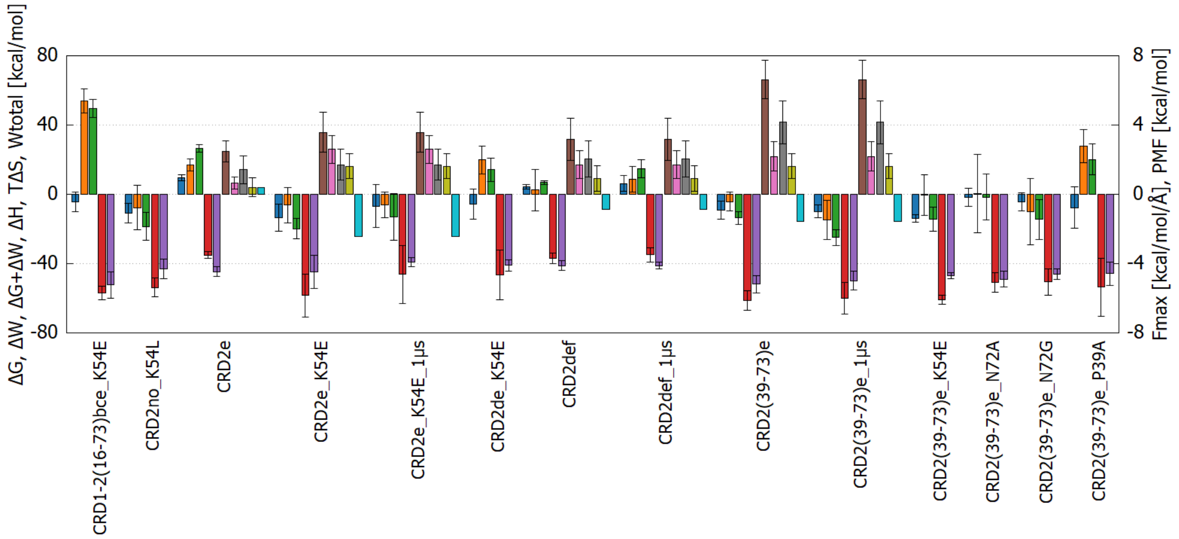

Figure S11: Change of: Binding free energy (ΔG, **blue**), internal work (ΔW, **orange**), summary of Gibbs free energy and internal work (ΔG+ΔW, **green**), enthalpy (ΔH, **red**), entropy (ΔS, **violet**) in 200 ns and 1000 ns long simulations ("1μs" postscript), total work (Wtotal, pulling speed 0.05 m/s **brown**, 0.01 m/s **pink**), force max (Fmax, pulling speed 0.05 m/s **gray**, 0.01 m/s **yellowish green**) and potential mean force (PMF, **cyan**) calculated on SMD basis of eleven selected systems with highest affinity to LIGHT. CRD2e_1μs, due to dissociating, is not shown.

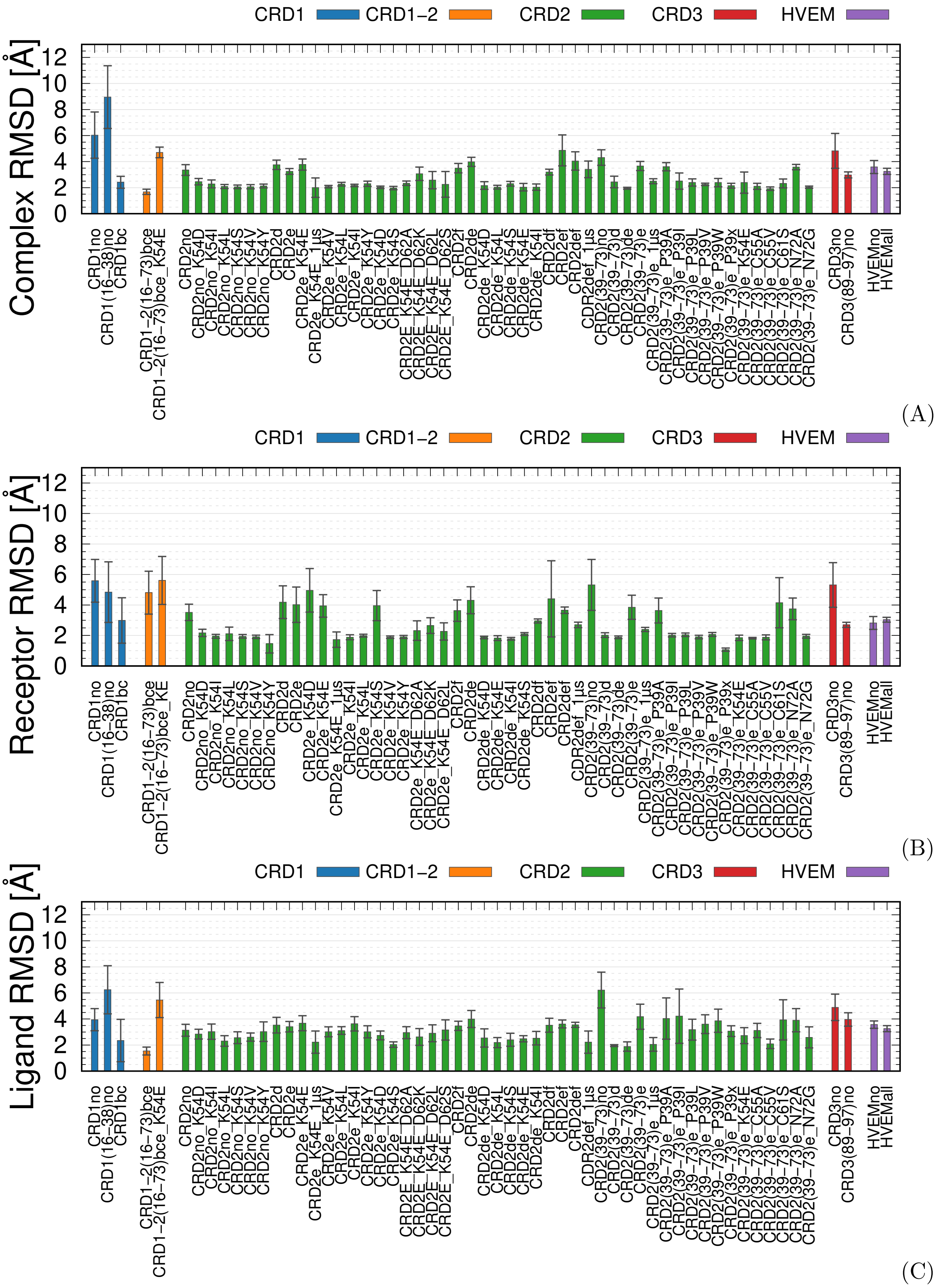

Figure S12: Bar plots of the stable complexes of the structural properties: (A) Complex CαRMSD, (B) Receptor CαRMSD, and (C) Ligand CαRMSD values averaged for 3 trajectories for the last 20 ns of simulation. For the RMSD calculation, an initial conformation adapted from the PDB file was used as a reference.

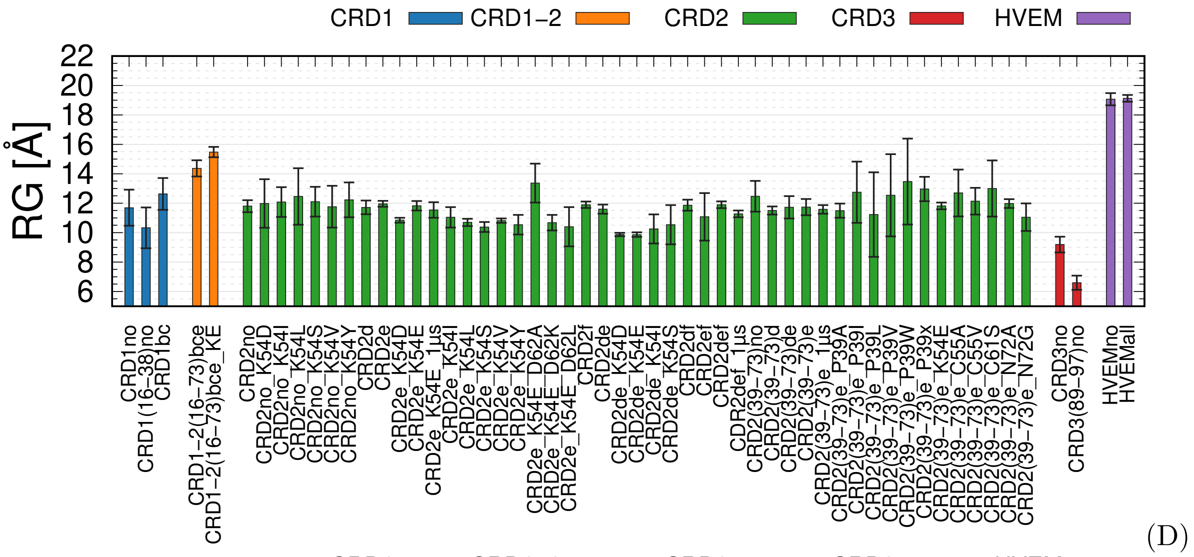

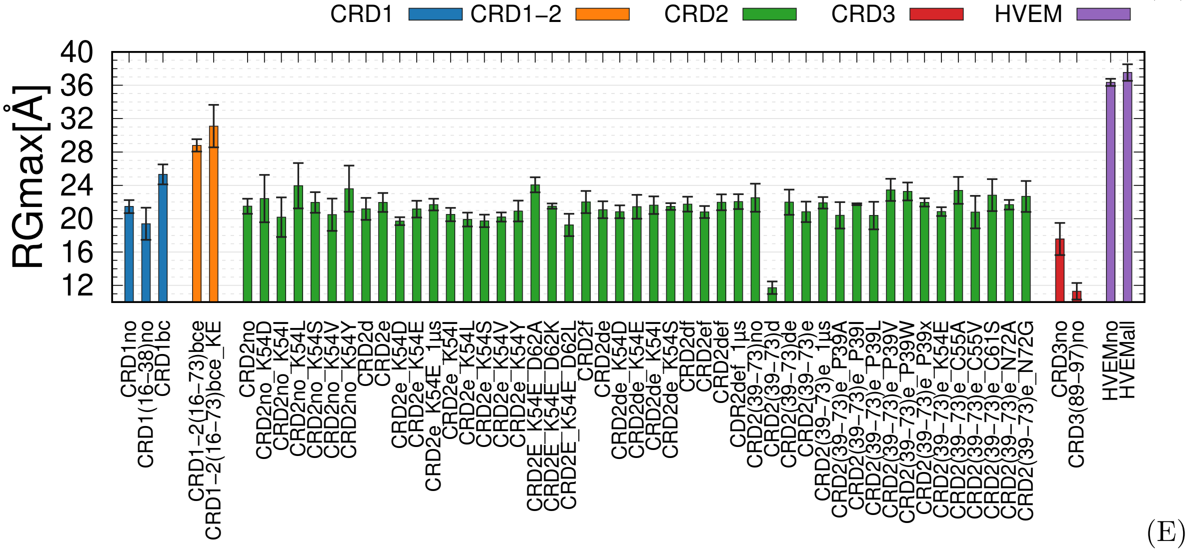

Figure S12: continued: Bar plots of the stable complexes of the structural properties: (D) RG and (E) RGmax values averaged for 3 trajectories for the last 20 ns of simulation. For the RMSD calculation, an initial conformation adapted from the PDB file was used as a reference.

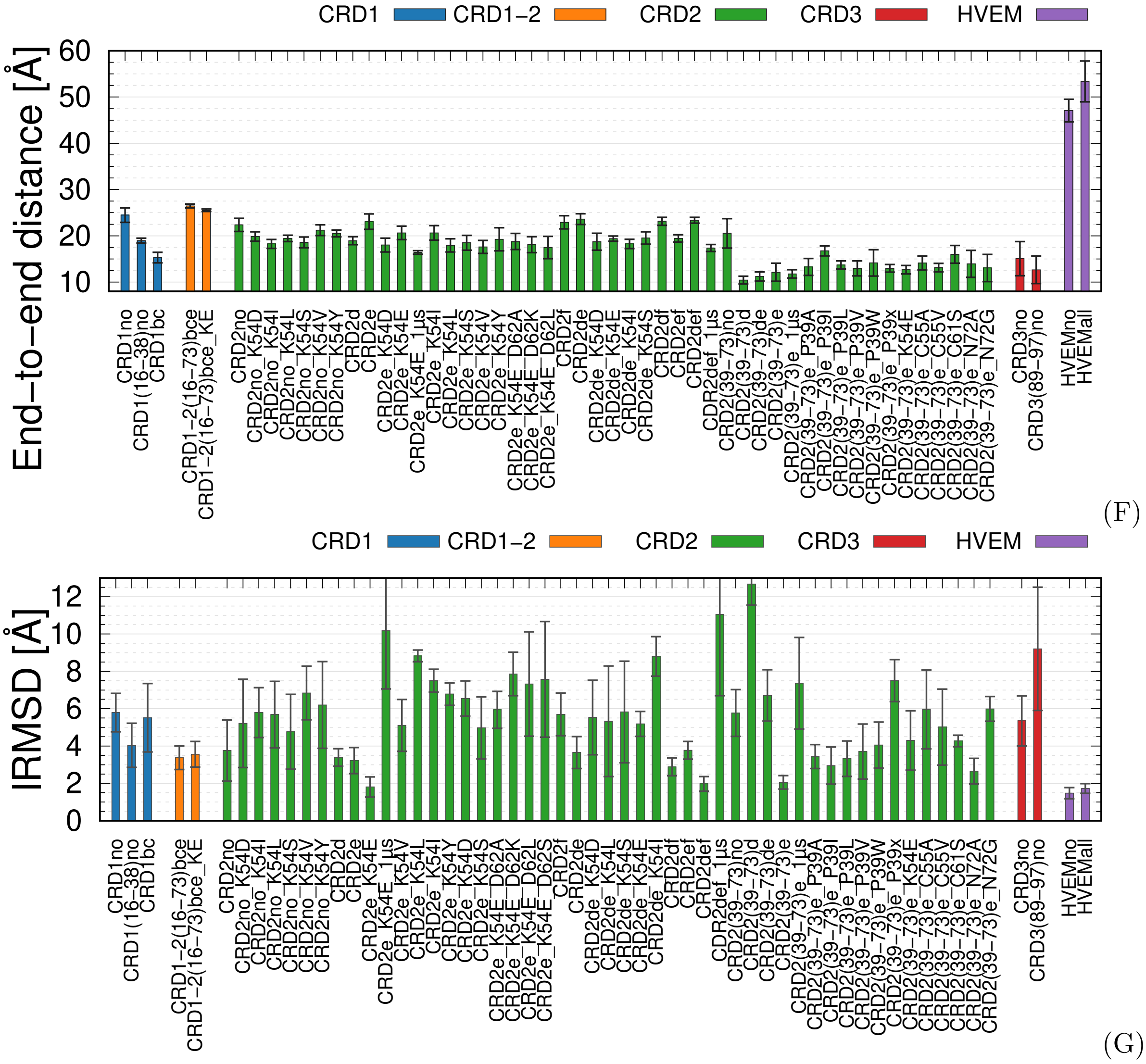

Figure S12: continued: Bar plots of the stable complexes of the structural properties: (F) End-to-end distance and (G) lRMSD values averaged for 3 trajectories for the last 20 ns of simulation. For the RMSD calculation, an initial conformation adapted from the PDB file was used as a reference.

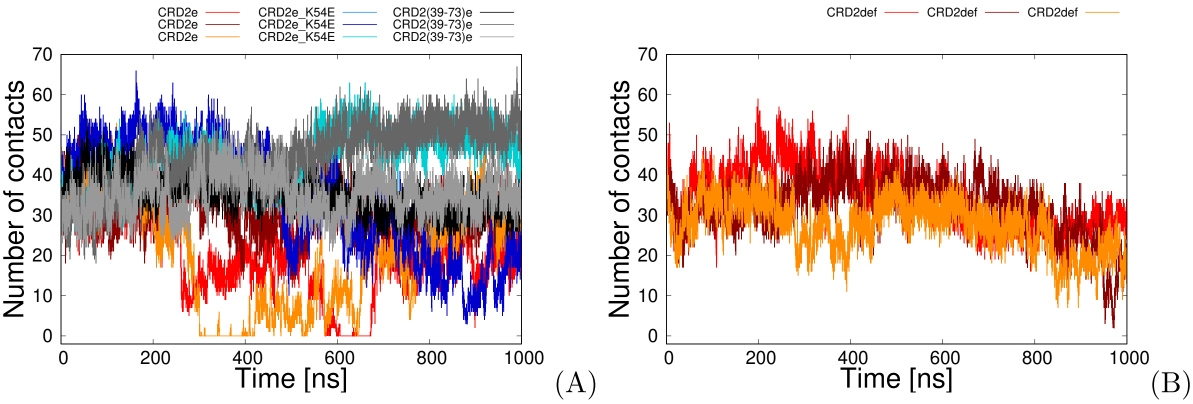

Figure S13: Number of native contacts between HVEM variant and LIGHT trimer as a function of time for 1μs simulations (A): CRD2e (red), CRD2e_K54E (blue), and CRD2(39-73)e (grey); (B): CRD2def (red). Different trajectories are depicted as various color tone.

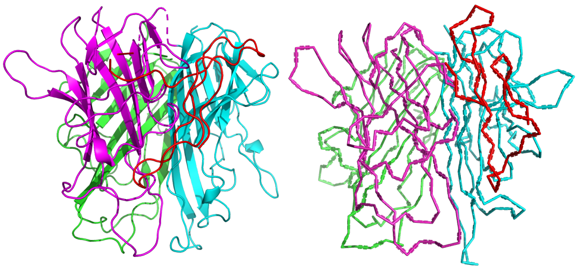

(A)

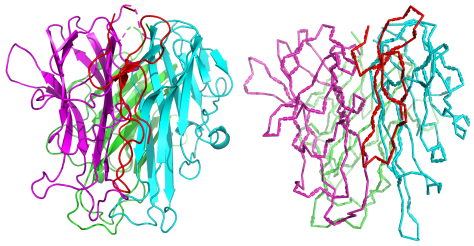

(B)

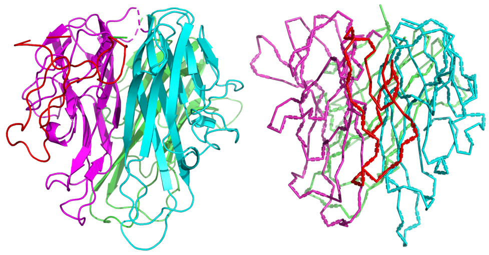

(C)

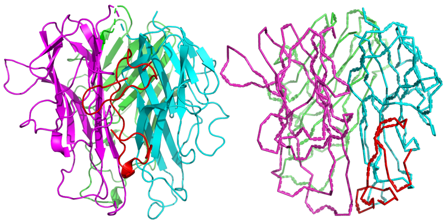

(D)

Figure S14: Comparison between the all-atom dominant structure (left, cartoon representation) with the UNRES-dock cluster with lowest RMSD to respective all-atom structure (right, ribbon representation). Each LIGHT chain is colored green, cyan, magenta and peptide is colored in red for: A) CRD2e (RMSD 4.80Å), B) CRD2def (RMSD 4.39Å), C) CRD2_K54E (RMSD 6.30Å) and D) CRD2(39-73)e (RMSD 4.95Å).
